# Second-line FOLFOX chemotherapy versus active symptom control for advanced biliary tract cancer (ABC-06): a phase 3, open-label, randomised, controlled trial

**DOI:** 10.1016/S1470-2045(21)00027-9

**Published:** 2021-05

**Authors:** Angela Lamarca, Daniel H Palmer, Harpreet Singh Wasan, Paul J Ross, Yuk Ting Ma, Arvind Arora, Stephen Falk, Roopinder Gillmore, Jonathan Wadsley, Kinnari Patel, Alan Anthoney, Anthony Maraveyas, Tim Iveson, Justin S Waters, Claire Hobbs, Safia Barber, W David Ryder, John Ramage, Linda M Davies, John A Bridgewater, Juan W Valle, Advanced Biliary Cancer Working Group

**Affiliations:** a Department of Medical Oncology, The Christie NHS Foundation Trust/Institute of Cancer Sciences, University of Manchester, Manchester, UK; b Manchester Centre for Health Economics, University of Manchester, Manchester, UK; c Manchester Clinical Trials Unit, University of Manchester, Manchester, UK; d Division of Cancer Sciences, University of Manchester, Manchester, UK; e University of Liverpool and Clatterbridge Cancer Centre, Liverpool, UK; f Department of Cancer Medicine, Hammersmith Hospital, Imperial Colllege London, London, UK; g Guy's Cancer, Guy's & St Thomas' NHS Foundation Trust, London, UK; h Department of Hepatobiliary Oncology, University of Birmingham and University Hospitals Birmingham NHS Foundation Trust, Birmingham, UK; i Department of Medical Oncology, University Hospital of Nottingham NHS Trust, University of Nottingham, Nottingham, UK; j Bristol Haematology and Oncology Centre, Bristol, UK; k Department of Medical Oncology, Royal Free NHS Foundation Trust, London, UK; l Weston Park Cancer Centre, Sheffield, UK; m Department of Medical Oncology, Cancer and Haematology Centre, Oxford, UK; n Department of Medical Oncology, Leeds Teaching Hospitals NHS Trust, Leeds, UK; o Department of Medical Oncology, Hull University Teaching Hospitals NHS Trust, Hull, UK; p Department of Gastro-Intestinal Oncology, University Hospital Southampton NHS Foundation Trust, Southampton University, Southampton, UK; q Kent Oncology Centre, Maidstone, UK; r Department of Clinical Oncology, Great Western Hospital, Swindon, UK; s Hampshire Hospitals NHS Foundation Trust, Basingstoke, UK; t UCL Cancer Institute, University College London, London, UK

## Abstract

**Background:**

Advanced biliary tract cancer has a poor prognosis. Cisplatin and gemcitabine is the standard first-line chemotherapy regimen, but no robust evidence is available for second-line chemotherapy. The aim of this study was to determine the benefit derived from second-line FOLFOX (folinic acid, fluorouracil, and oxaliplatin) chemotherapy in advanced biliary tract cancer.

**Methods:**

The ABC-06 clinical trial was a phase 3, open-label, randomised trial done in 20 sites with expertise in managing biliary tract cancer across the UK. Adult patients (aged ≥18 years) who had histologically or cytologically verified locally advanced or metastatic biliary tract cancer (including cholangiocarcinoma and gallbladder or ampullary carcinoma) with documented radiological disease progression to first-line cisplatin and gemcitabine chemotherapy and an Eastern Cooperative Oncology Group performance status of 0–1 were randomly assigned (1:1) centrally to active symptom control (ASC) and FOLFOX or ASC alone. FOLFOX chemotherapy was administered intravenously every 2 weeks for a maximum of 12 cycles (oxaliplatin 85 mg/m^2^, L-folinic acid 175 mg [or folinic acid 350 mg], fluorouracil 400 mg/m^2^ [bolus], and fluorouracil 2400 mg/m^2^ as a 46-h continuous intravenous infusion). Randomisation was done following a minimisation algorithm using platinum sensitivity, serum albumin concentration, and stage as stratification factors. The primary endpoint was overall survival, assessed in the intention-to-treat population. Safety was also assessed in the intention-to-treat population. The study is complete and the final results are reported. This trial is registered with ClinicalTrials.gov, NCT01926236, and EudraCT, 2013-001812-30.

**Findings:**

Between March 27, 2014, and Jan 4, 2018, 162 patients were enrolled and randomly assigned to ASC plus FOLFOX (n=81) or ASC alone (n=81). Median follow-up was 21·7 months (IQR 17·2–30·8). Overall survival was significantly longer in the ASC plus FOLFOX group than in the ASC alone group, with a median overall survival of 6·2 months (95% CI 5·4–7·6) in the ASC plus FOLFOX group versus 5·3 months (4·1–5·8) in the ASC alone group (adjusted hazard ratio 0·69 [95% CI 0·50–0·97]; p=0·031). The overall survival rate in the ASC alone group was 35·5% (95% CI 25·2–46·0) at 6 months and 11·4% (5·6–19·5) at 12 months, compared with 50·6% (39·3–60·9) at 6 months and 25·9% (17·0–35·8) at 12 months in the ASC plus FOLFOX group. Grade 3–5 adverse events were reported in 42 (52%) of 81 patients in the ASC alone group and 56 (69%) of 81 patients in the ASC plus FOLFOX group, including three chemotherapy-related deaths (one each due to infection, acute kidney injury, and febrile neutropenia). The most frequently reported grade 3–5 FOLFOX-related adverse events were neutropenia (ten [12%] patients), fatigue or lethargy (nine [11%] patients), and infection (eight [10%] patients).

**Interpretation:**

The addition of FOLFOX to ASC improved median overall survival in patients with advanced biliary tract cancer after progression on cisplatin and gemcitabine, with a clinically meaningful increase in 6-month and 12-month overall survival rates. To our knowledge, this trial is the first prospective, randomised study providing reliable, high-quality evidence to allow an informed discussion with patients of the potential benefits and risks from second-line FOLFOX chemotherapy in advanced biliary tract cancer. Based on these findings, FOLFOX should become standard-of-care chemotherapy in second-line treatment for advanced biliary tract cancer and the reference regimen for further clinical trials.

**Funding:**

Cancer Research UK, StandUpToCancer, AMMF (The UK Cholangiocarcinoma Charity), and The Christie Charity, with additional funding from The Cholangiocarcinoma Foundation and the Conquer Cancer Foundation Young Investigator Award for translational research.

**Research in context**

**Evidence before this study**

A previous systematic review by this group published in 2014 evaluated the level of evidence for the use of second-line chemotherapy for patients with advanced biliary tract cancer. As part of this systematic review, we searched MEDLINE using the search terms “[(((biliary tract AND chemotherapy AND second) OR (biliary tract AND chemotherapy AND refractory)) OR (gallbladder AND chemotherapy AND refractory)) OR (cholangiocarcinoma AND chemotherapy AND refractory)]”, with no publication date or language restrictions. Abstracts of the Proceedings of the Annual Meeting of the American Society of Clinical Oncology, the European Society of Medical Oncology Congress since 2002, and the annual World Gastrointestinal Congress since 2006 were searched manually. Up to December, 2013, 25 studies were identified, which were mainly phase 2 clinical trials and retrospective series. No randomised phase 3 studies were identified. When this search of the literature was updated in December, 2020, phase 3 trial-based evidence supporting the role of cytotoxic chemotherapy in this setting was still nonexistent. Therefore, before this study, it was not possible to reliably determine the real benefit from second-line chemotherapy in this setting. Moreover, there was insufficient evidence to recommend a specific second-line chemotherapy schedule in advanced biliary tract cancer.

**Added value of this study**

To our knowledge, ABC-06 is the first trial to compare active symptom control alone with active symptom control combined with FOLFOX chemotherapy following progression on first-line treatment with cisplatin and gemcitabine for patients with advanced biliary tract cancer. The study met its primary endpoint and confirmed that patients who received FOLFOX chemotherapy had a longer overall survival, with a clinically meaningful increase in 6-month and 12-month overall survival rates, than those who received active symptom control alone. This outcome was achieved with an acceptable toxicity profile. Ongoing quality of life, health economics, and translational analyses of ABC-06 will help us to understand both the impact on patients' experience and the cost-benefit of FOLFOX chemotherapy in this setting, and the mechanisms of primary and secondary resistance to this treatment.

**Implications of all the available evidence**

To our knowledge, this study is the first prospective, adequately powered, randomised trial exploring the role of chemotherapy in this setting, thus providing reliable high-quality evidence to allow an informed discussion with patients of the potential benefits and risks from second-line FOLFOX chemotherapy in advanced biliary tract cancer. Based on the results of ABC-06, FOLFOX should become standard-of-care chemotherapy in second-line treatment for advanced biliary tract cancer and the reference regimen for further clinical trials.

## Introduction

Biliary tract cancer is a term that includes cholangiocarcinoma (either intrahepatic or extrahepatic in origin) and cancers of the gallbladder and ampulla of Vater. These uncommon cancers arising from the biliary tract account for less than 1% of all cancers worldwide.^1^ However, its incidence is increasing, primarily due to a rising incidence of intrahepatic cholangiocarcinoma. The prognosis is poor, with an all-stage 5-year overall survival of less than 20%.^1^ Patients are rarely diagnosed with early-stage disease and therefore curative surgery and adjuvant therapy is only feasible for a small proportion of patients.

The ABC-02 study established cisplatin and gemcitabine as first-line therapy for patients with advanced biliary tract cancer^2^ and remains the current standard of care. Randomised studies during the past decade have failed to show an improvement in survival with the addition of biological therapies (EGFR or VEGF inhibitors); intensification of chemotherapy is under evaluation (eg, gemcitabine, cisplatin, and nab-paclitaxel; cisplatin, gemcitabine, and S-1; and FOLFIRINOX [oxaliplatin, leucovorin, irinotecan, and fluorouracil]).^3^, ^4^, ^5^

The role of second-line chemotherapy after progression on cisplatin and gemcitabine remains unclear, with no prospective randomised trials reported so far.^6^, ^7^ Patients with advanced biliary tract cancer often experience a rapid decline in performance status following progression on first-line chemotherapy, and only 15–25% receive second-line therapy.^2^, ^8^, ^9^ Some studies suggest that second-line chemotherapy might be of value for patients with a good performance status;^8^, ^10^, ^11^, ^12^ however, this theory is subject to selection bias and has not been explored in a randomised study, and no consensus exists regarding the optimum regimen.

Three groups of agents have broadly shown activity in biliary tract cancer in retrospective and prospective trials: gemcitabine, fluoropyrimidines, and platinum agents.^13^ Since the sensitivity to platinum agents in these malignancies is well described,^2^ together with the fact that switching to a fluoropyrimidine-based schedule after progression on first-line gemcitabine-based chemotherapy is considered appropriate in similar scenarios,^14^ it was anticipated that a fluorouracil and platinum doublet (such as oxaliplatin and fluorouracil in FOLFOX, which comprises leucovorin, fluorouracil, and oxaliplatin) was most likely to be effective. Existing phase 2 and retrospective data also supported this choice.^6^

Novel molecular targets such as fibroblast growth factor receptor-2 (*FGFR2*) fusions and isocitrate dehydrogenase-1 (*IDH1*) mutations have been identified as promising within phase 2 and phase 3 trials, respectively, showing benefit in the second-line setting for a selected population of patients harbouring such aberrations (mainly those with intrahepatic cholangiocarcinoma, with approximately 15% prevalence of each).^15^, ^16^, ^17^, ^18^ However, active symptom control (ASC), including early identification and management of biliary tract and cancer-related complications and symptom management arising from tumour progression, is the current standard of care for most patients diagnosed with advanced biliary tract cancer who have progressed following first-line chemotherapy, especially in the absence of targetable molecular alterations.^6^

The ABC-06 study aimed to determine if patients with advanced biliary tract cancer benefit from the addition of second-line FOLFOX to ASC, following progression to previous first-line treatment with cisplatin and gemcitabine.

## Methods

### Study design and participants

The ABC-06 clinical trial was a phase 3, open-label, randomised controlled study conducted under the auspices of the UK National Cancer Research Institute Upper Gastrointestinal Cancer Studies Group. Patients were recruited across 20 centres with expertise in managing biliary tract cancer in the UK (appendix p 17).

Patients were eligible if they were aged 18 years or older and had histologically or cytologically verified locally advanced or metastatic biliary tract cancer (including cholangiocarcinoma, gallbladder carcinoma, and ampullary carcinoma) with documented radiological disease progression to previous first-line cisplatin and gemcitabine chemotherapy. Any other form of first-line systemic chemotherapy or additional line of first-line chemotherapy (including rechallenge with cisplatin and gemcitabine) was not allowed. Patients who had been started on first-line cisplatin and gemcitabine for whom the cisplatin was stopped due to toxicity (with continuation of gemcitabine) were eligible.

A maximum of 6 weeks was allowed between disease progression to first-line treatment and the start of second-line chemotherapy as part of the ABC-06 trial. All patients had to have an Eastern Cooperative Oncology Group (ECOG) performance status of 0–1, life expectancy of longer than 3 months, and adequate haematological, renal, and hepatic function, with no evidence of ongoing infection or inadequate biliary drainage. Patients with clinical evidence of metastatic disease to the brain and those with clinically significant cardiovascular disease were excluded. Full details of the patient inclusion and exclusion criteria are in the protocol (see appendix).

Trial data were collected and monitored at each site, and underwent quality control and analysis at the Manchester Clinical Trials Unit (CTU; Manchester, UK). The study was sponsored by The Christie NHS Foundation Trust and conducted in accordance with the principles of Good Clinical Practice and the Declaration of Helsinki. The study protocol was approved by a research ethics committee and all patients were required to provide written, informed consent before any trial-related investigations or treatment took place.

### Randomisation and masking

This was an open-label study, with no masking. Patients were randomly assigned (1:1) to ASC plus FOLFOX or ASC alone. Researchers contacted a central telephone number whereupon CTU staff used a computer system employing a minimisation algorithm over the margins of three factors to determine the allocation. Allocations were made with a probability of 0·75 to the group that would yield improved balance or 0·5 if balance scores were tied. Allocations were revealed to the CTU staff member only after all the participant details had been committed to the system. The allocation was then relayed verbally to the caller and an automated confirmation e-mail was sent to the recruiting site.

Platinum sensitivity (sensitive *vs* refractory or resistant), serum albumin concentration (<3·5 mg/L *vs* ≥3·5 mg/L), and disease stage (locally advanced *vs* metastatic) were used as stratification factors.^8^, ^11^, ^19^, ^20^ The definition of platinum sensitivity was derived from first-line cisplatin and gemcitabine data based on the difference between median progression-free survival and time on chemotherapy from the ABC-02 clinical trial (approximately 3 months).^2^ Platinum sensitivity was defined as sensitive (progression after 90 days of day 1 of the last cycle of first-line cisplatin and gemcitabine), refractory (progression during first-line cisplatin and gemcitabine), or resistant (progression within the first 90 days after completion of day 1 of the last cycle of first-line cisplatin and gemcitabine). Disease stage was defined as locally advanced versus metastatic as per the American Joint Committee on Cancer staging (version 7),^21^ according to which intrahepatic cholangiocarcinoma with multifocal or satellite liver lesions or metastases were classified as locally advanced in the absence of extrahepatic metastatic disease. Although performance status^22^ is a widely recognised prognostic factor in advanced biliary tract cancer,^8^, ^11^, ^12^, ^19^ since only patients with ECOG performance status 0–1 were eligible, serum albumin concentration was included as a stratification factor instead.^11^, ^23^

### Procedures

ASC consisted of early identification and treatment of biliary-related complications and cancer-related symptom management; it could include (and was not limited to) the following as per requirements of individual patients: biliary drainage, antibiotics, analgesia, steroids, anti-emetics, other palliative treatment for symptom control, palliative radiotherapy (eg, for painful bone metastases), and transfusion of blood products.

Patients allocated to the ASC plus FOLFOX group also received FOLFOX chemotherapy every 2 weeks for a maximum of 12 cycles. Treatment took place over 2 days, and consisted of oxaliplatin 85 mg/m^2^ (in 250–500 mL of 5% glucose; 2-h intravenous infusion), L-folinic acid 175 mg (or folinic acid 350 mg; 2-h intravenous infusion concurrently with oxaliplatin infusion), and fluorouracil 400 mg/m^2^ (5–10 min bolus) completed on day 1, and fluorouracil 2400 mg/m^2^ as continuous intravenous infusion starting on day 1 and finishing on day 2. At the investigator's discretion, fluorouracil could be started at 80% of the full dose (with full-dose oxaliplatin) for patients older than 70 years. The dose of oxaliplatin could be adjusted to 65 mg/m^2^ if the creatinine clearance was 30–60 mL/min. It was recommended that the body surface area be capped at 2·2 m^2^ for this study, and to be recalculated in case of weight variation of greater than 5%. From cycle 2 onwards, patients were required to have a neutrophil count of at least 1·5 × 10^9^ per L and platelet count of at least 75 × 10^9^ per L for chemotherapy to proceed. Anti-emetic and supportive medication was administered following local protocols. Chemotherapy continued (in the absence of disease progression, intolerable toxicity, or patient choice to withdraw) up to a maximum of 12 cycles (6 months) and patients continued with ASC visits every 4 weeks thereafter. A maximum of two dose reduction levels per drug were allowed: level −1 represented a 20% reduction from the full initial dose of each drug while level −2 represented a 50% reduction. If level −2 was not adequately tolerated for a specific drug, that drug was discontinued. If oxaliplatin was discontinued due to toxicity, treatment could continue with fluorouracil and folinic acid or L-folinic acid alone if deemed appropriate by the local investigator. In that case, the dose per m^2^ of fluorouracil could be increased as per local practice at the discretion of the investigator. When a treatment delay was needed because of toxicity, the patient was evaluated weekly and the drug restarted if the toxicity recovered to grade 1 or lower. If there were more than 28 days of treatment delay, the patient received no further protocol-mandated treatment. Patients withdrawn from protocol treatment who agreed to continue on study were still followed up until study end and details of further treatment given outside the trial were recorded.

All patients were seen every 4 weeks for ASC, and those receiving chemotherapy were seen every 2 weeks for FOLFOX chemotherapy. During these clinic visits, physical examination, assessment of ECOG performance status, symptom monitoring, reviewing of concomitant medication, and assessment of liver and renal function with full blood count was done. Adverse events were collected at every clinic visit and were classified according to the Common Terminology Criteria for Adverse Events version 4.03, with causality assigned by treating investigators or delegated clinicians. Tumour marker (CA19-9, carcinoembryonic antigen, and CA125) and C-reactive protein assessments were done at baseline and at every follow-up appointment.

Patients in the ASC plus FOLFOX group underwent radiological tumour evaluation by CT (and optional MRI if clinically indicated) 12 weeks after the start of chemotherapy, at the end of chemotherapy, and every 3 months thereafter until documentation of disease progression. All radiological evaluations were investigator assessed, with no central review. Patients assigned to ASC alone did not have regular radiological tumour evaluation; imaging was allowed as clinically indicated. Biliary tract obstruction in itself did not constitute evidence of disease progression. Disease response to therapy and progression was defined according to Response Evaluation Criteria in Solid Tumors (RECIST) version 1.1 in the ASC plus FOLFOX group undergoing radiological follow-up.^24^

In patients with disease progression after ASC plus FOLFOX, subsequent treatment was administered at the treating clinician's discretion, including experimental therapies in the context of phase 1 clinical trials. Patients assigned to ASC alone were permitted to receive treatment with experimental therapies in the context of phase 1 clinical trials.

Quality of life and health status questionnaires (European Organisation for Research and Treatment of Cancer [EORTC] Quality of Life Questionnaire [QLQ] 30,^25^ EORTC QLQ-BIL21,^26^, ^27^ and EQ-5D^28^) were used for quality-of-life analyses, the results of which will be reported separately.

Archival tissue and prospective blood samples (whole blood, serum, and plasma) were collected for all patients at baseline and at 12 weeks, and were collected upon disease progression for patients in the ASC plus FOLFOX group for future translational research. For patients with HIV, hepatitis C, or other transmissible human diseases, blood sample collection did not take place. All samples were stored in the Manchester Cancer Research Centre Biobank (Manchester, UK; Human Tissue Authority licence number 30004; South Manchester Research Ethics Committee reference number 18/NW/0092).

The independent data monitoring committee did an early safety review on Sept 30, 2015, of the first 20 patients in the ASC plus FOLFOX group who had completed one cycle. Additionally, because of the concern that patients' disease might be platinum resistant after having progressed on a previous platinum regimen, the committee was specifically asked at the same review to consider whether a change in regimen to a non-platinum containing regimen was required in the event of futility. The committee concluded that no change was required and the trial could proceed.

The end of the trial was defined as completion of 12 months after the date of enrolment of the last patient included, or after the death of all the patients, whichever happened first. Patients were able to withdraw consent from the trial at any time if they wished.

### Outcomes

The primary endpoint was overall survival, defined as the time from randomisation to death from any cause. Secondary endpoints were progression-free survival (time between randomisation and radiological disease progression or death of any cause, whichever occurred first) and radiological response as per RECIST version 1.1 for the ASC plus FOLFOX group only; and assessment of adverse events, quality of life, and health economics in both groups. Outcomes and toxicity findings are provided in this Article; data on quality of life and health economics will be reported separately.

### Statistical analysis

The study was powered to show a benefit in overall survival with the addition of FOLFOX to ASC in the intention-to-treat population. 148 death events were required for a hypothesised hazard ratio (HR) of 0·63 with 80% power and 5% two-sided α; since minimal (<3%) loss to follow-up was envisaged, the required sample size was 162 patients. At the time of the study design, the assumed 12-month overall survival rate for patients assigned to ASC alone was 10% (derived from the 24-month overall survival rate from the cisplatin and gemcitabine group in the ABC-02 study,^2^ given that the first 12 months were taken up with first-line effect), and the median overall survival was assumed to be 4 months (derived from the difference between overall survival [12 months] and progression-free survival [8 months] in the ABC-02 study^2^). The hypothesised HR was equivalent to an increase in median overall survival from 4 months to 6·4 months.

The study was expected to recruit across 20 centres and recruitment was expected to be completed in 28 months (from February, 2014, to August, 2016). Because of slower than anticipated recruitment, the study period was extended to allow the required sample size to be reached (protocol version 6.0; July 26, 2017). From the start of recruitment to final analysis of the findings, eight protocol amendments were submitted and approved (appendix p 2). None of these amendments affected the sample size or primary or secondary endpoints. The final study protocol (version 7.0) is available in the appendix.

Analysis of the primary endpoint (overall survival) was done with multivariable Cox regression adjusted for the stratification factors (platinum sensitivity, serum albumin concentration, and disease stage); HRs for each stratification factor are also provided, adjusted by the other stratification factors and treatment group. Proportional hazard assumptions were assessed with plots of Schoenfeld residuals and one-step tests on trend. No interim analysis of the primary endpoint was planned. Median overall survival, progression-free survival, and survival rates at 3 months (progression-free survival) and at 6 and 12 months (both progression-free survival and overall survival) were derived from Kaplan-Meier estimates. Surviving patients were censored at their time of last follow-up. Radiological response data were summarised by best recorded response, complete response, partial response, stable disease, progressive disease, or death. Efficacy and safety analyses were done in the intention-to-treat population.

Prespecified subgroup analyses by stratification factors for overall survival and progression-free survival were done, together with post-hoc subgroup analyses by primary tumour site and ECOG performance status. A post-hoc sensitivity analysis for overall survival using the stratification categories provided by local investigators at the time of randomisation was also done.

Two-sided p<0·05 was considered significant. Stata (version 15.1) was used for statistical analysis. The final database extraction for analysis was done on April 3, 2020. The study was registered as an interventional randomised open-label trial with ClinicalTrials.gov, NCT01926236, and EudraCT, 2013-001812-30.

### Role of the funding source

The funders for this academic investigator-initiated study provided input in the form of peer review to ensure patient acceptability but had no role in study design in conception. The study sponsor (The Christie NHS Foundation Trust) provided regulatory and governance oversight with no direct involvement in design or data collection, analysis, or interpretation. The sponsor was not involved in the writing of the study report, although has approved the final version.

## Results

**Figure 1:**
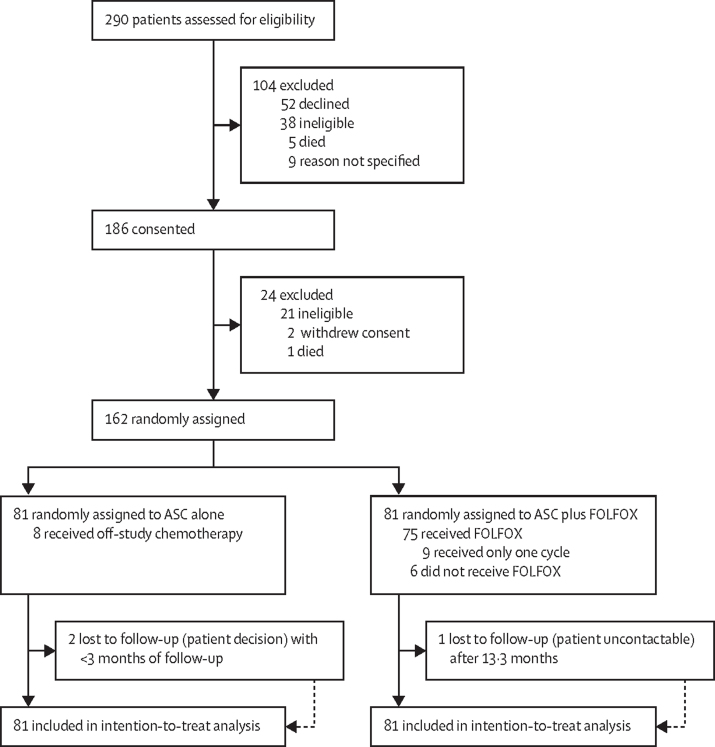
Trial profile ASC=active symptom control. FOLFOX=folinic acid, fluorouracil, and oxaliplatin.

Between March 27, 2014, and Jan 4, 2018, 290 patients were assessed for eligibility, of whom 162 patients from 20 sites in the UK were randomly assigned to ASC alone (n=81) or ASC plus FOLFOX (n=81; figure 1). The end of the follow-up of the last patient recruited, and thus the end of the study, was reached on Jan 4, 2019. At data cutoff, the median follow-up was 21·7 months (IQR 17·2–30·8).

In all enrolled patients, the primary tumour sites were intrahepatic cholangiocarcinoma (in 72 [44%] of 162 patients), extrahepatic cholangiocarcinoma (45 [28%]), gallbladder (34 [21%]), and ampullary cancer (11 [7%]). Baseline characteristics were generally well balanced between study groups (table 1).

**Table 1:** Baseline characteristics

		**ASC alone group (n=81)**	**ASC plus FOLFOX group (n=81)**
Sex			
	Female	44 (54%)	38 (47%)
	Male	37 (46%)	43 (53%)
Age, years			
	Median	65 (59–72)	65 (59–72)
	Range	26–81	26–84
Platinum sensitivity*			
	Resistant or refractory†	47 (58%)	54 (67%)
	Sensitive	34 (42%)	27 (33%)
Albumin*			
	<35 g/L	21 (26%)	19 (23%)
	≥35 g/L	60 (74%)	62 (77%)
Disease stage*			
	Locally advanced	15 (19%)	14 (17%)
	Metastatic	66 (81%)	67 (83%)
Tumour site			
	Intrahepatic	38 (47%)	34 (42%)
	Extrahepatic	19 (23%)	26 (32%)
	Gallbladder	17 (21%)	17 (21%)
	Ampulla	7 (9%)	4 (5%)
Histology			
	Adenocarcinoma	74 (91%)	73 (90%)
	Other‡	7 (9%)	8 (10%)
Grade of differentiation			
	Well	5 (6%)	9 (11%)
	Moderately	41 (51%)	37 (46%)
	Poorly	11 (14%)	9 (11%)
	Not specified	23 (28%)	26 (32%)
	Missing	1 (1%)	0
ECOG performance status			
	0	28 (35%)	25 (31%)
	1	52 (64%)	55 (68%)
	Missing	1 (1%)	1 (1%)
Had previous surgery		38 (47%)	34 (42%)
Previous cisplatin and gemcitabine			
	Duration, months	4·8 (2·9–5·3)	4·9 (2·8–5·5)
	≥6 months	6 (7%)	13 (16%)§
Baseline CA19.9 (U/mL)¶		443 (46–5714)	162 (25–1903)
Baseline carcinoembryonic antigen (U/mL)¶		6 (3–16)	6 (3–24)
Baseline CA125 (U/mL)¶		42 (20–168)	52 (21–159)

Data are n (%) or median (IQR). ASC=active symptom control. FOLFOX= folinic acid, fluorouracil, and oxaliplatin. ECOG=Eastern Cooperative Oncology Group.

* Stratification factors.

† 26 patients in the ASC alone group and 36 patients in the ASC plus FOLFOX group had platinum refractory disease. 21 patients in the ASC alone group and 18 patients in the ASC plus FOLFOX group had platinum-resistant disease.

‡ Other included squamous, adenosquamous, and not specified.

§ Five patients were platinum sensitive, seven platinum resistant, and one platinum refractory.

¶ Baseline tumour marker data were available for 67 (ASC alone) and 68 (ASC plus FOLFOX) patients for CA19.9, 76 (ASC alone) and 76 (ASC plus FOLFOX) patients for carcinoembryonic antigen, and 71 (ASC alone) and 72 (ASC plus FOLFOX) patients for CA125.

**Figure 2:**
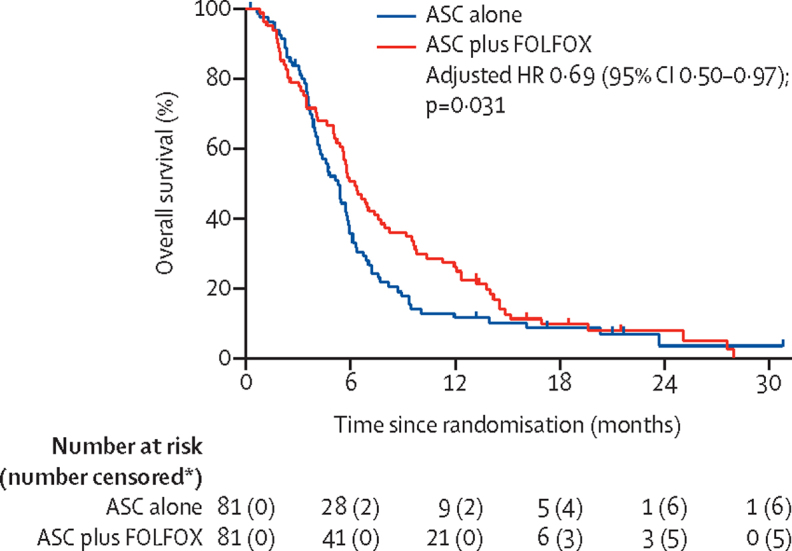
Overall survival The HR is adjusted for the three stratification factors (platinum sensitivity, serum albumin concentration, and disease stage). ASC=active symptom control. FOLFOX=folinic acid, fluorouracil, and oxaliplatin. HR=hazard ratio. *Numbers are cumulative.

By data cutoff, 150 patients had died: 74 in the ASC alone group and 76 in the ASC plus FOLFOX group. Median overall survival was 6·2 months (95% CI 5·4–7·6) in the ASC plus FOLFOX group versus 5·3 months (4·1–5·8) in the ASC alone group (adjusted HR 0·69 [95% CI 0·50–0·97], p=0·031; figure 2). The overall survival rate in the ASC alone group was 35·5% (95% CI 25·2–46·0) at 6 months and 11·4% (5·6–19·5) at 12 months, compared with 50·6% (39·3–60·9) at 6 months and 25·9% (17·0–35·8) at 12 months in the ASC plus FOLFOX group. No evidence was identified against the key proportional hazards assumption (proportional hazards assumption test p=0·65).

The other factors included in the multivariable analyses for overall survival were platinum sensitivity (adjusted HR 0·71, 0·49–1·00; p=0·050), high albumin at baseline (0·54, 0·37–0·78; p=0·0010), and metastatic disease (1·33, 0·85–2·09; p=0·21).

At the time of data cleaning and quality-control checks, stratification errors were identified with incorrect baseline information provided by study sites at the time of randomisation. A post-hoc sensitivity analysis for overall survival using the categories provided by local investigators at the time of randomisation confirmed the beneficial effect of FOLFOX plus ASC versus ASC alone on overall survival (adjusted HR 0·68, 95% CI 0·49–0·95; p=0·022; appendix p 3).

78 (96%) of 81 patients assigned to ASC plus FOLFOX had disease progression or had died at the time of data analysis. Median progression-free survival was 4·0 months (95% CI 3·2–5·0; appendix p 4). The progression-free survival rate was 66·7% (95% CI 55·3–75·8) at 3 months, 32·1% (22·3–42·3) at 6 months, and 8·6% (3·8–16·0) at 12 months. Objective response was observed in four (5%) of 81 patients in the ASC plus FOLFOX group: one (1%) patient had a complete response and three (4%) patients had a partial response. One (1%) patient did not have measurable disease, but was included in the analysis for evaluation of progression or stabilisation. Disease control was observed in 27 (33%) of 81 patients, including 23 (28%) patients with stable disease. The remaining 53 patients were non-responders: 30 (37%) patients had disease progression and 23 (28%) patients died. Three patients were still free from disease progression and alive at time of last follow-up, with individual progression-free survival ranging between 13·3 and 18·4 months; in one of these patients, a maintained radiological complete response was observed.

**Figure 3:**
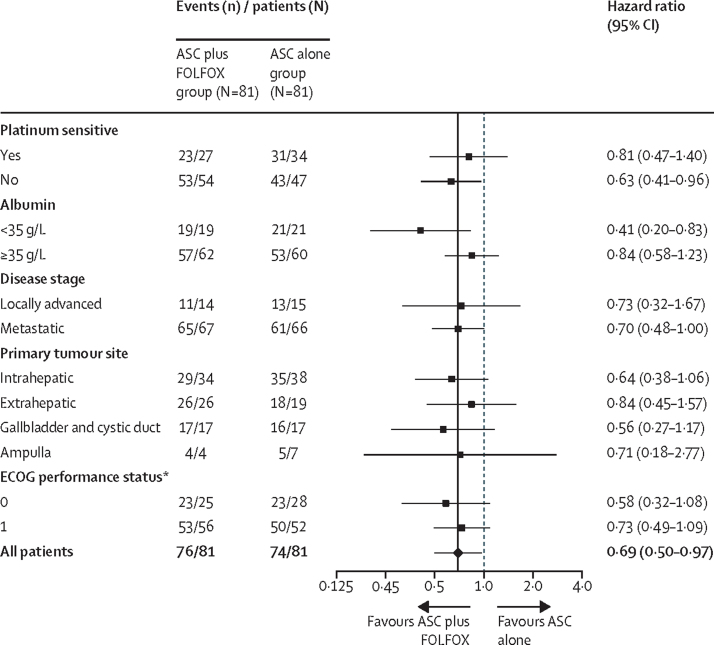
Subgroup analyses of overall survival Hazard ratios were adjusted by the three stratification factors (platinum sensitivity, serum albumin concentration, and disease stage), or the remaining two stratification factors where the factor of interest was itself a stratification factor. ASC=active symptom control. ECOG=Eastern Cooperative Oncology Group. FOLFOX=folinic acid, fluorouracil, and oxaliplatin. *ECOG performance status information was missing for one participant (death) in the ASC alone group.

Subgroup analyses of overall survival are shown in figure 3. Estimated median overall survival and progression-free survival for subgroups of interest are shown in the appendix (pp 5–9).

75 (93%) of 81 patients in the ASC plus FOLFOX group received at least one cycle of FOLFOX, with six patients receiving no chemotherapy for various reasons (some not reported). The median number of FOLFOX cycles received was five (IQR 2–6), with a median interval between the first and last doses received of 70 days (41–111). 46 (61%) of 75 patients required at least one component of FOLFOX chemotherapy to be dose reduced or omitted on one or more cycles. 13 (16%) of 81 patients completed all 12 cycles of FOLFOX. The main reasons for early discontinuation included radiological disease progression (24 patients), clinical disease progression (13 patients), intolerable toxicity (ten patients), intercurrent illness (five patients), patient decision (five patients), investigator decision (five patients), or other unspecified reason (three patients).

**Figure 4:**
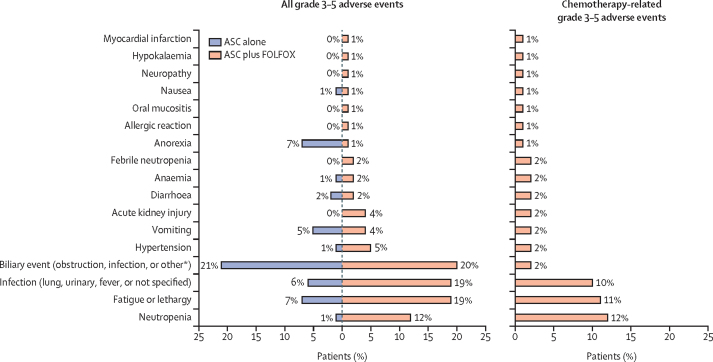
Grade 3–5 adverse events and chemotherapy-related toxicity Grade 3–5 adverse events reported at least in 1% of patients in the ASC plus FOLFOX group that were considered to be chemotherapy related are summarised, alongside the incidence in each study group regardless of causality. Percentages are calculated in the intention-to-treat population. ASC=active symptom control. FOLFOX=folinic acid, fluorouracil, and oxaliplatin. *Includes liver infection, increased bilirubin or alkaline phosphatase, and hepatitis.

Grade 3–5 adverse events were reported in 56 (69%) of 81 patients in the ASC plus FOLFOX group and 42 (52%) of 81 patients in the ASC alone group. Three chemotherapy-related deaths (one each due to infection, acute kidney injury, and febrile neutropenia) were reported in the ASC plus FOLFOX group. All other deaths reported in both groups were cancer related, with the exception of ten deaths associated with intercurrent illness (eight in the ASC plus FOLFOX group and two in the ASC-only group); cause of death was not reported for one patient in the ASC alone group. The most frequently reported grade 3–5 chemotherapy-related adverse events were neutropenia (ten [12%] patients), fatigue or lethargy (nine [11%] patients), and infection (eight [10%] patients; table 2; figure 4). Full details on adverse events are provided in the appendix (pp 10–11 for grade 3–5, p 12 for grade 1–2 adverse events regardless of causality, and p 13 for chemotherapy-related serious adverse events for the ASC plus FOLFOX group.

**Table 2:** Adverse events and chemotherapy-related toxicity

	**Grade 1–2**			**Grade 3**			**Grade 4**			**Grade 5**		
	All events (regardless of causality)		Chemotherapy-related events (ASC plus FOLFOX; n=81)	All events (regardless of causality)		Chemotherapy-related events (ASC plus FOLFOX; n=81)	All events (regardless of causality)		Chemotherapy-related events (ASC plus FOLFOX; n=81)	All events (regardless of causality)		Chemotherapy-related events (ASC plus FOLFOX; n=81)
	ASC alone (n=81)	ASC plus FOLFOX (n=81)		ASC alone (n=81)	ASC plus FOLFOX (n=81)		ASC alone (n=81)	ASC plus FOLFOX (n=81)		ASC alone (n=81)	ASC plus FOLFOX (n=81)	
Any*	35 (43%)	24 (30%)	37 (46%)	35 (43%)	39 (48%)	23 (28%)	3 (4%)	10 (12%)	5 (6%)	4 (5%)	7 (9%)	3 (4%)
Neuropathy	8 (10%)	55 (68%)	52 (64%)	0	1 (1%)	1 (1%)	0	0	0	0	0	0
Fatigue or lethargy	47 (58%)	58 (72%)	47 (58%)	6 (7%)	15 (19%)	9 (11%)	0	0	0	0	0	0
Nausea	32 (40%)	40 (49%)	30 (37%)	1 (1%)	1 (1%)	1 (1%)	0	0	0	0	0	0
Oral mucositis	4 (5%)	29 (36%)	28 (35%)	0	1 (1%)	1 (1%)	0	0	0	0	0	0
Anorexia	31 (38%)	47 (58%)	25 (31%)	6 (7%)	1 (1%)	1 (1%)	0	0	0	0	0	0
Diarrhoea	12 (15%)	27 (33%)	22 (27%)	2 (2%)	2 (2%)	2 (2%)	0	0	0	0	0	0
Thrombocytopenia	1 (1%)	18 (22%)	18 (22%)	0	0	0	0	0	0	0	0	0
Dysgeusia	11 (14%)	23 (28%)	16 (20%)	1 (1%)	0	0	0	0	0	0	0	0
Vomiting	16 (20%)	20 (25%)	14 (17%)	4 (5%)	3 (4%)	2 (2%)	0	0	0	0	0	0
Constipation	28 (35%)	35 (43%)	13 (16%)	1 (1%)	2 (2%)	0	0	0	0	0	0	0
Neutropenia	0	13 (16%)	12 (15%)	1 (1%)	8 (10%)	8 (10%)	0	2 (2%)	2 (2%)	0	0	0
Infection†	17 (21%)	19 (23%)	10 (12%)	3 (4%)	12 (15%)	6 (7%)	0	2 (2%)	1 (1%)	2 (2%)	1 (1%)	1 (1%)
Anaemia	5 (6%)	10 (12%)	9 (11%)	1 (1%)	2 (2%)	2 (2%)	0	0	0	0	0	0
Dry mouth	11 (14%)	20 (25%)	9 (11%)	0	1 (1%)	0	0	0	0	0	0	0
Pain	50 (62%)	42 (52%)	6 (7%)	5 (6%)	8 (10%)	0	1 (1%)	0	0	0	0	0
Tinnitus	2 (2%)	8 (10%)	5 (6%)	0	0	0	0	0	0	0	0	0
Myalgia	5 (6%)	10 (12%)	4 (5%)	1 (1%)	0	0	0	0	0	0	0	0
Oedema	9 (11%)	17 (21%)	4 (5%)	1 (1%)	0	0	0	1 (1%)	0	0	0	0
Dyspnoea	6 (7%)	13 (16%)	3 (4%)	1 (1%)	1 (1%)	0	0	0	0	0	0	0
Muscle weakness	9 (11%)	6 (7%)	3 (4%)	0	0	0	0	0	0	0	0	0
Thromboembolic event	2 (2%)	3 (4%)	3 (4%)	4 (5%)	0	0	0	0	0	0	0	0
Cough	4 (5%)	11 (14%)	2 (2%)	0	0	0	0	0	0	0	0	0
Dyspepsia	10 (12%)	8 (10%)	2 (2%)	0	0	0	0	0	0	0	0	0
Weight loss	9 (11%)	8 (10%)	2 (2%)	0	0	0	0	0	0	0	0	0
Abdominal distension	7 (9%)	3 (4%)	1 (1%)	0	2 (2%)	0	0	0	0	0	0	0
Biliary event‡	2 (2%)	2 (2%)	1 (1%)	13 (16%)	13 (16%)	2 (2%)	2 (2%)	2 (2%)	0	2 (2%)	1 (1%)	0
Catheter-related infection	0	2 (2%)	1 (1%)	0	2 (2%)	0	0	0	0	0	0	0
Erythema	1 (1%)	2 (2%)	1 (1%)	1 (1%)	0	0	0	0	0	0	0	0
Hypertension	4 (5%)	10 (12%)	1 (1%)	1 (1%)	4 (5%)	2 (2%)	0	0	0	0	0	0
Hypophosphatemia	0	2 (2%)	1 (1%)	1 (1%)	0	0	0	1 (1%)	0	0	0	0
Hypotension	1 (1%)	1 (1%)	1 (1%)	0	1 (1%)	0	0	0	0	0	0	0
Acute kidney injury	2 (2%)	0	0	0	1 (1%)	1 (1%)	0	0	0	0	2 (2%)	1 (1%)
Allergic reaction	0	0	0	0	1 (1%)	1 (1%)	0	0	0	0	0	0
Ascites	2 (2%)	10 (12%)	0	2 (2%)	2 (2%)	0	0	0	0	0	0	0
Cerebrovascular ischaemia	0	0	0	0	0	0	0	1 (1%)	0	0	0	0
Dehydration	0	0	0	1 (1%)	0	0	0	0	0	0	0	0
Diabetic ketoacidosis	0	0	0	0	0	0	0	0	0	0	1 (1%)	0
Fall	0	0	0	0	1 (1%)	0	0	0	0	0	0	0
Febrile neutropenia	0	0	0	0	0	0	0	1 (1%)	1 (1%)	0	1 (1%)	1 (1%)
Fracture (non-pathological)	0	0	0	0	1 (1%)	0	0	0	0	0	0	0
Gastric outlet obstruction	0	0	0	1 (1%)	0	0	0	0	0	0	0	0
Gastrointestinal bleeding	3 (4%)	1 (1%)	0	2 (2%)	1 (1%)	0	0	0	0	0	0	0
Generalised muscle weakness	1 (1%)	0	0	0	1 (1%)	0	0	0	0	0	0	0
Hallucinations	1 (1%)	0	0	0	1 (1%)	0	0	0	0	0	0	0
Hip fracture	0	0	0	1 (1%)	0	0	0	0	0	0	0	0
Hypercalcemia	1 (1%)	1 (1%)	0	1 (1%)	1 (1%)	0	0	1 (1%)	0	0	0	0
Hyperglycaemia	2 (2%)	2 (2%)	0	0	2 (2%)	0	0	0	0	0	0	0
Hypoalbuminemia	1 (1%)	2 (2%)	0	0	1 (1%)	0	0	0	0	0	0	0
Hypokalaemia	0	0	0	0	1 (1%)	1 (1%)	0	0	0	0	0	0
Hyponatremia	2 (2%)	2 (2%)	0	1 (1%)	0	0	0	0	0	0	0	0
Hypoxia	0	0	0	0	1 (1%)	0	0	0	0	0	0	0
Insomnia	6 (7%)	4 (5%)	0	0	1 (1%)	0	0	0	0	0	0	0
Leg oedema	3 (4%)	3 (4%)	0	1 (1%)	0	0	0	0	0	0	0	0
Liver failure	0	0	0	1 (1%)	0	0	0	0	0	0	0	0
Myocardial infarction	0	0	0	0	0	0	0	1 (1%)	1 (1%)	0	0	0
Pleural effusion	0	0	0	0	2 (2%)	0	0	0	0	0	0	0
Rhabdomyolisis	0	0	0	0	0	0	0	0	0	0	1 (1%)	0

Adverse events are listed in order of frequency, with the most frequent chemotherapy-related pooled grade 1/2 adverse events listed first. All grade 1–2 adverse events occurring in at least 10% of patients and all grade 3, 4, and 5 events are reported. Full details are available in the appendix (p 10). All percentages are calculated by intention to treat. ASC=active symptom control. FOLFOX=folinic acid, fluorouracil, and oxaliplatin.

* Refers to highest grade overall within-subject adverse events.

† Lung, urinary, fever, or not specified, excluding liver or biliary.

‡ Includes liver infection, increased bilirubin or alkaline phosphatase, and hepatitis.

Eight (10%) of 81 patients assigned to ASC only received off-protocol chemotherapy. 21 (13%) of 162 patients received some form of subsequent systemic anticancer therapy after the trial: five (3%) in the form of phase 1 trials and 16 (10%) in the form of chemotherapy agents (appendix p 14).

## Discussion

The results of the ABC-06 study show that FOLFOX chemotherapy can improve overall survival in patients with good performance status with advanced biliary tract cancer who have been previously treated with cisplatin and gemcitabine. To our knowledge, this trial is the first prospective randomised phase 3 study to evaluate the additional role of chemotherapy with ASC compared with ASC alone in these patients. Although some clinicians have already used FOLFOX in this setting, they were doing so without knowledge of the magnitude of benefit.^6^, ^7^

In the ABC-06 trial, patients who received ASC (which was the evidence-based approach in this setting before this study) showed a longer survival than expected. This has also been reported in two contemporary randomised clinical trials, exploring the role of regorafenib (multi-tyrosine kinase inhibitor)^29^ and ivosidenib (IDH1 inhibitor)^17^ in the second-line setting for patients with advanced biliary tract cancer. Both trials contained a placebo group, in which the management was similar to the ASC approach described here. The reported median overall survival for the control groups in those trials was 5·1 months^29^ and 6 months^17^ (when adjusted for crossover), respectively. The longer than expected overall survival in the ASC or placebo groups might be related to patient selection; patients who are fit enough to enter a second-line clinical trial are more likely to have a more favourable natural history. Nevertheless, this study supports the position that even when anticancer strategies are not being pursued, ASC (a proactive supportive care) rather than reactive management of symptoms is of benefit and might improve survival.^30^

The benefit achieved with the addition of FOLFOX chemotherapy in terms of median overall survival might seem marginal (lessened by a better than expected overall survival in the ASC alone group); this finding has also been described in other studies in this setting and might reflect the rapid deterioration of some patients before treatment has time to have a biological effect.^31^ However, the overall reduction in risk of death and positive impact on 6-month and 12-month overall survival rates are clinically meaningful. The toxicity profile of FOLFOX was tolerable and in line with that described with FOLFOX in many other malignancies.

Given the randomised design of this study, the effect of adding chemotherapy can be seen clearly. Few other clinical trials (none of them phase 3) exploring the role of cytotoxic agents (eg, etoposide toniribate [EDO-S7.1],^32^ FOLFIRINOX [fluorouracil, irinotecan, and oxaliplatin],^33^ and other small molecules [eg, regorafenib]^29^) have been reported in this setting. Although the results from these studies are of interest, interstudy comparison is not possible because of differences in baseline characteristics, such as line of therapy or primary tumour site (appendix pp 15–16), some of which are known to affect prognosis.^34^

Targeted therapies (ie, FGFR2 and IDH1 inhibitors) have a potential role in the second-line setting for advanced biliary tract cancer. Patients harbouring such alterations would be suitable for precision medicine strategies and their outcome might be difficult to compare with other advanced biliary tract cancers without such alterations.^18^ The natural history of these subgroups (including within ABC-06) is not yet fully understood and the timing of targeted therapies with respect to standard systemic chemotherapy (including FOLFOX in second line) is an area for future clinical and translational research.

A contentious issue with the ABC-06 clinical trial was the fact that it employed a platinum agent (oxaliplatin) following disease progression on another platinum (cisplatin).^35^ Platinum drugs are one of the active agents in advanced biliary tract cancer, together with gemcitabine and fluoropyrimidines.^13^ The third-generation platinum analogue oxaliplatin has activity in several gastrointestinal tumours and shows synergistic activity with a favourable toxicity profile in combination with fluorouracil that differs from the synergies proposed for cisplatin and gemcitabine.^36^ Additionally, non-cross resistance between oxaliplatin and cisplatin has been confirmed, although remains controversial.^37^, ^38^ The ABC-06 study showed how the overall survival and progression-free survival benefit was independent of whether patients were classed as being platinum sensitive or platinum refractory or resistant. In fact, subgroups with suspected poorer prognosis seemed to benefit most from FOLFOX. A potential explanation is that those patients with more aggressive tumours are the ones deriving more benefit from an aggressive antiproliferative therapy such as second-line chemotherapy. However, no strong conclusions can be derived in view of insufficient statistical power for subgroup analyses. These observations highlight the importance of a better understanding of the mechanisms behind response to platinum-based chemotherapy, such as pathways involved in DNA damage repair,^39^ among others.

Our subgroup analysis also showed a weaker overall survival benefit from treatment with FOLFOX in the extrahepatic cholangiocarcinoma subgroup; however, this observation should be viewed with caution owing to insufficient statistical power. We would still encourage the use of FOLFOX for this patient population following progression on cisplatin and gemcitabine.

Limitations of this study include its conduct in a single country. However, the magnitude of benefit previously observed with first-line cisplatin and gemcitabine chemotherapy was consistent across geographical regions,^40^ and this can reasonably also be expected in the second-line setting. Additionally, current predominant practice in the UK during first-line chemotherapy is to interrupt treatment at the completion of 6 months of therapy (as per the ABC-02 trial protocol), whereas other countries might continue treatment until disease progression. The effect of this practice on the efficacy of second-line therapy is not yet well understood. Furthermore, countries have varying access to molecular profiling (especially during the time this study was recruiting). Routine molecular profiling was not available for patients participating in ABC-06 because it was not protocol driven nor standard of care at the time of study conduct; thus, the impact on patients with specific molecular aberrations is not known.

We cannot exclude that ASC in the chemotherapy group was more meticulous than in the ASC alone group, but we considered fortnightly visits for patients not receiving chemotherapy too onerous; moreover, this is unlikely to have had a substantial effect on overall survival. Primary disease site, previous surgery, and ECOG performance status were not included as stratification factors since the magnitude of benefit from chemotherapy was expected to be consistent across these subgroups; since most baseline characteristics were well balanced between study groups, it is unlikely that having stratified for these factors would have affected the study outcome. It is difficult to determine whether the differences in pre-treatment CA19.9 between study groups is of any relevance, and this should be explored in the future. Platinum-refractory and platinum-resistant patients were analysed together; however, raw data would allow for further subsequent analysis (possibly via meta-analyses with other studies). Additionally, although platinum sensitivity was predefined for ABC-06, this definition has not yet been validated in biliary tract cancer and should be further interrogated in future studies. The insufficient power for subgroup analyses, which challenges some interpretations, is an acknowledged limitation, especially in such a heterogeneous disease. Routine radiological follow-up of patients assigned to the ASC only group was not pursued (because disease was likely to be steadily progressive), which limits the understanding and interpretation of progression-free survival and radiological response data, which are provided for the chemotherapy group only. Furthermore, the 3-monthly imaging, the absence of central radiology review, and the open-label design might have affected progression-free survival results and interpretation; however, the primary endpoint was unlikely to be affected by this. Subsequent lines of treatment were administered as per clinician discretion and could have affected overall survival; however, the proportion of patients receiving these was well balanced between the study groups and unlikely to affect the primary endpoint.

The ABC-06 clinical trial is, to our knowledge, the first randomised phase 3 clinical trial exploring the role of second-line chemotherapy in advanced biliary tract cancer. It shows a benefit from FOLFOX in terms of overall survival, with a meaningful increase in survival rates at 6 and 12 months. FOLFOX should therefore be considered the standard chemotherapy treatment after progression following cisplatin and gemcitabine and should be regarded as the reference regimen in future clinical trials in this setting.

## Data sharing

Data from the ABC-06 clinical trial can be made available to other researchers in the field upon request and approval by the trial management committee and subject to appropriate data transfer agreements. Requests should be directed to the corresponding author.

## Declaration of interests

AL has received travel and educational support from Ipsen, Pfizer, Bayer, AAA, SirtEx, Novartis, Mylan, and Delcath; speaker honoraria from Merck, Pfizer, Ipsen, and Incyte; advisory honoraria from EISAI, Nutricia Ipsen, QED, and Roche, outside of the submitted work. AL is also a member of the Knowledge Network and NETConnect Initiatives funded by Ipsen. DHP declares research grants from AstraZeneca, Bayer, Bristol-Myers Squibb, Nucana, and Sirtex; and consulting or advisory roles for AstraZeneca, Bayer, Bristol-Myers Squibb, Eisai, Merck Sharp & Dohme, Servier, and Roche, outside of the submitted work. HSW has participated in advisory boards for Incyte, Bayer, Roche/Genentech/Foundation Medicine, SIRTEX Medical, Celgene, and Zymeworks; has been a NICE expert for Bayer (uncompensated); and reports consultancies for ONCOSIL; speaker-related honoraria and travel support from BTG/Biocompatibles, Merck KGaA, and BMS; participation in trials steering committee for Pfizer and Zymeworks; creation of educational materials for AstraZeneca; and receipt of research funding from Sirtex, outside of the submitted work. PJR declares research grants from Sanofi and Bayer; consulting or advisory roles for Bayer, Bristol-Myers Squibb, Eisai, Sirtex, and Roche; speaker fees from Amgen, Roche, and Servier; and travel grants from Bayer, Roche, and Servier, outside of the submitted work. YTM declares speaker honoraria and advisory roles for Bayer, Eisai, and Roche, outside of the submitted work. JW declares research grants from AstraZeneca and Sanofi-Genzyme and consulting or advisory roles for Lilly, AstraZeneca, Sanofi Genyme, Eisai, AAA, Roche, Novartis, and Ipsen, outside of the submitted work. AM declares research grants from Pfizer, Bristol-Myers Squibb, and Bayer; speaker fees from Bristol-Myers Squibb, Bayer Ipsen, EUSA Pharma, Daiichi Sankyo, and Pfizer; and advisory roles for Bayer, Bristol-Myers Squibb, Leo, Pfizer, Merck, and Ipsen, outside of the submitted work. JSW has received educational support for travel and conference attendance from Mylan and Ipsen, and speaker honoraria from Mylan, outside of the submitted work. JR declares research grants, speaker fees, and advisory roles for Ipsen, Novartis, and AAA, outside of the submitted work. JAB declares consulting or advisory roles for Merck Serono, Servier, Roche, Bayer, AstraZeneca, Incyte, and Basilea; and travel support from MSD Oncology, Merck Serono, Servier, and Bristol-Myers Squibb, outside of the submitted work. JWV declares consulting or advisory roles for Agios, AstraZeneca, Delcath Systems, Keocyt, Genoscience Pharma, Incyte, Ipsen, Merck, Mundipharma EDO, Novartis, PCI Biotech, Pfizer, Pieris Pharmaceuticals, QED, and Wren Laboratories; speakers' bureau for Imaging Equipment, Ipsen, Novartis, and Nucana; and travel grants from Celgene and Nucana, outside of the submitted work. AAr, SF, RG, KP, AAn, TI, CH, SB, WDR, and LMD declare no competing interests.
