# Supplementary material for: Second-line FOLFOX chemotherapy versus active symptom control for advanced biliary tract cancer (ABC-06): a phase 3, open-label, randomised, controlled trial
Source: Lancet Oncol. 2021 May;22(5):690–701. doi: 10.1016/S1470-2045(21)00027-9 (PMC8082275; doi:10.1016/S1470-2045(21)00027-9)
Supplement: Supplementary appendix [file mmc1.pdf]

THE LANCET
Oncology

Supplementary appendix

This appendix formed part of the original submission and has been peer reviewed. We post it as supplied by the authors.

Supplement to: Lamarca D, Palmer DH, Wasan HS, et al. Second-line FOLFOX chemotherapy versus active symptom control for advanced biliary tract cancer (ABC-06): a phase 3, open-label, randomised, controlled trial . *Lancet Oncol* 2021; published online March 30. http://dx.doi.org/10.1016/S1470-2045(21)00027-9.

Appendix

Section A: Summary of Study Amendments 2

Section B: Sensitivity analysis (Overall survival) 3

Section C: Progression-free survival from second-line treatment in patients with advanced biliary tract cancer 4

Section D: Median overall survival and progression-free survival by baseline characteristics subgroups of interest 5

Section E: Overall survival and Progression-free survival subgroup analysis by platinum sensitivity 6

Section F: Patient outcome by primary tumour site. 9

Section G: Full details on reported AEs 10

Section H: Subsequent lines of systemic therapy 14

Section I: Summary of population characteristics and patient outcome for ABC-06 and other recent studies exploring the role of second –line therapy in advanced biliary cancer. 15

Section J: List of recruiting sites and principal investigators 17

Section K: Study protocol 18

Section A: Summary of Study Amendments

Amendment Reference	Date Amendment Approved	Amendment Details
Initial study approval	23 Oct 2013	Protocol v1.0 dated 10th September 2013
Substantial Amendment 1	02 Dec 2013	Protocol v2.0 dated 18th Oct 2013<br>• Update to SmPC for Oxaliplatin<br>• Addition of 2 research sites<br>• Minor update patient information sheet<br>• Minor changes to the wording of the patient diary
Substantial Amendment 2	23 May 2014	Protocol v3.0 dated 13th May 2014<br>• Addition of 2 sites<br>• Change the PI at the existing site<br>• Clarifications and updates to processes throughout protocol
Substantial Amendment 3	25 Sep 2014	Protocol v4.0 dated 18th Aug 2014<br>• Clarifications and updates to processes throughout protocol<br>• Update to Patient Information sheet
Substantial Amendment 4	23 Sep 2015	No protocol update<br>• Addition of 1 new site.<br>• Removal of 1 previous site.<br>• Change in PI at exiting site.
Substantial Amendment 5	13 Jan 2016	Protocol v5.0 dated 28th Oct 2015<br>• Clarifications and updates to processes throughout protocol.<br>• Changes made to the Patient Diary<br>• Changes made to the GP Letter
Substantial Amendment 6	19 Jul 2016	No protocol update<br>• Changes to PI's at 3 separate sites.
Substantial Amendment 7	09 Aug 2017	Protocol v6.0 dated 26th Jul 2017<br>• Extension to trial end date from 30/04/2017 to 30/11/2018<br>• Removal of 2 sites.<br>• Change of Principal Investigator 1 site<br>• Minor clarification in protocol
Substantial Amendment 8	15 May 2018	No protocol update.<br>• Updates to Reference Safety Information from the Fluorouracil SmPC and Oxaliplatin SmPC
Non-substantial Amendment 1	08 Jan 2019	Protocol v7.0 dated 03rd Dec 2018 (final version).<br>• Administrative changes throughout protocol due to *MAHSC-CTU* becoming 'Manchester Clinical Trial Unit'

Section B: Sensitivity analysis (Overall survival)

Sensitivity analysis for the overall survival (primary end-point) performing the analysis employing the categories provided by local investigators at time of randomisation was performed.

Factor	Reported at time of randomisation	Category following quality control	n
**Platinum sensitive**	No	Yes	21
	Yes	No	4
**Albumin ≥35 g/L**	No	Yes	2
	Yes	No	3
**Metastatic**	No	Yes	7
	Yes	No	17

Sensitivity analysis (primary end-point by stratification factor provided at randomisation)	Adjusted Hazard Ratio (Multivariate Cox Regression)	
	HR (95% CI)	P-value
**Treatment with ASC + FOLFOX (vs ASC only)**	0.68 (95% CI 0.49-0.95)	0.02
**Platinum sensitivity (vs refractory/resistant)**	0.63 (95% CI 0.43-0.93)	0.02
**Albumin at baseline ≥35g/L (vs <35g/L)**	0.49 (95% CI 0.34-0.73)	<0.001
**Metastatic disease (vs locally advanced)**	1.37 (95% CI 0.79-2.37)	0.26

Section C: Progression-free survival from second-line treatment in patients with advanced biliary tract cancer

Figure shows Kaplan-Meier estimates of progression-free survival (chemotherapy arm).

PFS: progression-free survival, CI: confidence interval: ASC: active symptom control; FOLFOX: oxaliplatin and 5-fluorouracil chemotherapy.

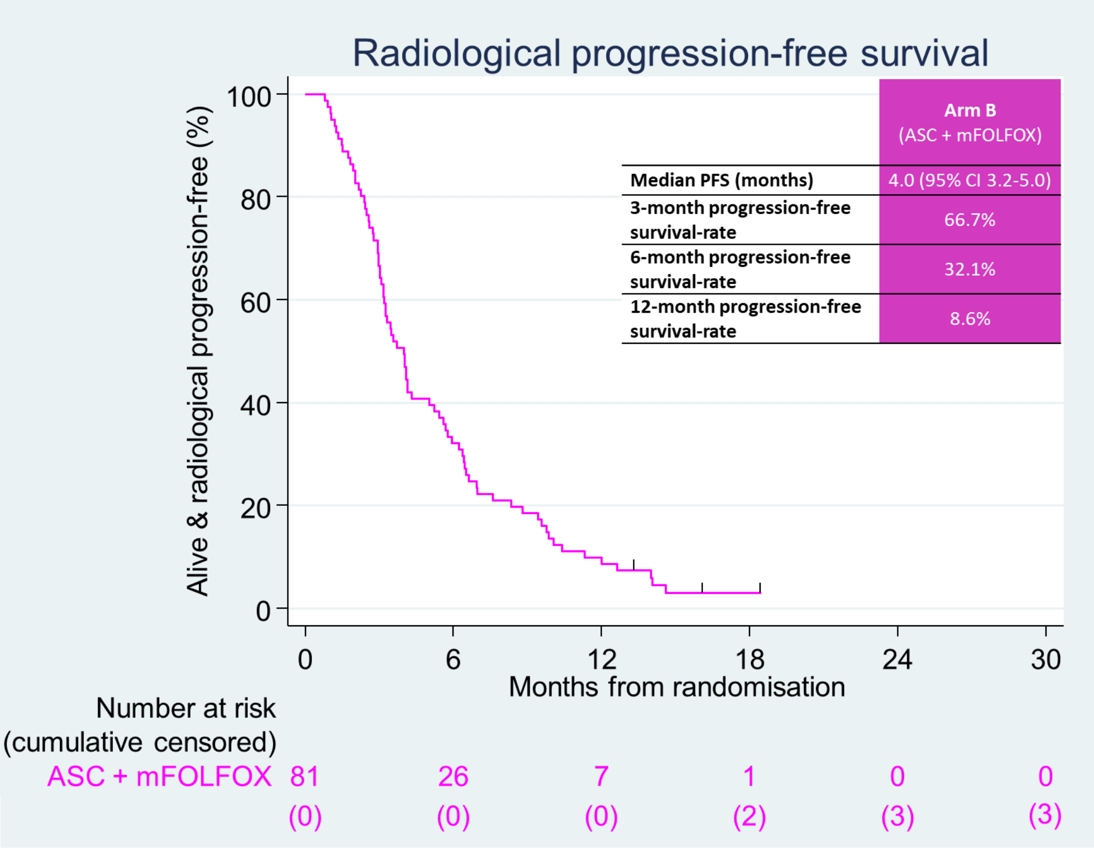

Section D: Median overall survival and progression-free survival by baseline characteristics subgroups of interest

OS: overall survival: PFS: progression-free survival; ASC: active symptom control; FOLFOX: 5-fluorouracil and oxaliplatin; ECOG: Eastern Cooperative Oncology Group; n: number of patients; CI: confidence interval; ne: not estimable.

*Stratification factors. **: ECOG-PS missing for 1 patient.

**Outcome explored**		**OS**				**PFS**	
**Trial arm**		**ASC alone**		**ASC + FOLFOX**		**ASC + FOLFOX**	
		**n**	**Median (months) (95% CI)**	**n**	**Median (months) (95% CI)**	**n**	**Median (months) (95% CI)**
**Platinum sensitivity***	Sensitive	34	5.7 (3.9-7.0)	27	6.6 (3.5-14.6)	27	4.1 (2.6-6.9)
	Resistant/refractory	47	4.8 (3.9-5.7)	54	5.9 (5.1-7.6)	54	3.6 (3.0-5.0)
**Albumin***	<35 g/L	21	3.7 (2.4-4.6)	19	5.8 (2.4-8.3)	19	4.0 (2.4-6.6)
	≥35 g/L	60	5.7 (4.8-6.4)	62	6.2 (5.4-9.2)	62	3.6 (3.1-5.4)
**Disease stage***	Locally advanced	15	5.8 (3.4-8.9)	14	6.6 (2.0-12.1)	14	4.1 (2-9.8)
	Metastatic	66	5.3 (4.2-5.9)	67	5.8 (5.1-7.6)	67	3.7 (3.0-5.2)
**Tumour site**	Intrahepatic	38	5.2 (3.7-5.8)	34	5.7 (4.1-7.4)	34	3.3 (2.5-5.2)
	Extrahepatic	19	5.4 (3.9-6.3)	26	6.2 (4.0-7.9)	26	4.0 (2.9-5.9)
	Gallbladder	17	4.6 (2.2-6.8)	17	5.1 (2.5-14.6)	17	3.9 (2.4-6.2)
	Ampulla	7	7.2 (3.6-ne)	4	10.4 (9.8-ne)	4	6.4 (4.1-ne)
**ECOG Performance Status****	0	28	6.1 (4.1-8.2)	25	8.3 (5.9-10.4)	25	4.0 (2.9-6.9)
	1	52	4.7 (3.8-5.7)	56	5.7 (4.1-6.4)	56	3.6 (3.2-4.3)

Section E: Overall survival and Progression-free survival subgroup analysis by platinum sensitivity

Panel A shows overall survival outcome by trial arm for patients with platinum sensitive disease; **panel B** shows overall survival outcome by trial arm for patients with platinum refractory/resistant disease. Survival curves limited to patients in the ASC + FOLFOX arm by platinum sensitivity for both OS (**Panel C**) and PFS (**Panel D**) are provided.

Patients with platinum-sensitive disease had a longer (borderline significant) OS compared to patients with platinum-resistance/refractory disease (HR 0.71 (95% CI 0.49-1.001); p-value 0.05; adjusted for trial arm, albumin and stage in the whole population); despite this, the effect of FOLFOX on OS (**Panels A-C, p 7-8)** was apparent in both platinum-sensitive (HR 0.81 (95% CI 0.47-1.4)) and refractory-resistant (HR 0.63 (95% CI 0.41-0.96)) patients; with no significant differences between groups in terms of PFS (**Panel D, p 8**).

* HRs adjusted for stratification factors (excluding platinum sensitivity) are provided.

Panel A: OS (platinum sensitive)

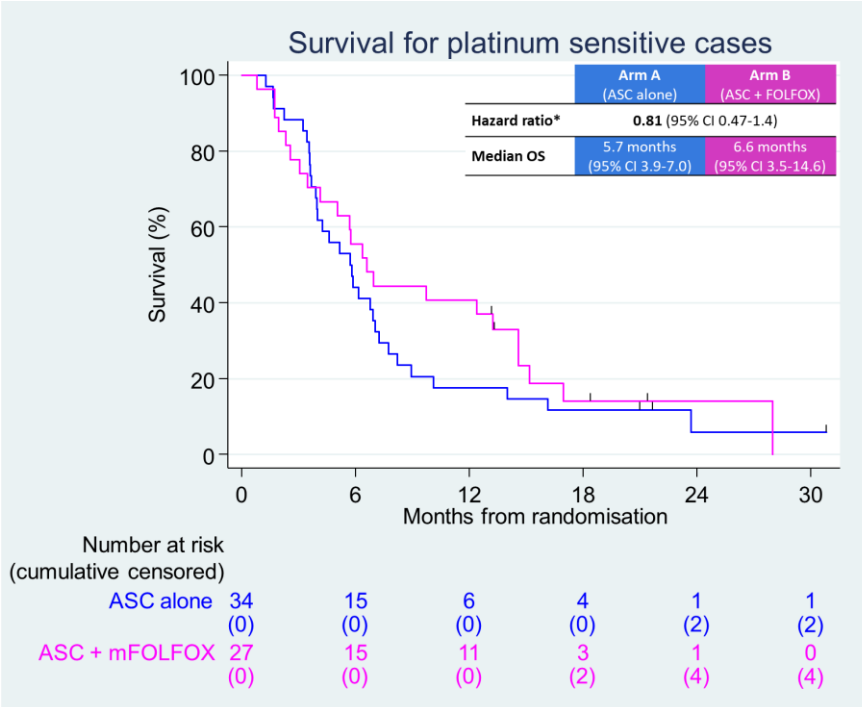

Panel B: OS (platinum resistant/refractory)

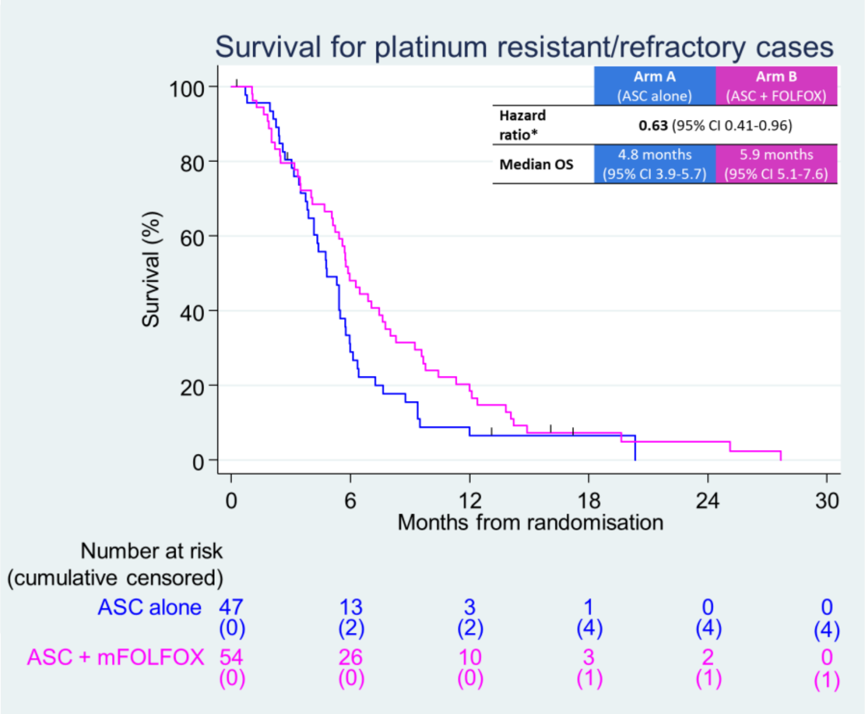

Panel C: OS in the ASC + FOLFOX arm by platinum sensitive vs. resistant/refractory

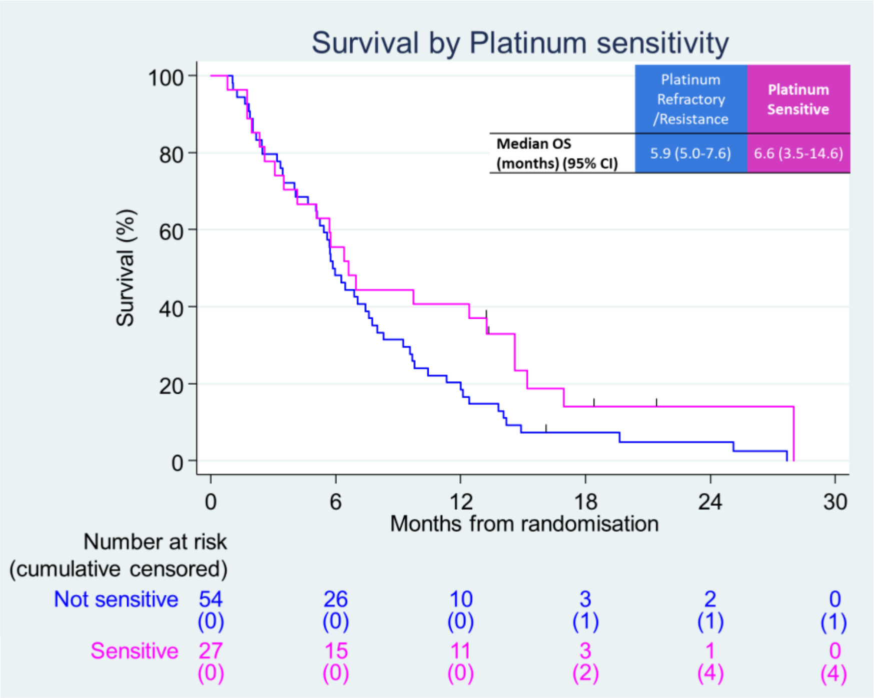

Panel D: PFS in the ASC + FOLFOX arm by platinum sensitive vs. resistant/refractory

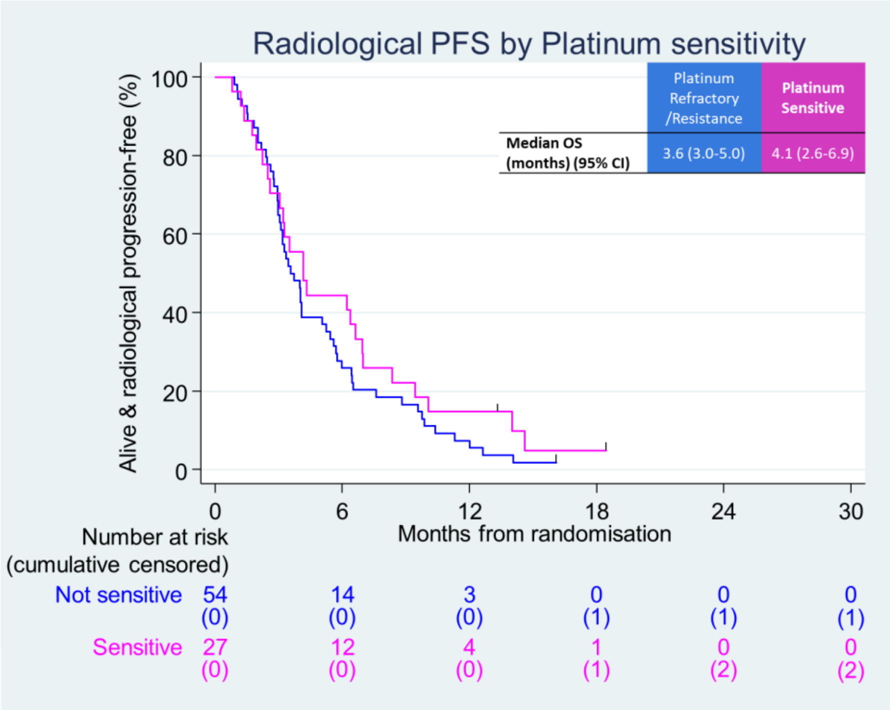

Section F: Patient outcome by primary tumour site.

The impact of FOLFOX on OS and PFS was consistent across the different primary tumour sites; although a more modest benefit for patients diagnosed with extrahepatic CCA was observed. PFS: progression free survival; iCCA: intrahepatic cholangiocarcinoma; eCCA: extrahepatic cholangiocarcinoma; OS: overall survival; CI: confidence interval: HR: hazard ratio; ASC: active symptom control; n: number of patients; ne: not estimable. * HRs adjusted for stratification factors are provided.

	All patients	iCCA	eCCA	Gallbladder cancer	Ampulla of Vater carcinoma
n (ASC-alone)	81	38	19	17	7
n (ASC+FOLFOX)	81	34	26	17	4
**Overall survival**					
Adjusted* HR (95% CI) OS	0.69 (0.50-0.97)	0.64 (0.38-1.06)	0.84 (0.45-1.57)	0.56 (0.27-1.17)	0.71 (0.18-2.77)
Median OS months (ASC-alone); months (95% CI)	5.3 (95% CI 4.1-5.8)	5.2 (3.7-5.8)	5.4 (3.9-6.4)	4.6 (2.2-6.8)	7.2 (3.6-ne)
Median OS months (ASC+FOLFOX); months (95% CI)	6.2 (95% CI 5.4-7.6)	5.7 (4.1-7.4)	6.2 (4.0-7.9)	5.1 (2.5-14.6)	10.4 (9.8-ne)
6m OS rate (ASC-alone) (%)	35.5%	30.8%	36.8%	35.3%	57.1%
6m OS rate (ASC+FOLFOX) (%)	50.6%	44.1%	53.9%	47.1%	100%
12m OS rate (ASC-alone) (%)	11.4%	11.2%	10.5%	5.9%	28.6%
12m OS rate (ASC+FOLFOX) (%)	25.9%	26.5%	15.4%	35.3%	50.0%
**Progression-free survival**					
Median PFS (ASC+FOLFOX); months (95% CI)	4.0 months (3.2-5.0)	3.3 months (2.5-5.2)	4.0 months (2.9-5.9)	4.0 months (2.4-6.2)	6.4 months (4.1-ne)
3m PFS rate (ASC+FOLFOX) (%)	66.7%	64.7%	65.4%	64.7%	100%
6m PFS rate (ASC+FOLFOX) (%)	32.1%	29.4%	30.8%	29.4%	75%
12m PFS rate (ASC+FOLFOX) (%)	8.6%	11.8%	3.9%	11.8%	0%
Complete response rate (ASC+FOLFOX); n (%)	1 (1%)	1 (3%)	0	0	0
Partial response rate (ASC+FOLFOX); n (%)	3 (4%)	0	0	3 (18%)	0
Overall Response rate (ASC+FOLFOX); n (%)	4 (5%)	1 (3%)	0	3 (18%)	0
Stable Disease rate (ASC+FOLFOX); n (%)	23 (28%)	7 (21%)	9 (35%)	5 (29%)	2 (50%)
Disease Control rate (ASC+FOLFOX); n (%)	27 (33%)	8 (24%)	9 (35%)	8 (47%)	2 (50%)

Section G: Full details on reported AEs

AE: adverse event; ASC: active symptom control; FOLFOX: 5-fluorouracil and oxaliplatin; pts: patients. All percentages are calculated using the total number of patient randomised to each arm as denominator.

@Refers to highest grade over all within subject AEs

*lung/urinary/fever/not specified, excluding liver/biliary; **includes: liver infection, increased bilirubin/alkaline phosphatase and hepatitis

TABLE G-1: Grade 3/4/5 AEs (all): in order of frequency, most frequent chemo-related grade 3/4/5 AEs are provided on the top of the table.

	Grade 3 AEs (n (%))			Grade 4 AEs (n (%))			Grade 5 AEs (n (%))			Grade 3/4/5 AEs (n (%))		
	All (regardless of causality)		Chemotherapy-related	All (regardless of causality)		Chemotherapy-related	All (regardless of causality)		Chemotherapy-related	All (regardless of causality)		Chemotherapy-related
	Arm A (ASC alone) n=81 pts	Arm B (ASC + FOLFOX) n=81 pts	Arm B (ASC + FOLFOX) n=81 pts	Arm A (ASC alone) n=81 pts	Arm B (ASC + FOLFOX) n=81 pts	Arm B (ASC + FOLFOX) n=81 pts	Arm A (ASC alone) n=81 pts	Arm B (ASC + FOLFOX) n=81 pts	Arm B (ASC + FOLFOX) n=81 pts	Arm A (ASC alone) n=81 pts	Arm B (ASC + FOLFOX) n=81 pts	Arm B (ASC + FOLFOX) n=81 pts
**Any@**	35 (43%)	39 (48%)	23 (28%)	3 (4%)	10 (12%)	5 (6%)	4 (5%)	7 (9%)	3 (4%)	42 (52%)	56 (69%)	31 (38%)
**Neutropenia**	1 (1%)	8 (10%)	8 (10%)	0 (0%)	2 (2%)	2 (2%)	0 (0%)	0 (0%)	0 (0%)	1 (1%)	10 (12%)	10 (12%)
**Fatigue/Lethargy**	6 (7%)	15 (19%)	9 (11%)	0 (0%)	0 (0%)	0 (0%)	0 (0%)	0 (0%)	0 (0%)	6 (7%)	15 (19%)	9 (11%)
**Infection***	3 (4%)	12 (15%)	6 (7%)	0 (0%)	2 (2%)	1 (1%)	2 (2%)	1 (1%)	1 (1%)	5 (6%)	15 (19%)	8 (10%)
**Biliary event****	13 (16%)	13 (16%)	2 (2%)	2 (2%)	2 (2%)	0 (0%)	2 (2%)	1 (1%)	0 (0%)	17 (21%)	16 (20%)	2 (2%)
**Hypertension**	1 (1%)	4 (5%)	2 (2%)	0 (0%)	0 (0%)	0 (0%)	0 (0%)	0 (0%)	0 (0%)	1 (1%)	4 (5%)	2 (2%)
**Vomiting**	4 (5%)	3 (4%)	2 (2%)	0 (0%)	0 (0%)	0 (0%)	0 (0%)	0 (0%)	0 (0%)	4 (5%)	3 (4%)	2 (2%)
**Acute kidney injury**	0 (0%)	1 (1%)	1 (1%)	0 (0%)	0 (0%)	0 (0%)	0 (0%)	2 (2%)	1 (1%)	0 (0%)	3 (4%)	2 (2%)
**Diarrhoea**	2 (2%)	2 (2%)	2 (2%)	0 (0%)	0 (0%)	0 (0%)	0 (0%)	0 (0%)	0 (0%)	2 (2%)	2 (2%)	2 (2%)
**Anaemia**	1 (1%)	2 (2%)	2 (2%)	0 (0%)	0 (0%)	0 (0%)	0 (0%)	0 (0%)	0 (0%)	1 (1%)	2 (2%)	2 (2%)
**Febrile neutropenia**	0 (0%)	0 (0%)	0 (0%)	0 (0%)	1 (1%)	1 (1%)	0 (0%)	1 (1%)	1 (1%)	0 (0%)	2 (2%)	2 (2%)
**Anorexia**	6 (7%)	1 (1%)	1 (1%)	0 (0%)	0 (0%)	0 (0%)	0 (0%)	0 (0%)	0 (0%)	6 (7%)	1 (1%)	1 (1%)
**Allergic reaction**	0 (0%)	1 (1%)	1 (1%)	0 (0%)	0 (0%)	0 (0%)	0 (0%)	0 (0%)	0 (0%)	0 (0%)	1 (1%)	1 (1%)
**Oral mucositis**	0 (0%)	1 (1%)	1 (1%)	0 (0%)	0 (0%)	0 (0%)	0 (0%)	0 (0%)	0 (0%)	0 (0%)	1 (1%)	1 (1%)
**Nausea**	1 (1%)	1 (1%)	1 (1%)	0 (0%)	0 (0%)	0 (0%)	0 (0%)	0 (0%)	0 (0%)	1 (1%)	1 (1%)	1 (1%)
**Neuropathy**	0 (0%)	1 (1%)	1 (1%)	0 (0%)	0 (0%)	0 (0%)	0 (0%)	0 (0%)	0 (0%)	0 (0%)	1 (1%)	1 (1%)
**Hypokalemia**	0 (0%)	1 (1%)	1 (1%)	0 (0%)	0 (0%)	0 (0%)	0 (0%)	0 (0%)	0 (0%)	0 (0%)	1 (1%)	1 (1%)
**Myocardial infarction**	0 (0%)	0 (0%)	0 (0%)	0 (0%)	1 (1%)	1 (1%)	0 (0%)	0 (0%)	0 (0%)	0 (0%)	1 (1%)	1 (1%)
**Constipation**	1 (1%)	2 (2%)	0 (0%)	0 (0%)	0 (0%)	0 (0%)	0 (0%)	0 (0%)	0 (0%)	1 (1%)	2 (2%)	0 (0%)

**Dry mouth**	0 (0%)	1 (1%)	0 (0%)	0 (0%)	0 (0%)	0 (0%)	0 (0%)	0 (0%)	0 (0%)	0 (0%)	1 (1%)	0 (0%)
**Dysgeusia**	1 (1%)	0 (0%)	0 (0%)	0 (0%)	0 (0%)	0 (0%)	0 (0%)	0 (0%)	0 (0%)	1 (1%)	0 (0%)	0 (0%)
**Myalgia**	1 (1%)	0 (0%)	0 (0%)	0 (0%)	0 (0%)	0 (0%)	0 (0%)	0 (0%)	0 (0%)	1 (1%)	0 (0%)	0 (0%)
**Oedema**	1 (1%)	0 (0%)	0 (0%)	0 (0%)	1 (1%)	0 (0%)	0 (0%)	0 (0%)	0 (0%)	1 (1%)	1 (1%)	0 (0%)
**Abdominal distension**	0 (0%)	2 (2%)	0 (0%)	0 (0%)	0 (0%)	0 (0%)	0 (0%)	0 (0%)	0 (0%)	0 (0%)	2 (2%)	0 (0%)
**Ascites**	2 (2%)	2 (2%)	0 (0%)	0 (0%)	0 (0%)	0 (0%)	0 (0%)	0 (0%)	0 (0%)	2 (2%)	2 (2%)	0 (0%)
**Catheter related infection**	0 (0%)	2 (2%)	0 (0%)	0 (0%)	0 (0%)	0 (0%)	0 (0%)	0 (0%)	0 (0%)	0 (0%)	2 (2%)	0 (0%)
**Cerebrovascular inchaemia**	0 (0%)	0 (0%)	0 (0%)	0 (0%)	1 (1%)	0 (0%)	0 (0%)	0 (0%)	0 (0%)	0 (0%)	1 (1%)	0 (0%)
**Dehydration**	1 (1%)	0 (0%)	0 (0%)	0 (0%)	0 (0%)	0 (0%)	0 (0%)	0 (0%)	0 (0%)	1 (1%)	0 (0%)	0 (0%)
**Diabetic ketoacidosis**	0 (0%)	0 (0%)	0 (0%)	0 (0%)	0 (0%)	0 (0%)	0 (0%)	1 (1%)	0 (0%)	0 (0%)	1 (1%)	0 (0%)
**Dyspnea**	1 (1%)	1 (1%)	0 (0%)	0 (0%)	0 (0%)	0 (0%)	0 (0%)	0 (0%)	0 (0%)	1 (1%)	1 (1%)	0 (0%)
**Erythema**	1 (1%)	0 (0%)	0 (0%)	0 (0%)	0 (0%)	0 (0%)	0 (0%)	0 (0%)	0 (0%)	1 (1%)	0 (0%)	0 (0%)
**Fall**	0 (0%)	1 (1%)	0 (0%)	0 (0%)	0 (0%)	0 (0%)	0 (0%)	0 (0%)	0 (0%)	0 (0%)	1 (1%)	0 (0%)
**Fracture (non-pathological)**	0 (0%)	1 (1%)	0 (0%)	0 (0%)	0 (0%)	0 (0%)	0 (0%)	0 (0%)	0 (0%)	0 (0%)	1 (1%)	0 (0%)
**Gastric outlet obstruction**	1 (1%)	0 (0%)	0 (0%)	0 (0%)	0 (0%)	0 (0%)	0 (0%)	0 (0%)	0 (0%)	1 (1%)	0 (0%)	0 (0%)
**Gastrointestinal bleeding**	2 (2%)	1 (1%)	0 (0%)	0 (0%)	0 (0%)	0 (0%)	0 (0%)	0 (0%)	0 (0%)	2 (2%)	1 (1%)	0 (0%)
**Generalized muscle weakness**	0 (0%)	1 (1%)	0 (0%)	0 (0%)	0 (0%)	0 (0%)	0 (0%)	0 (0%)	0 (0%)	0 (0%)	1 (1%)	0 (0%)
**Hallucinations**	0 (0%)	1 (1%)	0 (0%)	0 (0%)	0 (0%)	0 (0%)	0 (0%)	0 (0%)	0 (0%)	0 (0%)	1 (1%)	0 (0%)
**Hip fracture**	1 (1%)	0 (0%)	0 (0%)	0 (0%)	0 (0%)	0 (0%)	0 (0%)	0 (0%)	0 (0%)	1 (1%)	0 (0%)	0 (0%)
**Hypercalcemia**	1 (1%)	1 (1%)	0 (0%)	0 (0%)	1 (1%)	0 (0%)	0 (0%)	0 (0%)	0 (0%)	1 (1%)	2 (2%)	0 (0%)
**Hyperglycemia**	0 (0%)	2 (2%)	0 (0%)	0 (0%)	0 (0%)	0 (0%)	0 (0%)	0 (0%)	0 (0%)	0 (0%)	2 (2%)	0 (0%)
**Hypoalbuminemia**	0 (0%)	1 (1%)	0 (0%)	0 (0%)	0 (0%)	0 (0%)	0 (0%)	0 (0%)	0 (0%)	0 (0%)	1 (1%)	0 (0%)
**Hyponatremia**	1 (1%)	0 (0%)	0 (0%)	0 (0%)	0 (0%)	0 (0%)	0 (0%)	0 (0%)	0 (0%)	1 (1%)	0 (0%)	0 (0%)
**Hypophosphatemia**	1 (1%)	0 (0%)	0 (0%)	0 (0%)	1 (1%)	0 (0%)	0 (0%)	0 (0%)	0 (0%)	1 (1%)	1 (1%)	0 (0%)
**Hypotension**	0 (0%)	1 (1%)	0 (0%)	0 (0%)	0 (0%)	0 (0%)	0 (0%)	0 (0%)	0 (0%)	0 (0%)	1 (1%)	0 (0%)
**Hypoxia**	0 (0%)	1 (1%)	0 (0%)	0 (0%)	0 (0%)	0 (0%)	0 (0%)	0 (0%)	0 (0%)	0 (0%)	1 (1%)	0 (0%)
**Insomnia**	0 (0%)	1 (1%)	0 (0%)	0 (0%)	0 (0%)	0 (0%)	0 (0%)	0 (0%)	0 (0%)	0 (0%)	1 (1%)	0 (0%)
**Leg oedema**	1 (1%)	0 (0%)	0 (0%)	0 (0%)	0 (0%)	0 (0%)	0 (0%)	0 (0%)	0 (0%)	1 (1%)	0 (0%)	0 (0%)
**Liver failure**	1 (1%)	0 (0%)	0 (0%)	0 (0%)	0 (0%)	0 (0%)	0 (0%)	0 (0%)	0 (0%)	1 (1%)	0 (0%)	0 (0%)
**Pain**	5 (6%)	8 (10%)	0 (0%)	1 (1%)	0 (0%)	0 (0%)	0 (0%)	0 (0%)	0 (0%)	6 (7%)	8 (10%)	0 (0%)
**Pleural effusion**	0 (0%)	2 (2%)	0 (0%)	0 (0%)	0 (0%)	0 (0%)	0 (0%)	0 (0%)	0 (0%)	0 (0%)	2 (2%)	0 (0%)
**Rhabdomyolisis**	0 (0%)	0 (0%)	0 (0%)	0 (0%)	0 (0%)	0 (0%)	0 (0%)	1 (1%)	0 (0%)	0 (0%)	1 (1%)	0 (0%)
**Thromboembolic event**	4 (5%)	0 (0%)	0 (0%)	0 (0%)	0 (0%)	0 (0%)	0 (0%)	0 (0%)	0 (0%)	4 (5%)	0 (0%)	0 (0%)

TABLE G-2: Grade 1/2 AEs (if frequency ≥10% in any arm, regardless of causality): AEs are provided in order of frequency, most frequent chemo-related grade 1/2 AEs are provided on the top of the table.

	Grade 1 AEs (n (%))			Grade 2 AEs (n (%))			Grade 1/2 AEs (n (%))		
	All (regardless of causality)		Chemotherapy-related	All (regardless of causality)		Chemotherapy-related	All (regardless of causality)		Chemotherapy-related
	Arm A (ASC alone) n=81 pts	Arm B (ASC + FOLFOX) n=81 pts	Arm B (ASC + FOLFOX) n=81 pts	Arm A (ASC alone) n=81 pts	Arm B (ASC + FOLFOX) n=81 pts	Arm B (ASC + FOLFOX) n=81 pts	Arm A (ASC alone) n=81 pts	Arm B (ASC + FOLFOX) n=81 pts	Arm B (ASC + FOLFOX) n=81 pts
**Any[@]**	10 (12%)	4 (5%)	7 (9%)	25 (31%)	20 (25%)	30 (37%)	35 (43%)	24 (30%)	37 (46%)
**Neuropathy**	8 (10%)	38 (47%)	35 (43%)	0 (0%)	17 (21%)	17 (21%)	8 (10%)	55 (68%)	52 (64%)
**Fatigue/Lethargy**	21 (26%)	23 (28%)	22 (27%)	26 (32%)	35 (43%)	25 (31%)	47 (58%)	58 (72%)	47 (58%)
**Nausea**	24 (30%)	25 (31%)	20 (25%)	8 (10%)	15 (19%)	10 (12%)	32 (40%)	40 (49%)	30 (37%)
**Oral mucositis**	4 (5%)	23 (28%)	22 (27%)	0 (0%)	6 (7%)	6 (7%)	4 (5%)	29 (36%)	28 (35%)
**Anorexia**	20 (25%)	28 (35%)	16 (20%)	11 (14%)	19 (23%)	9 (11%)	31 (38%)	47 (58%)	25 (31%)
**Diarrhoea**	10 (12%)	18 (22%)	16 (20%)	2 (2%)	9 (11%)	6 (7%)	12 (15%)	27 (33%)	22 (27%)
**Thrombocytopenia**	1 (1%)	8 (10%)	8 (10%)	0 (0%)	10 (12%)	10 (12%)	1 (1%)	18 (22%)	18 (22%)
**Dysgeusia**	8 (10%)	22 (27%)	15 (19%)	3 (4%)	1 (1%)	1 (1%)	11 (14%)	23 (28%)	16 (20%)
**Vomiting**	12 (15%)	14 (17%)	9 (11%)	4 (5%)	6 (7%)	5 (6%)	16 (20%)	20 (25%)	14 (17%)
**Constipation**	20 (25%)	23 (28%)	10 (12%)	8 (10%)	12 (15%)	3 (4%)	28 (35%)	35 (43%)	13 (16%)
**Neutropenia**	0 (0%)	8 (10%)	7 (9%)	0 (0%)	5 (6%)	5 (6%)	0 (0%)	13 (16%)	12 (15%)
**Infection***	11 (14%)	10 (12%)	6 (7%)	6 (7%)	9 (11%)	4 (5%)	17 (21%)	19 (23%)	10 (12%)
**Dry mouth**	6 (7%)	15 (19%)	6 (7%)	5 (6%)	5 (6%)	3 (4%)	11 (14%)	20 (25%)	9 (11%)
**Anaemia**	3 (4%)	4 (5%)	5 (6%)	2 (2%)	6 (7%)	4 (5%)	5 (6%)	10 (12%)	9 (11%)
**Pain**	22 (27%)	25 (31%)	4 (5%)	28 (35%)	17 (21%)	2 (2%)	50 (62%)	42 (52%)	6 (7%)
**Tinnitus**	2 (2%)	7 (9%)	4 (5%)	0 (0%)	1 (1%)	1 (1%)	2 (2%)	8 (10%)	5 (6%)
**Myalgia**	4 (5%)	7 (9%)	3 (4%)	1 (1%)	3 (4%)	1 (1%)	5 (6%)	10 (12%)	4 (5%)
**Oedema**	5 (6%)	11 (14%)	3 (4%)	4 (5%)	6 (7%)	1 (1%)	9 (11%)	17 (21%)	4 (5%)
**Muscle weakness**	7 (9%)	6 (7%)	3 (4%)	2 (2%)	0 (0%)	0 (0%)	9 (11%)	6 (7%)	3 (4%)
**Dyspepsia**	4 (5%)	7 (9%)	2 (2%)	6 (7%)	1 (1%)	0 (0%)	10 (12%)	8 (10%)	2 (2%)
**Cough**	3 (4%)	9 (11%)	1 (1%)	1 (1%)	2 (2%)	1 (1%)	4 (5%)	11 (14%)	2 (2%)
**Dyspnea**	5 (6%)	11 (14%)	2 (2%)	1 (1%)	2 (2%)	1 (1%)	6 (7%)	13 (16%)	3 (4%)
**Weight loss**	6 (7%)	7 (9%)	2 (2%)	3 (4%)	1 (1%)	0 (0%)	9 (11%)	8 (10%)	2 (2%)
**Hypertension**	1 (1%)	5 (6%)	1 (1%)	3 (4%)	5 (6%)	0 (0%)	4 (5%)	10 (12%)	1 (1%)
**Ascites**	1 (1%)	2 (2%)	0 (0%)	1 (1%)	8 (10%)	0 (0%)	2 (2%)	10 (12%)	0 (0%)

TABLE G-3: chemotherapy-related Severe AEs (SAEs) (all): in order of frequency, most frequent chemo-related SAEs are provided on the top of the table.

Title of chemotherapy-related SAE	Grade	Related to oxaliplatin	Related to 5-Fluorouracil	Number of SAEs reported with same characteristics
Acute kidney injury	5	yes	yes	1
Acute onset chronic renal failure	3	yes	no	1
Community acquired pneumonia	Not determined	yes	yes	1
Dehydration and anaemia	3	yes	yes	1
Diarrhoea	2	yes	yes	1
Diarrhoea	3	yes	yes	2
E. Coli Infection	3	yes	yes	1
Febrile neutropenia	3	yes	yes	1
Febrile neutropenia	5	yes	yes	1
Febrile neutropenia and chest infection	4	yes	yes	1
Fever (non neutropenic)	2	yes	yes	1
Heart attach	4	no	yes	1
Hepatitis (acute)	3	yes	no	1
Lung infection	3	yes	yes	1
Lung infection	5	yes	yes	1
Neutropenic sepsis	3	yes	yes	1
Neutropenic sepsis	4	yes	yes	1
PICC line infection (catheter associated infection)	3	yes	yes	1
PICC line infection (catheter associated infection)	2	yes	yes	1
Respiratory infection	3	yes	yes	1
Sepsis	4	yes	yes	2
Sepsis (presumed chest or biliary)	2	yes	yes	1
Thromboembolic event	2	yes	yes	1
Urinary tract infection	3	yes	yes	1
Vomiting	2	yes	yes	1
Vomiting	3	yes	no	1
Vomiting	3	yes	yes	1
TOTAL	n/a	n/a	n/a	29 SAEs (21 patients)

Section H: Subsequent lines of systemic therapy

N: number of patients; %: percentage; pts: patients; ASC: active symptom control; FOLFOX: 5-FU+oxaliplatin; CisGem: cisplatin and gemcitabine; CAPOX: capecitabine + oxaliplatin; FOLFIRI: 5-fluorouracil + irinotecan; 5-FU: 5-fluorouracil.

*1 patient was treated with: 2nd and 3rd line FOLFOX + 4th line capecitabine; &1 patient received CisGem (4th line); @one patient entered two separate phase I clinical trials; & one patient received CisGem after phase I trial

Subsequent line of systemic anti-cancer therapy	Total n=162		Arm A (ASC alone) n=81 pts		Arm B (ASC + FOLFOX) n=81 pts	
	n	%	n	%	n	%
**Yes**	21	13	11	14	10	12
**Phase I**	5	3	3@	4	2&	2
**Chemotherapy**	16	10	8	10	8	10
FOLFOX/CAPOX	7	4	5*	6	2	2
CisGem	3	2	2	2	1	1
FOLFIRI	1	1	0	0	1&	1
Gemcitabine + Carboplatin	1	1	0	0	1	1
Gemcitabine	1	1	0	0	1	1
5-Fluorouracil	1	1	0	0	1	1
Irinotecan	1	1	0	0	1	1
Dendritic cell vaccine therapy	1	1	1	1	0	0

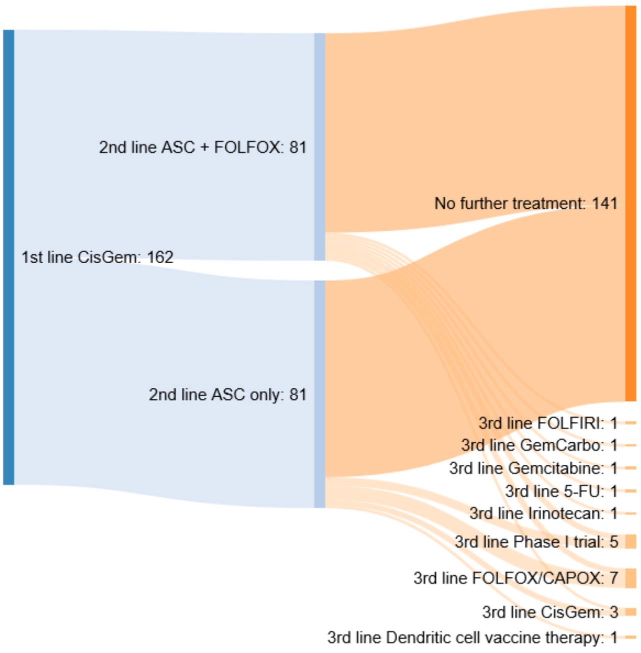

Absolute number of patients are provided. Two patients received fourth-line therapy: one patient was treated with second-line and third-line FOLFOX plus fourth-line capecitabine, whereas the second patient received cisplatin and gemcitabine as fourth-line therapy. ASC=active symptom control. CAPOX=capecitabine and oxaliplatin. FOLFIRI=fluorouracil and irinotecan. FOLFOX= FOLFOX=folinic acid, fluorouracil, and oxaliplatin.

Section I: Summary of population characteristics and patient outcome for ABC-06 and other recent studies exploring the role of second –line therapy in advanced biliary cancer.

Reported outcome (A) and baseline characteristics (B) including primary tumour site and line of treatment for each study arm are provided. Data extracted from (6, 31, 34, 35).

PFS: progression-free survival; OS: overall survival; n: number of patients; FOLFOX: 5-fluorouracil and oxaliplatin; ASC: active symptom control; 5: percentage; NR: not reported; iCCA: intrahepatic cholangiocarcinoma; BTC: biliary tract cancer; AMP: ampullary cancer; GBC: gallbladder cancer; eCCA: extrahepatic cholangiocarcinoma; NOS: not otherwise specified, includes eCCA where distinction between hCCA and dCCA is not made; hCCA: hilar cholangiocarcinoma; dCCA: distal cholangiocarcinoma; FOLFIRINOX: 5-fluorouracil, irinotecan, and oxaliplatin.

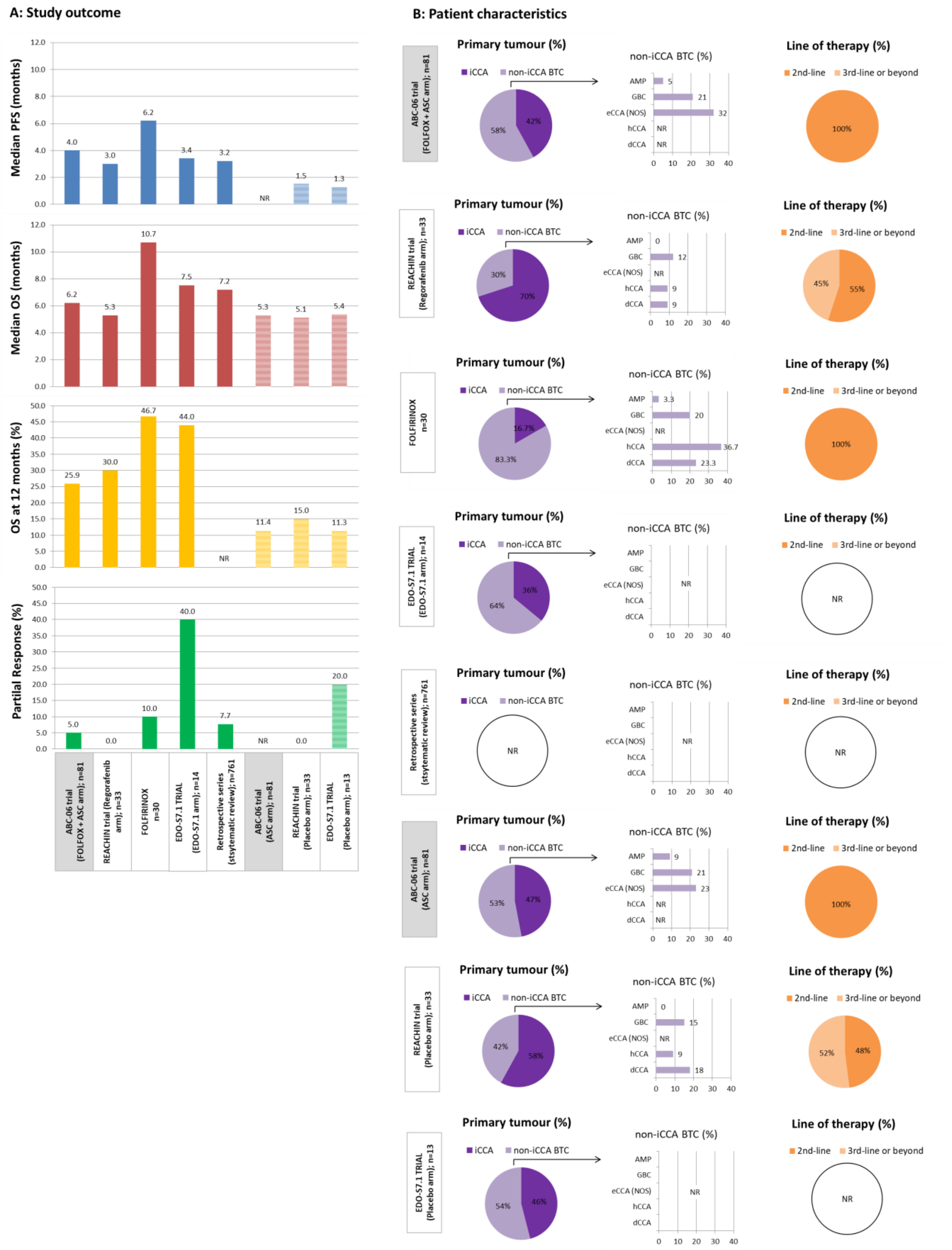
A: Study outcome
B: Patient characteristics
Median PFS (months)
4.0
3.0
6.2
3.4
3.2
NR
1.5
1.3
Median OS (months)
6.2
5.3
10.7
7.5
7.2
5.3
5.1
5.4
OS at 12 months (%)
25.9
30.0
46.7
44.0
NR
11.4
15.0
11.3
Partial Response (%)
5.0
0.0
10.0
40.0
7.7
NR
0.0
20.0
ABC-06 trial (FOLFOX + ASC arm); n=81
REACHIN trial (Regorafenib arm); n=33
FOLFIRINOX n=30
EDO-S7.1 TRIAL (EDO-S7.1 arm); n=14
Retrospective series (stsytematic review); n=761
ABC-06 trial (ASC arm); n=81
REACHIN trial (Placebo arm); n=33
EDO-S7.1 TRIAL (Placebo arm); n=13
Primary tumour (%)
iCCA
non-iCCA BTC
non-iCCA BTC (%)
AMP
GBC
eCCA (NOS)
hCCA
dCCA
Line of therapy (%)
2nd-line
3rd-line or beyond
NR

Section J: List of recruiting sites and principal investigators

Site	Principal Investigator	No of participants recruited
The Christie Hospital, Manchester	Prof Juan Valle	33
The Clatterbridge Cancer Centre, Wirral	Prof Daniel Palmer	18
Hammersmith Hospital, London (Imperial College)	Dr Harpreet Wasan	17
Guy’s & St Thomas’ Hospital, London	Dr Paul Ross	14
University College London - Cancer Clinical Trials Unit	Prof John Bridgewater	13
Queen Elizabeth Hospital, Birmingham	Dr Yuk Ting Ma	12
Nottingham City Hospital	Dr Arvind Arora	10
Bristol haematology & oncology centre	Dr Stephen Falk	9
Royal Free Hospital, London	Dr Roopinder Gillmore	8
Weston Park Hospital, Sheffield	Dr Jonathan Wadsley	8
Churchill Hospital, Oxford	Dr Kinnari Patel	7
St James' Hospital, Leeds	Dr Fiona Collinson	5
Castle Hill Hospital, Hull	Dr Anthony Maraveyas	3
Great Western Hospital, Swindon	Dr Claire Hobbs	2
Maidstone Hospital, Kent	Dr Justin Waters	2
Southampton General Hospital	Dr Tim Iveson	1

Section K: Study protocol

ABC-06

A Phase III, randomised, multi-centre, open-label study of active symptom control (ASC) alone or ASC with oxaliplatin/5-FU chemotherapy for patients with locally advanced/metastatic biliary tract cancers previously treated with cisplatin / gemcitabine chemotherapy

A MCTU Managed Trial
Sponsored by The Christie NHS Foundation Trust
Funded by Cancer Research UK
Chief Investigator: Professor Juan W Valle

Under the auspices of the NCRN Upper GI Clinical studies group.

EudraCT: 2013-001812-30
Sponsor reference number: CFTSp048
ClinicalTrials.gov Identifier: NCT01926236

Protocol version: V7.0 03 DEC 2018

TRIAL MANAGEMENT GROUP

Role in Trial	Name	Postal Address	Telephone and Fax Number(s)	Email address
Chief Investigator	**Professor Juan W Valle**<br>Consultant/Honorary Professor in Medical Oncology	The University of Manchester and The Christie NHS Foundation Trust; Wilmslow Road; Manchester, M20 4BX	Tel: 0161 446 3719<br>Fax: 0161 446 8234	juan.valle@christie.nhs.uk
Co-Investigator	**Dr John Bridgewater**<br>Senior Lecturer and Consultant in Medical Oncology	UCL Cancer Institute, 72 Huntley St, London WC1E 6AA	Tel: 020 7679 6080<br>Fax: 020 7679 6925	j.bridgewater@ucl.ac.uk
Co-Investigator	**Professor Daniel Palmer**<br>Professor of Medical Oncology	Department of Molecular and Clinical Cancer Medicine, Duncan Building, Daulby Street, University of Liverpool, Liverpool L69 3GA	Tel: 0151 706 4177<br>Fax: 0151 706 5826	Daniel.Palmer@liverpool.ac.uk
Co-Investigator	**Dr Harpreet Wasan**<br>Consultant & Reader in Oncology	Imperial College Healthcare NHS Trust, Hammersmith Hospital, DuCane Road, London W12 0HS	Tel: 020 8383 3057<br>Fax: 020 8383 3054	h.wasan@imperial.ac.uk
Co-Investigator (Health Economist)	**Professor Linda Davies**	Manchester Centre for Health Economics, Institute of Population Health, University of Manchester, Jean MacFarlane Building, Oxford Road, Manchester,<br>M13 9PL	Tel: 0161 306 7907	linda.m.davies@manchester.ac.uk
Co-Investigator<br>(QoL advisor)	**Dr John Ramage**<br>Consultant Gastroenterologist	Hampshire Hospitals NHS Trust<br>Aldermaston Road<br>Basingstoke<br>RG24 9NA	Tel: 01256 313637	john.ramage@hhft.nhs.uk
Sponsor's representative	**Gillian Heap**<br>Head of Research Operations	R&D Department The Christie NHS Foundation Trust Wilmslow Road<br>Manchester, M20 4BX	Tel: 0161 918 7432, Fax: 0161 446 8201	gillian.heap@christie.nhs.uk
Statistician	**David Ryder**<br>Statistician	Manchester Clinical Trials Unit (MCTU), Jean McFarlane Building, The University of Manchester, Oxford Road, Manchester M13 9PL	Tel: 0161 275 7688	David.Ryder@manchester.ac.uk
Clinical Trial Project Manager	**Safia Barber**<br>Clinical Trials Project Manager	**Manchester Clinical Trials Unit (MCTU)**<br>Jean McFarlane Building, 1st floor, Room 1.309, The University of Manchester, Oxford Road, Manchester M13 9PL**MCTU**	Tel: 0161 9306 8036	ABC06@manchester.ac.uk
Lead Pharmacist	**Alkesh Patel**<br>Senior Clinical Trials Pharmacist	The Christie NHS Foundation Trust Wilmslow Road; Manchester, M20 4BX	Tel: 0161 918 2380	alkesh.patel@christie.nhs.uk
Lead Laboratory	The Christie NHS Foundation Trust Wilmslow Road; Manchester, M20 4BX			

Role in Trial	Name	Postal Address	Telephone and Fax Number(s)	Email address
Lead medical / technical department	The Christie NHS Foundation Trust Wilmslow Road; Manchester, M20 4BX			
Biobank Management	**Jane Rogan**<br>Business Manager	Manchester Cancer Research Centre Biobank; Paterson Institute for Cancer Research; M20 4BX, Manchester	Tel: 0161 446 3659, Fax: 0161 446 3203	jane.rogan@christie.nhs.uk
Protocol development	**Dr Angela Lamarca**<br><br>ESMO Clinical Fellow	The Christie NHS Foundation Trust; Wilmslow Road; Manchester, M20 4BX	Tel: 0161 446 3719, Fax: 0161 446 8234	angela.lamarca@christie.nhs.uk

Trial email address for general queries/ correspondence to MCTU:

ABC06@manchester.ac.uk

DISCLAIMER

This document describes the ABC-06 trial and provides information about procedures for entering patients into it. The protocol should not be used as a guide for the treatment of patients outside the trial. Every care was taken in drafting this protocol, but corrections or amendments may be necessary. These will be circulated to all Investigators participating in the trial; centres entering patients for the first time are advised to contact the Trial Manager at the MCTU to confirm they have the most recent and approved version.

This trial will adhere to the principles outlined in the Medicines for Human Use (Clinical Trials) regulations 2004 and ICH Good Clinical Practice guidelines. The trial will be conducted in compliance with the protocol, the Data Protection Act, the Declaration of Helsinki, Human Tissue Act (2004), the Research Governance Framework (2005) and other regulatory requirements as appropriate.

Protocol Authorised by:

Name & Role	Signature	Date
**Professor Juan W Valle**<br>Chief Investigator		
**Gillian Heap**<br>Sponsor representative		

Principal Investigator

I, as Principal Investigator for the ABC-06 trial for the site stated below, confirm that I will be responsible to ensure that all members of the local clinical trial team are appropriately trained on the trial protocol and have the relevant qualifications and experience to carry out their role in accordance with the trial protocol.

Name	Site	Signature	Date

TABLE OF CONTENTS

DISCLAIMER 3

LIST OF ABBREVIATIONS 8

1.-TRIAL SUMMARY 10

2.-BACKGROUND 13

2.1- BILIARY TRACT CANCERS 13
2.2.-TREATMENT FOR ADVANCED BILIARY TRACT CANCER 13
2.2.1.-Second-line chemotherapy, current evidence 15
2.3.-RATIONALE FOR SELECTING THE MFOLFOX REGIMEN 17
2.4.-RATIONALE FOR FACTORS CONTROLLED FOR AT RANDOMISATION 18
2.5.-SUMMARY 19

3.-TRIAL OBJECTIVES AND ENDPOINTS 19

3.1.-TRIAL OBJECTIVES 19
3.2.-TRIAL ENDPOINTS 19

4.-TRIAL DESIGN 20

4.1.-OVERALL DESIGN 20
4.2.-SELECTION OF TRIAL PARTICIPANTS 21
4.2.1.-Inclusion criteria 21
4.2.2.-Exclusion criteria 22
4.2.3.-Concomitant medication 23

5.-RECRUITMENT OF TRIAL PARTICIPANTS 23

5.1.-SELECTION OF CENTRES/CLINICIANS 24
5.2.-IDENTIFYING PARTICIPANTS 24
5.3.-CONSENTING PARTICIPANTS 24
5.4.-SCREENING FOR ELIGIBILITY, PRE-RANDOMISATION EVALUATION 26
5.5.-INELIGIBLE AND NON-RECRUITED PARTICIPANTS 27

6.-REGISTRATION AND RANDOMISATION PROCEDURES 27

7.-DISCONTINUATION/WITHDRAWAL OF PARTICIPANTS AND "STOPPING RULES" 28

7.1.-WITHDRAWAL OF PATIENTS 29
7.2.-EARLY DISCONTINUATION OF TRIAL 29
7.3.-WITHDRAWAL FROM TRIAL PARTICIPATION BY SITES 29

8.-TREATMENT DETAILS 30

8.1.-TREATMENT SUMMARY 30
8.2.-DETAILS OF TRIAL TREATMENT 31
8.2.1.-mFOLFOX and suggested premedication: administration, dosage, periods 31
8.2.2.-Overdose 32
8.2.3.-Dose Changes and Modification of trial treatment 33
8.3.-MANAGEMENT AND HANDLING OF INVESTIGATIONAL MEDICAL PRODUCT (IMP) 38
8.4.-NON-STUDY TREATMENT 39
8.4.1.-CONCOMITANT TREATMENT 39
8.4.2.-Interaction with Other Drugs 39

9.-TRIAL ASSESSMENTS 40

9.1.-SCREENING ASSESSMENTS 40
9.2.-IMAGING ASSESSMENTS 40
9.3.-ASSESSMENTS TO BE PERFORMED IN ARM A 40
9.4.-ASSESSMENTS TO BE PERFORMED IN ARM B 41

10.-TRIAL CLOSURE 44

11.-STATISTICS AND DATA ANALYSIS 45

11.1.-SAMPLE SIZE CALCULATION 45
11.2.-THE RECRUITMENT RATE 46
11.3.-DISCONTINUATION RULES 46
11.4.-PRIMARY ANALYSIS 46
11.5 SECONDARY ANALYSIS 47
11.6 ECONOMIC ANALYSIS 47
11.6.1 Measure of health benefit *47*
11.6.2 ***Resource use and costs*** *48*
11.6.3 ***Missing data*** *48*
11.6.4 Economic data analysis *49*

12.-PHARMACOVIGILANCE 51

12.1.-DEFINITIONS 51
12.2.-DETECTING AND REPORTING AEs 52
12.2.1.-SAE Processing and Reporting at the MCTU 53
12.2.2.-Detecting and reporting a SUSAR 54
12.3.-PREGNANCY REPORTING 55
12.4.-NEW SAFETY FINDINGS 56
12.5.-URGENT SAFETY MEASURES 56
12.6.-PERIODIC SAFETY REPORTS 56

13.-TRIAL MANAGEMENT AND OVERSIGHT ARRANGEMENTS 57

13.1.-OVERSIGHT COMMITTEES 57
13.1.1.-Trial Management Group (TMG) 57
13.1.2-Trial Steering Committee (TSC) 57
13.1.3.-Independent Data Monitoring Committee (IDMC) 58
13.2.-TRIAL MANAGEMENT: MCTU 59
13.2.1.-Data Collection 59
13.2.2.-Data Handling at MCTU 59
13.2.3.-Trial monitoring 60

14.-ETHICAL AND REGULATORY REQUIREMENTS 60

14.1.-SPONSORSHIP AND INDEMNITY 61
14.2.-PARTICIPANT INFORMATION AND INFORMED CONSENT 61
14.3.-PARTICIPANT CONFIDENTIALITY AND DATA PROTECTION 62
14.4.-DIRECT ACCESS TO DATA 63

15.-TRIAL CONDUCT 63

15.1.-PROTOCOL AMENDMENTS 63
15.2.-PROTOCOL DEVIATIONS AND SERIOUS BREACHES 63

16.-END OF TRIAL 64

17.-TRANSLATIONAL RESEARCH 64

18.-PEER REVIEW **65**

19.-PUBLICATION POLICY **65**

20.-TRIAL RECORD RETENTION AND ARCHIVING **65**

APPENDICES **66**

APPENDIX 1: PERFORMANCE STATUS (ECOG) 66
APPENDIX 2: COCKCROFT-GAULT, DUBOIS AND GLOBULIN EQUATIONS 67
GLOBULIN EQUATION 67
TOTAL GLOBULIN = TOTAL PROTEIN - ALBUMINAPPENDIX 3: NEW YORK HEART ASSOCIATION STAGING (NYHA) 67
APPENDIX 4: EXPECTED TOXICITY PROFILE OF STUDY MEDICATIONS AND SUGGESTED RECOMMENDATIONS TO PATIENT 69
APPENDIX 5: TRIAL TREATMENT: SUGGESTED DOSAGE AND PREMEDICATION 71
APPENDIX 6: SUGGESTED DOSE REDUCTIONS 72
APPENDIX 7: TRIAL ASSESSMENTS 73
APPENDIX 8: TRANSLATIONAL BLOODS COLLECTION 75
APPENDIX 9: FLOWCHART FOR ADVERSE EVENT NOTIFICATION 76

LIST OF ABBREVIATIONS

5FU	5-fluorouracil
AE	Adverse Event
ABC	Advanced Biliary tract Cancer
ALT	Alanine Aminotransferase
ANC	Absolute Neutrophil Count
APTT	Activated Partial Thromboplastin Time
AR	Adverse Reaction
AST	Aspartate Aminotransferase
ASC	Active Symptom Control
BSC	Best Supportive Care
BTC	Biliary Tract Cancer
CI	Chief Investigator
CRF	Case Report Form
CRP	C-reactive protein
CT	Computerized tomography
CTA	Clinical Trial Authorisation
CTAAC	Clinical Trials Awards and Advisory Committee
CTCAE	Common Terminology Criteria for Adverse Events
CTIMP	Clinical Trial Investigational Medicinal Product
CTPM	Clinical Trial Project Manager
DMC	Data Monitoring Committee
Dox	doxorubicin
EC	European Commission
ECF	epirubicin, cisplatin and 5FU
EU	European Union
EudraCT	European Clinical Trials Database
FELV	5FU, etoposide and leucovorin
FOLFOX	Combination of 5FU and oxaliplatin
mFOLFOX	Modified combination of 5FU and oxaliplatin
GCP	Good Clinical Practice
G-CSF	Granulocyte colony-stimulating factor
Gem	Gemcitabine
mGEMOX	modified gemcitabine and oxaliplatin
GP	General Practitioner
HR	Hazard Ratio
IDMC	Independent Data Monitoring Committee
IMP	Investigational Medicinal Product
INR	International normalised ratio
MCTU	Manchester Clinical Trials Unit
MHRA	Medicines and Healthcare products Regulatory Agency
MMC	mitomycin C
MR	Magnetic Resonance
MRCP	Magnetic resonance cholangiopancreatography
NCCN	National Comprehensive Cancer Network
NCRN	National Cancer Research Network
PI	Principal Investigator

PS	Performance status
RECIST	Response Evaluation Criteria In Solid Tumors
SAR	Serious Adverse Reaction
SAE	Serious Adverse Event
SAP	Statistical Analysis Plan
SmPC	Summary of Product Characteristics
SUSAR	Suspected Unexpected Serious Adverse Reaction
TMG	Trial Management Group
TSC	Trial Steering Committee
ULN	Upper Limits of Normal
WBC	White Blood Count

1.-TRIAL SUMMARY

**Title:**	ABC-06: A Phase III, randomised, multi-centre, open-label study of active symptom control (ASC) alone or ASC with oxaliplatin/5-FU chemotherapy for patients with locally advanced/metastatic biliary tract cancers previously treated with cisplatin / gemcitabine chemotherapy
**Design:**	Randomised (1:1), controlled, two-arm, open-label study<br><br>Multi-centre study (under the auspices of the NCRN Upper GI Clinical studies group (Hepatobiliary Sub-group)).
**Objectives:**	This study will determine whether fit patients (with performance score of 0-1) with advanced biliary tract cancer (ABC) benefit from chemotherapy in the second-line setting (after prior therapy with cisplatin and gemcitabine) in terms of overall survival.<br><br>In addition we will determine the radiological response rate, effect on patients' quality of life and health economics of using chemotherapy in this setting. Clinical samples will be collected for future translational work.
**Endpoints**	Primary endpoint:<br><br>• Overall survival<br><br>Secondary endpoints:<br><br>• Progression-free survival<br>• Response rate (chemotherapy arm only)<br>• Toxicity<br>• Quality of life (QLQ-C30 – validated and QoL Bil – exploratory)<br>• Costs of health and social care<br>• Health status (Euroqol)<br>• Quality adjusted life years (QALYs) estimated from Euroqol and survival using published utility tariffs
**Cohorts:**	162 patients (81 on each arm)<br><br>• ARM A: Active Symptom Control.<br>• ARM B: ASC + mFOLFOX chemotherapy
**Eligibility**	Please refer to section 4.2 for full eligibility criteria<br><br>Inclusion Criteria:<br><br>✓ Histologically / cytologically verified, ABC failed no more than one prior course of chemotherapy (gemcitabine and cisplatin) with clear evidence of disease progression<br>✓ ECOG performance status 0-1<br>✓ Adequate haematological, renal and liver function.<br>✓ Adequate biliary drainage, with no evidence of ongoing infection.

	✓ Life expectancy >3 months<br>✓ All patients must be randomised and sites must ensure that patients allocated chemotherapy (ARM B) start treatment within 6 weeks of radiological progression<br><br><u>Exclusion Criteria</u>:<br><br>✗ Incomplete recovery from previous therapy (includes ongoing neuropathy of grade >1 from cisplatin)<br>✗ Clinical evidence of metastatic disease to brain<br>✗ Clinically significant cardiovascular disease.
**Trial Treatment and methods:**	Consenting patients with histologically / cytologically-verified advanced (locally advanced or metastatic) biliary tract cancer with evidence of disease progression after prior cisplatin /gemcitabine chemotherapy will be randomised (1:1) to either ASC (the "standard" arm) or ASC with oxaliplatin/5-FU chemotherapy (the "experimental" arm).<br><br>**ARM A**: Active Symptom Control.<br><br>**ARM B**: Active Symptom Control + mFOLFOX chemotherapy:<br><br>- Oxaliplatin 85mg/m²,<br>- L-folinic acid 175mg or 350 mg folinic acid,<br>- 5-FU 400mg/m² (bolus) and<br>- 5-FU 2400mg/m² (infusion) q14d.<br><br>Patients will undergo tumour evaluation by CT/MR scan every 12 weeks; patients allocated to receive chemotherapy will be seen for treatment every 2 weeks; chemotherapy will continue (in the absence of disease progression, intolerable toxicity or patient choice to withdraw) to a maximum of 12 cycles (6 months).<br><br>- All patients are to be followed up to the point in calendar time 12 months after randomisation of the last patient, or until death, whichever is earlier.<br><br>- Quality of life assessments will be performed at baseline and 12-weekly thereafter.<br><br>- Health economics will also be formally assessed.
**Trial duration per participant:**	All patients followed until 12 months after enrolment of the final patient, or until death, whichever comes first (up to 42 months follow-up for the first patients enrolled).
**Estimated total trial duration:**	45 months: duration of trial set up (3 months), duration of trial recruitment (30 months), duration of trial follow-up (12 months).
**Total number of participants planned:**	162 patients (81 on each arm)

**Additional trials/ Sub-trials**	- Archival paraffin-embedded tissue will be collected at baseline. Prospective blood samples (whole blood, serum and plasma) will be collected for translational research (subject to separate protocol and regulatory approvals):<br>o At baseline (both arms)<br>o At 12 weeks (both arms)<br>o At disease progression (experimental arm only)

2.-BACKGROUND

2.1- Biliary tract cancers

Biliary tract cancers (BTCs) are relatively low-incidence (0.7%) malignant tumours in adults with approximately 1200 new cases registered in England and Wales per year (1)); however they are associated with a high mortality rate (approximately 23 per million population (2)).

They are sub-classified with respect to site of origin as:

- Gallbladder cancer (40%)
- Extra-hepatic cholangiocarcinoma (35%)
- Ampullary tumours (13%)
- Intra-hepatic cholangiocarcinoma (8%)
- Unspecified/other cancers (4%)

BTCs are more prevalent in patients between 50 and 70 years, with a higher incidence in males in the case of cholangiocarcinoma and ampullary carcinomas and in females for gallbladder cancers. Although adenocarcinoma histology accounts for more than 90%, it is possible to find other histological subtypes such as squamous, neuroendocrine tumours, lymphomas or sarcomas. The main risk factors for BTC are gallstones, congenital abnormalities of the bile ducts, primary sclerosing cholangitis, chronic liver diseases and hereditary polyposis syndromes. (3;4).

BTCs present the combined clinical problems of biliary obstruction and malignancy, causing obstructive jaundice, right upper quadrant pain and weight loss. Hepatic dysfunction can occur as a consequence of unrelieved biliary obstruction as well as local invasion of the vascular supply. Unfortunately sepsis is a frequent cause of death. This is usually consequent to inadequate biliary drainage (despite intervention) and it is important in the management of these conditions that the combination of obstruction and infection is treated pro-actively.

2.2.-Treatment for advanced biliary tract cancer

Surgery offers the only chance of long-term cure; however, due to the aggressive nature of BTC, most of patients (>65%) are diagnosed in advanced stages, when no surgery is feasible and when palliative chemotherapy is the only treatment available.

The prognosis of patients diagnosed with advanced (metastatic or unresectable locally advanced disease) biliary cancer is poor. The five-year overall survival for stage III and IV is 10% and 0%, respectively (5). Hence supportive care is pivotal in the management of patients with advanced biliary

tract cancer (ABC) from the point of diagnosis. Nevertheless, first line palliative chemotherapy has shown improvement in overall survival and quality of life.

The most active chemotherapy drugs for the treatment of ABC are gemcitabine, fluoropyrimidines and platinum agents (6;7). The UK NCRN ABC-02 study (8) established cisplatin and gemcitabine as the reference regimen for the first-line treatment of patients with ABC. Results from this randomized phase III study with 410 patients comparing cisplatin and gemcitabine doublet-chemotherapy over gemcitabine monotherapy demonstrated advantage in overall survival (median 11.7 vs. 8.1 months; p<0.001) and in progression-free survival (median 8 vs. 5 months; p<0.001). A very similar magnitude of benefit was seen in a Japanese randomized phase II study using the same treatment regimens (the BT-22 study) where a median survival of 11.2 months was documented with cisplatin / gemcitabine (9).

Although other chemotherapy agents appear promising in phase II studies, they have not been validated in prospective phase III trials (6). Only six phase III trials have been conducted in this context (1st-line chemotherapy for advanced disease):

**Author**	**No**	**Survival**	**Chemotherapy**
Glimelius 1998	37	6 vs 2.5m	5FU/Etop vs. BSC
Takada 1998	31	6 vs 3m	5FU/Dox/MMC vs BSC
Kornek 2004	51	6.7 vs 9.3m	MMC/Gem vs MMC/Capecitabine
Ducreux 2005	58	5 vs 8m	5FU vs. Cis/5FU
Rao 2005	54	12 vs 9m	FELV vs ECF (planned phase III trial but terminated early)
Sharma 2010	81	4.5m vs 4.6m vs 9.5m	BSC/ FU & folinic acid/ mGEMOX

In 2005 a phase III study comparing 5FU, etoposide and leucovorin (FELV) to epirubicin, cisplatin and 5FU (ECF) in first line ABC patients was conducted (10). Fifty-four patients were recruited with 27 randomly assigned to each arm. No difference between arms in overall survival, progression-free survival and objective response rate were shown, although the study closed prior to complete enrolment (slow recruitment rate) and was consequently statistically underpowered. In 2010 another phase III trial evaluated efficacy of modified gemcitabine and oxaliplatin (mGEMOX) (Arm A) over BSC (Arm B) or FU in combination with folinic acid (Arm C) in unresectable gall bladder cancer (11). A total of 81 patients were included in this trial; the median overall survival and progression free

survival with mGEMOX were significantly superior to BSC. Nevertheless, there was no difference between the BSC and FU. This data suggested that GEMOX-based chemotherapy was superior not only to BSC but also FU in overall survival and also in progression free survival.

Given the robustness of the ABC-02 study with respect to its size and observed survival advantage, cisplatin and gemcitabine is now considered the standard of care and has been widely adopted in the UK and internationally (e.g. NCCN guidelines in USA).

2.2.1.-Second-line chemotherapy, current evidence

Although there are multiple trials evaluating the efficacy and tolerability of chemotherapy in first line treatment for ABC, there is no phase III evidence to date supporting the use of second-line treatment after progression to cisplatin and gemcitabine in ABC. As detailed above, patients often have the inherent problems of biliary obstruction and sepsis with ABC, which limit survival and quality of life and may make second-line treatment difficult to deliver.

To date, active symptom control (ASC) is the standard of care after progression to first line chemotherapy. ASC includes addressing obstructive and infective problems (e.g. by biliary stenting and antibiotics, as appropriate) and other symptoms arising from tumour progression, aimed at improving the quality of life for these patients.

Some patients progressing after first-line chemotherapy have evidence of rapidly worsening performance status; however, some patients still have a good performance score and may be suitable for salvage chemotherapy. In the ABC-02 trial (8), from the total of 410 patients treated with first-line chemotherapy, only sixty-three patients (15.3%) were treated with second line chemotherapy (12). This is partly due to the lack of an available second-line regimen. In contrast, 63 of the 84 patients (75%) included in the Japanese BT22 trial (9) received second line chemotherapy (where there was an available treatment option: S1). Despite this difference in the rate of patients going on to receive second line chemotherapy, the median overall survival of both studies was very similar (11.7 and 11.2 months in ABC-02 and BT22, respectively).

Several retrospective studies have been reported exploring the potential benefit of second-line chemotherapy:

- A large retrospective study of 378 ABC patients analyzed the tolerance, survival and prognostic factors of 96 ABC patients going on to receive second-line chemotherapy (13). Various chemotherapy regimens were used including gemcitabine monotherapy, gemcitabine-based combination, 5FU or capecitabine monotherapy, a combination of gemcitabine and

5FU, other 5FU-based schedules, or regimens containing other drugs (irinotecan, docetaxel, paclitaxel, epirubicin, erlotinib) along with phase 1 studies. Nine percent of the patients had an objective response with second line treatment and 34% achieved stable disease. The median progression free survival was 2.8 months and the overall survival was 7.5 months. However, by its nature, the data reported in this retrospective analysis is too heterogeneous (multiple chemotherapy schedules employed) to conclude about the usefulness of second-line chemotherapy in general or an individual regimen, in particular, for ABC. Only 25% of the patients on treatment with first line chemotherapy received second line systemic chemotherapy; young patients, females and patients with a PFS > 6 months following first line chemotherapy were more likely to receive it. Several prognostic factors were related with overall survival after a second line chemotherapy regimen: performance status > 1 (HR 0.43 (0.21–0.91), p = 0.03), gallbladder cancer (HR 0.43 (0.31–0.60), p < 0.0001), objective response as best response with first line schedule (HR 2.09 (1.47–2.97), p < 0.0001) and PFS with first line longer than 6 months (HR 4.27 (3.06–5.95), p < 0.0001).

- Another retrospective analysis of 63 patients previously treated with a combination of gemcitabine, were treated with second line chemotherapy (gemcitabine, 5FU, mFOLFOX or cisplatin-gemcitabine) (12). The progression-free survival and overall survival were 4 and 8.1 months respectively.

Only a few prospective clinical trials involving salvage treatment for BTC have been conducted; both were after treatment with either gemcitabine or 5FU in first-line.

- In a phase II clinical trial, 32 ABC patients were treated with second-line gemcitabine after progression on 5FU (14); of the 29 evaluable patients, two achieved a partial response, with an overall response rate of 6.9%. According to prognostic factor analysis, poor performance status (PS >1) and albumin level <3.5 g/dL had an independent influence on inferior time to progression and overall survival.
- A phase II trial with gemcitabine pre-treated patients diagnosed with either pancreatic or BTC was performed (15). The treatment employed was a combination schedule of 5FU, doxorubicin and mitomycin-C (FAM). Sixteen patients with ABC were treated in this trial (of a total of 31 patients). Only performance status of patients correlated with overall survival. The results of this study showed that a combination of infusional 5-FU is both feasible and tolerable in patients with advanced pancreatic and biliary tract cancer after gemcitabine-based chemotherapy.
- Treatment with capecitabine and celecoxib in a phase II trial reported a response rate of 9% and a median overall survival of 19 weeks (4.4 months) in the 55 patients treated (16).
- Treatment with S-1 after progression to gemcitabine in a phase II trial was reported in 2012 (17). In 22 patients, S-1 single-agent was feasible and moderately efficacious second-line

chemotherapy. The median overall survival time was 13.5 months and the median time-to-progression was 5.4 months.

- Targeted therapies have also been studied in second line treatment after progression to gemcitabine or 5FU. In a phase II trial with sunitinib, a marginal efficacy in second line ABC patients was suggested (18); everolimus (19) and imatinib (20) have also been tested without very encouraging results in monotherapy.

This limited data suggests that there is a population suitable for second-line prospective randomized studies with chemotherapy as recommended in the recent retrospective series (13) although there is no level 1 evidence of benefit in this patient population.

2.3.-Rationale for selecting the mFOLFOX regimen

In this trial the aim is to determine if patients with ABC benefit from mFOLFOX chemotherapy in the second-line setting, after progression to first-line treatment with cisplatin and gemcitabine.

Three groups of agents have broadly shown activity in biliary tract cancer in retrospective and prospective trials: gemcitabine, fluoropyrimidines and platinum agents (6). Moreover, the sensitivity to a platinum agent has been recently confirmed in the phase III ABC-02 trial, in which cisplatin and gemcitabine combination arm shown benefit in survival compared to gemcitabine alone. After progressing to a first line gemcitabine-based chemotherapy switching to a fluoropyrimidine-based schedule is considered appropriate. A previous pooled analysis suggests that patients receiving doublet-chemotherapy have a greater benefit vs. monotherapy (6). Given the known platinum sensitivity (from ABC-02), it is anticipated that a 5-FU / platinum doublet is most likely to be effective.

The third-generation platinum analogue oxaliplatin is known for its activity in several gastrointestinal tumours and a synergistic activity with a favourable toxicity profile seems to exist with its combination with 5FU.

In twenty-nine patients with locally advanced or metastatic BTC treated with single agent oxaliplatin an objective response rate of 20.6% was shown (21), thus oxaliplatin appears to be an active agent against BTC. Additional studies using Oxaliplatin/ 5FU based regimens for biliary tract tumours are available, with good results and acceptable toxicity (22-24). In 2008 a phase II trial in 28 patients (including pre-treated and chemotherapy-naïve) patients treated with FOLFOX achieved a response rate of 21.5% and median overall survival of 10 months (25). In another prospective analysis of sixteen patients diagnosed with ABC, FOLFOX achieved a disease control rate (PR + stable disease) of 56% and a median overall survival of 9.5 months (26).

2.4.-Rationale for factors controlled for at randomisation

Patients will be randomised to a trial arm using the allocation method of minimisation with a random element. The factors controlled for in the allocation will be:

- Platinum sensitivity (refractory and resistant patients vs. sensitive patients)
- Serum albumin (<35g/l vs. ≥35g/l)
- Locally advanced vs. metastatic disease

The prognostic effect of ***PS*** and ***albumin levels*** have been demonstrated in several trials and retrospective analyses (13-15). Patients progressing after first-line chemotherapy who have a rapidly worsening performance status are not likely to benefit from second-line treatment. Thus, only patients with good performance status (PS 0-1) are eligible and serum albumin levels will be controlled for at randomisation.

Platinum sensitivity and resistance have not been defined in ABC. In one retrospective analysis, the cut-off of six months progression-free survival seemed to be a prognostic factor in the second-line setting (13;27). Using the median PFS as a cut-off in first-line chemotherapy, as it is done in other malignancies (ovarian cancer, small cell lung cancer and germinal tumours) may be useful. The median PFS ABC-02 was 8 months from randomization (patients received 6 months of treatment). Therefore, in this study we will use the following definitions:

1. **Refractory disease**: radiologically confirmed progressive disease during first line treatment with cisplatin and gemcitabine
2. **Resistant disease:** radiologically confirmed progressive disease within the first three months (90 days) after completion of the last cycle of first line chemotherapy* (likely to coincide with timing of follow-up scan)
3. **Sensitive disease**: radiologically confirmed progressive disease after three months (90 days) of the last cycle of first line-chemotherapy*.

* Date of 'completion of last cycle of first line chemotherapy' is defined as the day 1 date of the last cycle given.

Platinum sensitivity *will be controlled for at randomisation*, as this is thought to be a prognostic factor: refractory and resistant patients vs. sensitive patients. The IDMC will be asked to review outcomes in patients with refractory and resistant disease. If appropriate a protocol amendment would be considered, allowing a switch to an alternative regimen for these patients.

Whether the disease is **locally advanced** or **metastatic** will also be controlled for at randomisation. This has been identified as a prognostic factor in ABC-02 (in press). In addition to the 3 factors

controlled for at randomisation other relevant factors may be adjusted for in the primary outcome analysis – this will all be detailed in a Statistical Analysis Plan (SAP) which will be written and agreed prior to database locking and analysis.

2.5.-Summary

Active Symptom Control (ASC) is already the standard of care following progression on first-line treatment for ABC and represents “good medicine” with pro-active management of biliary obstruction / sepsis and other tumour-related symptoms and complications. This study will determine whether fit patients (with ECOG performance score of 0-1) with ABC benefit from the addition of chemotherapy to ASC in the second-line setting (after prior therapy with cisplatin and gemcitabine) in terms of overall survival.

3.-TRIAL OBJECTIVES AND ENDPOINTS

3.1.-Trial objectives

Primary objective: This study will determine whether good PS patients (ECOG PS 0-1) with ABC, benefit from chemotherapy in the second-line setting (after prior therapy with cisplatin and gemcitabine) in terms of overall survival.

Secondary objectives: In addition we will determine the radiological response rate (for patients treated with chemotherapy), effect on patients’ quality of life and health economics of using chemotherapy in this setting.

Exploratory objectives: Archival paraffin-embedded tissue will be obtained at study entry. In addition whole blood, serum and plasma will be obtained at baseline and at 12 weeks for all patients and on disease progression for patients on the chemotherapy arm. Analysis of these samples will be subject to a separate grant application and will build on the translational elements integral to other studies in the biliary tract portfolio (ABC-03, ABC-04 and ABC-05).

3.2.-Trial endpoints

Primary endpoint:

- Overall survival

Secondary endpoints:

- Progression-free survival. Progression will be documented according to RECIST criteria for patients in the chemotherapy arm. Clinical evidence of progression will be recorded for patients in both arms.
- Response rate (chemotherapy arm only) according to RECIST v1.1
- Toxicity according to CTCAE v.4.03
- Quality of life (QLQ-C30 – validated and QoL Bil - exploratory)
- Costs of health and social care (collected from case note review and a patient diary for the study.).
- Health status (Euroqol) completed by patients at baseline and scheduled follow up assessment.
- Quality adjusted life years (QALYs) estimated from Euroqol and survival using published utility tariffs

4.-TRIAL DESIGN

4.1.-Overall design

This is a randomised (1:1) phase III, multi-centre, controlled, open-label trial of 162 patients with advanced (locally advanced or metastatic) biliary tract cancer with evidence of disease progression after prior cisplatin /gemcitabine chemotherapy treatment.

Eligible patients will be randomised (1:1) to receive either Active Symptom Control (ASC; the “standard” arm) or ASC with mFOLFOX chemotherapy (the “experimental” arm). At randomisation 3 factors will be controlled for: response to first line treatment, serum albumin level and locally advanced vs. metastatic disease.

Patients allocated to receive chemotherapy will be seen for treatment every 2 weeks. Chemotherapy will continue (in the absence of disease progression, intolerable toxicity or patient choice to withdraw) to a maximum of 12 cycles (6 months). Patients in the experimental arm will undergo tumour evaluation by CT scan (and optional MR) after 12 weeks and at the end of chemotherapy treatment (6 months, or earlier where treatment is discontinued). Patients who have not progressed at the end of chemotherapy treatment should have repeated CT scans every 12 weeks until progression.

Quality of life assessments will be performed at baseline and 12-weekly thereafter. Endpoints for the cost effectiveness analysis include costs, QALYs and the incremental cost per QALY gained by ASC with oxaliplatin/5-FU chemotherapy. Service use and health status data will be collected at baseline and every 12 weeks to estimate overall costs and QALYs.. In addition, archival tissue and prospective blood samples will be collected for translational research.

The end of the trial will be twelve months after enrolment of the final patient or once all patients have died, whichever comes first.

Study Schema:

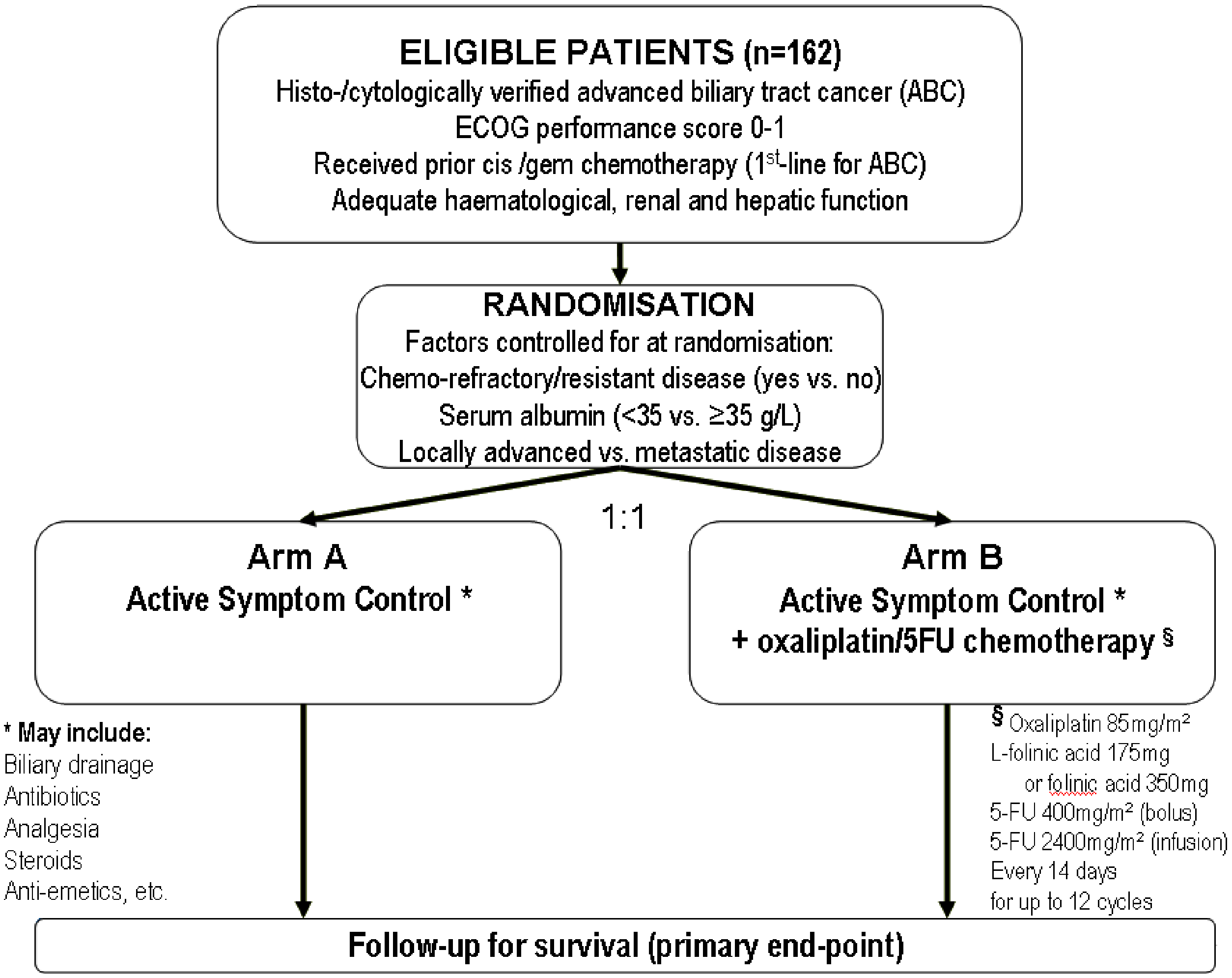

4.2.-Selection of trial participants

There will be no exceptions to the eligibility requirements at the time of randomisation. Queries in relation to the eligibility criteria must be addressed prior to randomisation. Patients are eligible for the trial if all the inclusion criteria are met and none of the exclusion criteria applies.

4.2.1.-<u>Inclusion criteria</u>

1. Histologically / cytologically verified, non-resectable or recurrent / metastatic cholangiocarcinoma, gallbladder or ampullary carcinoma.

2. Patients must have failed no more than one prior course of chemotherapy (gemcitabine* and cisplatin) with clear evidence of disease progression.
   * Patients receiving Acelarin within the ABC-08 study may be included.
3. ECOG performance status 0-1 (*Appendix 1*).
4. Age ≥18 years and life expectancy >3 months.
5. Adequate renal function:
   - serum urea and serum creatinine < 1.5 times upper limit of normal (ULN)
   - creatinine clearance ≥ 30ml/min. Creatinine clearance to be estimated using the Cockroft-Gault formula (*Appendix 2*, Wright formula may be used in place of Cockroft-Gault if this is usual local practice). For patients with estimated creatinine clearance <30 ml/min GFR may be assessed using either Cr51-EDTA or 99mTc-DTPA clearance method to confirm if GFR is ≥ 30 mL/min.
6. Adequate haematological function: Hb ≥ 100g/l, WBC ≥ 3.0 x 10^9/L, ANC ≥ 1.5 x 10^9/L, platelet count ≥ 100 x 10^9/l.
7. Adequate liver function: total bilirubin < 60 µmol/L and ALP, along with AST and/or ALT ≤ 5 x ULN
8. Adequate biliary drainage, with no evidence of ongoing infection (patients on maintenance antibiotics are eligible when acute sepsis has resolved).
9. Women of child bearing age must have a negative pregnancy test prior to study entry and be using an adequate contraception method. This must be continued for 4 months after completion of chemotherapy, unless child bearing potential has been terminated by surgery/radical radiotherapy
10. Men must be willing to use an adequate method of contraception during chemotherapy and until 6 months after chemotherapy
11. Patients must have given written informed consent
12. All patients must be randomised and sites must ensure that patients allocated chemotherapy (ARM B only) start treatment within 6 weeks of radiological progression

4.2.2.-Exclusion criteria

1. Incomplete recovery from previous therapy or unresolved biliary tree obstruction (includes ongoing neuropathy of grade >1 from cisplatin)
2. Any evidence of severe or uncontrolled systemic diseases which, in the view of the investigator, makes it undesirable for the patient to participate in the trial
3. Evidence of significant clinical disorder or laboratory finding which, in the opinion of the investigator makes it undesirable for the patient to participate in the trial

4. Any patient with a medical or psychiatric condition that impairs their ability to give informed consent
5. Any other serious uncontrolled medical conditions
6. Clinical evidence of metastatic disease to brain
7. Any pregnant or lactating woman
8. Clinically significant cardiovascular disease. [i.e. active; or <12 months since e.g. cerebrovascular accident, myocardial infarction, unstable angina, New York Heart Association (NYHA – *Appendix 3*) grade II or greater congestive heart failure, serious cardiac arrhythmia requiring medication, uncontrolled hypertension*].
9. Patients must not have a history of other invasive malignant diseases within the last 5 years (other than adequately treated non-melanotic skin cancer or in-situ carcinoma of the uterine cervix)

* Hypertension grading of ≥ 3 is an exclusion criteria (CTCAE v4.03). However, patients who have controlled hypertension with medication and/or diet may be included at the investigator's discretion. (This should be noted in the medical history section of the CRF). .

4.2.3.-Concomitant medication

Patients may receive all concomitant therapy deemed to provide adequate supportive care at the investigator's discretion. However, the use of experimental drugs is not permitted until at least 28 days after the completion of chemotherapy. Patients in either arm of the trial may participate in relevant phase I trials (if eligible) provided that this is at least 28 days after last chemotherapy treatment for patients in the chemotherapy arm. All medications or other further active treatments taken by the patient during the trial (including those initiated prior to the start of the trial) must be recorded in the patient's clinical notes and either the 'concomitant medications and procedures log' or the 'further active treatments log' as appropriate.

Premedication for chemotherapy treatment is detailed in *Section 8.2.1*.

Interactions between the investigational chemotherapy and other drugs are detailed in *Section 8.4*.

5.-RECRUITMENT OF TRIAL PARTICIPANTS

5.1.-Selection of centres/clinicians

A contract with the MCTU outlining specific responsibilities will need to be agreed and signed. This together with evidence of site specific assessment will be required before patient entry. The relevant local staff (data managers, research nurses etc) should be identified and have received information about the trial. Centres will be asked to supply appropriate reference ranges for laboratory assessments. Participant recruitment at a site will only be permitted when a trial has documented REC, regulatory and Local Trust R&D approvals. Centres should be able to comply with Research governance and The Medicines for Human Use (Clinical Trials) Regulations 2004.

All investigators must have experience of treating biliary tract cancers and access to a hepatobiliary MDT for diagnostic case review, as appropriate

5.2.-Identifying Participants

Potentially eligible participants will be identified by the Principal Investigators and by Sub-investigators of each site. The participants must have received first line chemotherapy (cisplatin-gemcitabine) for ABC, so they might be following up in Medical Oncology Departments from each site. When progressive disease to the first line chemotherapy is evidenced, the patients may be considered for this clinical trial.

5.3.-Consenting Participants

Patients deemed eligible for entry into the trial (after review of diagnosis, treatment history and key eligibility criteria) will be provided with a verbal and written explanation of the trial. The PI, or, where delegated by the PI, other appropriately trained site staff (GCP trained, suitably qualified and experienced), are required to provide a full explanation of the trial and all relevant treatment options to each patient prior to trial entry. During these discussions, the current approved patient information sheet for the trial should be discussed with the patient. A contact number should be given to the patient, should they wish to discuss any aspect of the trial with site staff.

After adequate time has been given (a minimum of 24 hours), all queries have been addressed and the clinical team is confident that the patient understands the trial and all requirements, patients will be consented onto the trial signing a current approved written informed consent. Written informed consent on the current approved version of the consent form for the trial must be obtained before any trial–specific procedures are conducted.

Site staff are responsible for:

- Assessing a patient's capability to give informed consent.
- Ensuring that all patients are fully informed about the trial
- Checking that the correct (current approved) version of the patient information sheet and consent form are used.
- Checking that information on the consent form is complete and legible.
- Checking that the patient has completed/initialled all relevant sections and signed and dated the form.
- Checking that an appropriate member of staff has countersigned and dated the consent form to confirm that they provided information to the patient.
- Checking that an appropriate member of staff has made dated entries in the patient's medical notes relating to the informed consent process (i.e. information given, consent signed etc.)
- Giving the patient a copy of their signed consent form, patient information sheet and patient contact card.
- Following randomisation, adding the patient's trial number to all copies of the consent form, which should be filed in the patient's medical notes and investigator site file

Four copies of the consent forms will be completed:

1. An **original, signed copy** of the patient consent form should be retained in the Investigator Site File.
2. A **copy** of the signed patient consent form should be retained in the patient notes.
3. A **copy** of the signed patient consent form should be sent for the translational research study to the MCRC Biobank when requested.
4. An **original OR copy** of the patient consent form should be given to the patient (each site's local policy/ preference may be followed regarding whether this is a 2nd original copy of the consent form or a photocopy).

The right of the patient to refuse to participate in the trial without giving reasons must be respected. All patients are free to withdraw at any time from the protocol treatment without giving reasons and without prejudicing further treatment. Patient withdrawal of consent from the trial must be explicitly documented in the source documents.

With the patient's permission, GPs will be informed of their patient's trial participation.

If new safety information results in significant changes in the risk/benefit assessment, the patient information sheet and associated consent form would be reviewed and updated if necessary. If the patient information sheet and consent form is updated with new safety information, all patients

(including those already being treated), would be informed of the new information, given a copy of the revised documents and asked to re-consent to continue in the trial.

When consenting patients it is essential that they fully understand the trial is ASC alone vs. ASC + mFOLFOX and that if allocated ASC alone no active cancer therapies are permitted (other than some phase I studies as detailed in section 4.2.3). If this is not acceptable to the potential participant they **must not** be consented to the trial.

5.4.-Screening for eligibility, pre-randomisation evaluation

Pre-treatment evaluations should be carried out as detailed below before randomisation. All patients must be randomised and sites must ensure that patients allocated chemotherapy (Arm B) start treatment within 6 weeks of radiological progression. Patient completed questionnaires and the research blood & tumour samples are not required for patients who fail screening, so should be completed following successful completion of all other screening assessments.

1. Histological or cytological confirmation of biliary tract cancer
2. Medical history, patients demographics and baseline symptoms
3. Concomitant medication
4. Height and weight
5. Vital signs: blood pressure, heart rate and rhythm**, respiratory rate, temperature
6. Physical exam
7. ECOG performance status (*Appendix 1*)
8. Full blood count and coagulation (PT, INR, APTT, APTT ratio***).
9. Biochemistry: Sodium, potassium, magnesium, urea, creatinine, phosphate, total protein, albumin, globulin (Appendix 2), adjusted calcium, LDH, CRP)
10. Liver function tests (including Bilirubin and ALP, along with AST and/or ALT and gamma-GT,)
11. Estimation of renal function (using the Cockroft-Gault formula (*Appendix 2*, Wright formula may be used in place of Cockroft-Gault if this is usual local practice)). If the calculated creatinine clearance is less than 30 mL/min, GFR may be assessed using either Cr51-EDTA or 99mTc-DTPA clearance method to confirm if GFR is ≥ 30 mL/min.)
12. Tumour markers: CEA, CA19-9 and CA125
13. Pregnancy test
14. Thorax-Abdomen-pelvis CT staging within 6 weeks before randomisation (RECIST, v1.1). Scans can be Thorax-Abdomen scan followed by a Pelvis scan to provide a 'complete' scan.
15. MR scan (liver) within 6 weeks prior to registration (optional)
16. Provision of patient diary to patient & collection of screening responses. The patient should be asked to complete a diary of health and social care use during the screening period (if this

is a short time period, patients should be asked to complete the diary retrospectively for the previous 2 weeks prior to randomisation). Further data will be collected at each subsequent treatment/ follow-up visit so the patient should be reminded to bring a completed diary to each visit.

17. Archival paraffin-embedded tissue and blood samples collection for translational research *
18. Completion of baseline quality of life and health status questionnaires (EORTC QLQ 30, QolBil and EQ5D) *

* not required for any patients who fail screening, to be completed once all other screening investigations successfully completed. Samples must not be taken from a patient who is infected with HIV or Hep C or other transmissible human disease; or from patients who are in high risk groups such as intravenous drug users.
** an ECG is not required

*** coagulation tests may be completed as per usual local practice prior to line insertion- INR is mandatory but others may be omitted if not required per local policy

5.5.-Ineligible and non-recruited participants

Ineligible and non-recruited participants will be treated according to principal investigator criteria. Best supportive care is recommended.

6.-REGISTRATION AND RANDOMISATION PROCEDURES

Trial sites will be required to keep a record of all patients approached to participate in the study, in order to monitor recruitment. Details of patients approached and the number who declined participation should be provided to MCTU (summary details only, no identifiers will be collected). Details of patients who were consented to the trial but were found ineligible/ did not proceed for any other reason will also be collected by MCTU (in anonymised form). Sites will be provided with instructions on how to provide this information to the CTU.

Any patient who consents to the trial and has been determined eligible will be centrally randomised by the MCTU. The clinical team at the research site will contact the CTU by a telephone call to a dedicated telephone number:

Randomisation line at MCTU:
**0161 306 3344**
(Monday – Friday; 9am to 5pm excluding Bank Holidays and the period between Christmas Day and New Year’s Day)

Sites should ensure that the following have been completed before randomising:

- Patient consent form must be signed
- Pre-randomisation evaluations must have been carried out at site as detailed in section 5.4
- The patient's eligibility for the trial must have been confirmed and the Investigator should have signed off the randomisation checklist.

Randomisation is completed within a bespoke computer system which is maintained by a MCTU statistician. Patients will be randomised 1:1 Arm A to Arm B. The randomisation method will be minimisation with a random element. The factors controlled for in the allocation will be:

- Response to previous chemotherapy treatment: first line chemotherapy treatment refractory/resistant (radiologically confirmed progression during first line chemotherapy treatment or time to radiologically confirmed progression since ending the last cycle of Cisplatin-Gemcitabine* ≤ three months (90 days)) vs. sensitive (time to radiologically confirmed progression since ending the last cycle of Cisplatin-Gemcitabine* > three months (90 days)).
- Serum albumin level (<35 vs. ≥35 g/l).
- Locally advanced vs. metastatic.

* Date of 'completion of last cycle of first line chemotherapy' is defined as the day 1 date of the last cycle given.

A member of MCTU staff will access the randomisation system and check with the member of the clinical team calling that the patient's eligibility for the trial has been confirmed. The MCTU staff member will then provide the caller with verbal confirmation of the treatment allocation and the unique Participant ID assigned.

An e-mail confirmation of the participant entering into the trial will be sent to the designated circulation list by MCTU. The Participant ID will be used for the purpose of participant identification and data collection during the study.

Upon randomisation, participants will be given a trial specific participant card, which will have the trial title, participant ID number, contact details of the Principal Investigator and out of hours contact details in cases of emergency.

7.-DISCONTINUATION/WITHDRAWAL OF PARTICIPANTS AND "STOPPING RULES"

7.1.-Withdrawal of patients

When consenting to the trial, patients are consenting to trial treatment, trial follow-up and data collection. Patients may be withdrawn from chemotherapy treatment prior to completion of all cycles for the following reasons:

- Progressive disease (according to RECIST 1.1)
- Clinical progression
- Intolerable toxicity/ adverse event/ intercurrent illness. Chemotherapy should be withdrawn if there is a toxicity related delay in delivering chemotherapy of more than 28 days.
- Patient decision
- Investigator decision
- For safety reasons e.g. recommendation of IDMC that trial should not continue.

If a patient is withdrawn from mFOLFOX trial treatment follow-up and data collection will continue as per protocol. For patients on survival follow-up only, sites should continue to collect the further active treatment concomitant medication logs in addition to the survival data. Sites should also continue to collect the concomitant medications and procedures log, where possible. Patients will only be withdrawn from further follow-up in the case of;

- Patient explicitly stating their wish not to contribute further data to the study
- Death
- End of study reached (12 months after enrolment of the final patient).

7.2.-Early discontinuation of trial

There are no planned formal early stopping rules for efficacy. An Independent Data Monitoring Committee (IDMC) will be appointed who will oversee patient safety and any recommendations from the IDMC (which may include early stopping) will be considered by the Trial Steering Group. We will include a formal early safety review, after the first 20 patients have been treated with chemotherapy (and completed at least 1 full cycle). This will be done by the Independent Data Monitoring Committee.

7.3.-Withdrawal from trial participation by sites

Should a site choose to close to recruitment the PI must inform MCTU in writing. Follow up as per protocol must continue for all patients recruited into the trial at that site and other responsibilities continue as per the written agreement between the trial sponsor and research site.

8.-TREATMENT DETAILS

8.1.-Treatment summary

Patients should receive treatment as detailed bellow:

ARM A: monthly clinical review and active symptom control as needed:

- Biliary drainage
- Antibiotics
- Analgesia
- Steroids
- Anti-emetics
- Other palliative treatment for symptom control
- Palliative radiotherapy (example bone metastases)
- Blood transfusion

ARM B:

- Active symptom control (as detailed in arm A) as needed
- Two weekly mFOLFOX chemotherapy using a central venous catheter for a maximum of 12 cycles:

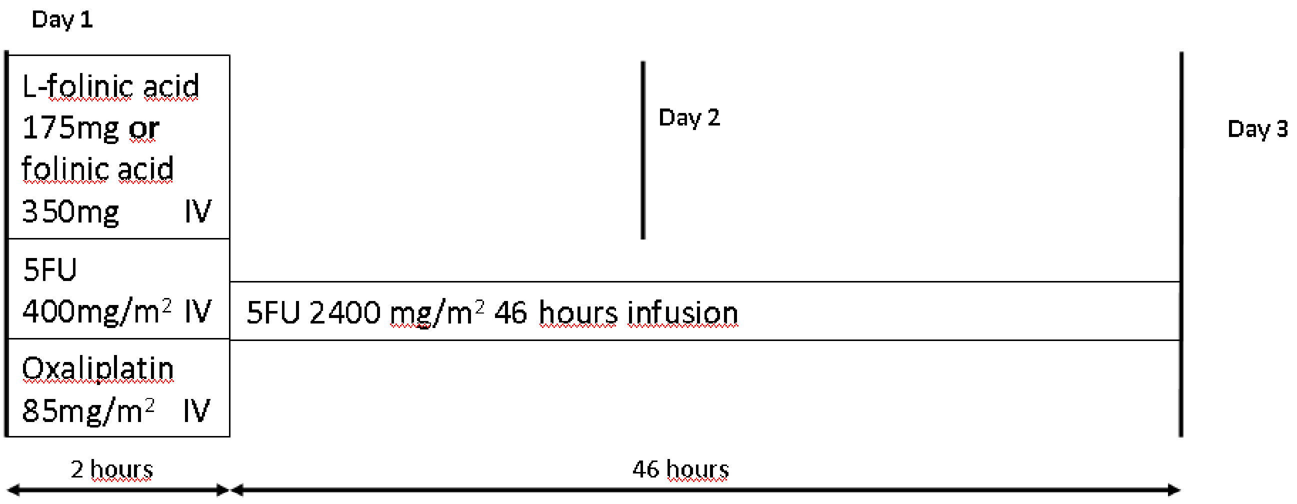

Chemotherapy treatment will be stopped in the case of:

- Disease progression (RECIST v.1.1)
- Intolerable toxicity (CTCAE v4.03)

- Patient decision
- Investigators' judgement that continued treatment is not in the best interests of the patient

8.2.-Details of trial treatment

The above chemotherapy treatments are not supplied for the trial and sites should use own shelf/ commercial stock for the trial (please refer to section 8.3).

mFOLFOX (Oxaliplatin, calcium folinate / L-folinic acid and 5FU) is currently licensed for use for cancer treatment in the UK. mFOLFOX will be sourced from routine hospital stock and its handling and management will be subject to standard procedures of the pharmacy. It will be administered to patients intravenously at the trial site. Oxaliplatin must be administered in glucose 5%, no other diluents must be used to prepare the oxaliplatin infusion for administration. Each chemotherapy treatment must be prepared and administered in a separate bag of diluent

The chemotherapy will be administered in Arm B patients until a maximum of 12 cycles, disease progression, intolerable toxicity or patient withdrawal, whichever happens first. Each cycle will be 14 days long. The investigator may stop the chemotherapy treatment at any time if it is no longer considered to be in the best interests of the patient.

8.2.1.-mFOLFOX and suggested premedication: administration, dosage, periods

Patients will be warned of the most frequent side effects and treatment recommendations of mFOLFOX (as described in SmPC). Patients may be provided with further local information sheets about FOLFOX treatment, if this is part of usual local practice. Precautions for Oxaliplatin related neuropathy (pharyngeal dysesthesia and distal neuropathy) and central line infusion chemotherapy will be explained. The most important adverse events and recommendations for patients/investigators are summarized in *Appendix 4*.

The chemotherapy administration and antiemetic premedication will follow the local protocol from each site. Suggested chemotherapy administration and antiemetic premedication, would be as summarized in *Appendix 5*. Changes in premedication or infusion time will be performed according to local rules if needed. Central line is needed for mFOLFOX administration, following each site's local procedure.

Oxaliplatin should be given in 250-500 ml of 5% glucose over 2 hours. Please refer to current SmPC for instructions regarding the preparation, stability and final concentration of oxaliplatin infusion. Dose banding is permitted.

5-FU and calcium folinate / L-folinic acid should be reconstituted according to local protocols. Dose banding of 5-FU is permitted. The dose and administration of calcium folinate/ L-folinic acid could be changed according to usual local practice.

Any brand and salt form of calcium folinate / L-folinic acid may be used.

At the Investigator's discretion, mFOLFOX patients **> 70 years** of age can be commenced on 5-fluorouracil IV continuous infusion and 5-fluorouracil IV bolus at 80% of the starting dose, if clinically indicated.

Body surface area will be capped at $2.2m^2$ for this study. Body surface area will be calculated using the DuBois formula (*Appendix 2*). BSA should be recalculated in case of weight variation > 5%.

Creatinine clearance will be checked at baseline (*Appendix 2*). Dose adjustment will be as follows:

- CrCl ≥60 ml/min full dose;
- CrCl 30-60 ml/min oxaliplatin dose reduced to 65 mg/m^2. (This is an optional dose reduction and may be omitted if not usual local practice).
- CrCl <30 ml/min at baseline is an exclusion criteria for this trial. If CrCl is <30 ml/min later in the trial no oxaliplatin should be administered (5FU and trial assessments may continue).

Contraception: Patients in the chemotherapy arm must be advised on the use of effective contraception. Male patients who are treated with oxaliplatin are advised not to conceive a child during and until 6 months after the end of oxaliplatin therapy. Women of childbearing potential must continue effective methods of contraception until 4 months after completion of chemotherapy.

8.2.2.-Overdose

Hospital pharmacy departments will be responsible for the preparation of trial drug infusions and for drug accountability. No overdose is expected, however in the event that a patient is given more than the protocol defined dose of any of the trial medications the research site should inform MCTU as soon as possible. The research site should also refer to the guidance on overdose available in the summary of product characteristics for the relevant drug/ drugs.

8.2.3.-Dose Changes and Modification of trial treatment

All dose changes and modifications will be collected in the clinical notes. The reason for dose adjustment and cycle delay will be specified. Expected toxicities are detailed in *Appendix 4*.

The following general guidance should be followed for management of adverse events and dose reductions.

• Body surface area will be capped at 2.2m^2 for this study. BSA should be recalculated in case of weight variation > 5%.

• At the Investigator's discretion, mFOLFOX patients > 70 years of age can be commenced on 5-fluorouracil IV continuous infusion and 5-fluorouracil IV bolus at 80% of the starting dose, if clinically indicated.

• Treat each of the adverse events with maximum supportive care (including withholding administration of the agent suspected of causing the adverse event where required).

• If the symptoms promptly resolve with supportive care, consideration should be given to continuing the same dose of trial medications along with appropriate continuing supportive care. If medically appropriate, dose reductions are permitted (see below).

• All dose modifications should be documented with clear reasoning and documentation of the approach taken in the case report forms.

• Dose modifications should be made to the drug(s) most likely responsible for the adverse event(s) necessitating the dose reduction.

8.2.3.1.-Dose adjustment and criteria for deferring subsequent courses

The mFOLFOX regimen is usually well tolerated. Initiation of subsequent treatment cycles will be dependent upon the full blood count and clinical assessment performed prior to treatment on day 1 every 14 days. Toxicities will be classified according to CTCAE v4.03.

Dose modifications should be made to the drug(s) most likely responsible for the adverse event necessitating the dose reduction. Wherever possible, oxaliplatin should be dose-reduced (as per Investigator discretion) rather than discontinued and can be given over a longer period of time if it is the hyperacute neurotoxicity which is particularly a problem. In the situation where oxaliplatin is discontinued due to toxicity, treatment can continue with 5-FU and folinic acid/ L-folinic acid alone if deemed appropriate by the local Investigator. In this case, the dose per m^2 of the single agent 5- FU

can be increased as per local practice at the discretion of the Investigator. Patients will still be considered to be on ABC-06 protocol treatment and will be followed-up as per protocol.

Once the dose of a specific protocol treatment has been reduced it must not be re-escalated. No more than two dose reductions will be allowed for each drug. Suggested dose adjustments are indicated in *Appendix 6*. When further dose reductions are needed, the administration of this drug will be stopped. When a treatment delay is needed because of toxicity, the patient will be evaluated weekly and the drug will not be restarted until recovery to ≤ grade 1. No more than 4 weeks of delay is allowed, if there is more than 28 days of treatment delay the patient will receive no further protocol-mandated treatment. Patients withdrawn from protocol treatment will still be followed up as per protocol and details of further treatment given outside the trial will be recorded.

8.2.3.1.1.-Dose adjustment for haematological toxicity

The following dose modifications are provided as a guideline in the event of haematological toxicity, however Investigators are permitted to follow their local practice for the management of haematological toxicity, with all dose modifications fully documented in the patient's medical record and CRFs. For decreased haemoglobin levels, bloods transfusions or employment of erythropoietin is allowed following local practice at each site. G-CSF management of neutropaenia will be at the discretion of the local Investigator.

- Neutrophils ≥1.5 x 10^9/l and Platelets ≥75 x10^9/l: treat with full dose on time
- Neutrophils <1.5 x 10^9/l and/or Platelets <75 x10^9/l: delay treatment until neutrophils and platelets are above these limits
- If > 1 delay, or 1 delay of ≥ 2 weeks occurs, maintain oxaliplatin and infusional 5-FU doses, but omit bolus 5-FU and continue without bolus 5-FU for subsequent doses.
- If further delay(s) for myelotoxicity occur despite omitting bolus 5-FU, reduce the oxaliplatin and infusional 5-FU doses by 20%. Further dose reductions can be made at the discretion of the local Investigator.
- If on day 1 of the second cycle:
    - Neutrophils <1.0: Reduce doses for subsequent courses by 20% (and omit bolus 5-FU from mFOLFOX regimen)
    - Neutrophils <0.5: Investigators may at their discretion reduce doses for subsequent courses by 50% (and omit bolus 5-FU from mFOLFOX regimen). A 20% dose reduction with G-CSF would be an acceptable alternative.

8.2.3.1.2.-Dose adjustment for non-haematological toxicity

Diarrhoea

Diarrhoea with these regimens is a consequence of the 5FU. Therefore reduction in this drug is the most important adjustment to make. The table below gives recommendations but dose adjustments according to local protocol may be followed as long as the dose given is carefully annotated in the CRF.

Toxicity	Treatment	Dose adjustment
**Diarrhoea**		
Grade 1	Continue chemotherapy	Not needed
Grade 2	Start medical treatment for diarrhoea (loperamide recommended, other treatments can be employed following the rules of each site). Delay the treatment until grade ≤1 is recovered. If not recovered in 28 days, mFOLFOX will be permanently stopped.	If first occurrence, no dose reduction needed. If second occurrence, restart chemotherapy with 20% dose reduction (both bolus and infusion of 5FU). If third occurrence, restart at 50% dose (both bolus and infusion of 5FU).
Grade 3		If first occurrence, restart chemotherapy with 20% dose reduction (both bolus and infusion of 5FU). If second occurrence, restart at 50% dose (both bolus and infusion of 5FU).If third occurrence, consider the discontinuation of all chemotherapy.
Grade 4		Consider the discontinuation of all chemotherapy or, if rapid recovery, restart at 50% dose (both bolus and infusion of 5FU).

If dose reduction of 5-FU does not result in improved tolerance then consideration should be given to reduction of oxaliplatin dose to 80% on mFOLFOX regimen.

Neurosensory Toxicity

Neurosensory toxicity with these regimens is felt to be a consequence of the oxaliplatin. Therefore reduction in this drug is the most important adjustment to make. Acute dysesthesia of the larynx may

be mitigated by slowing the rate of infusion of oxaliplatin. If repeat events of neurosensory toxicity occur; local practice should be followed with the management of the toxicities being fully documented in the patient's medical records.

Peripheral neuropathy		
Grade 1 or grade 2 (persisting less than 7 days)	Continue chemotherapy with full dose of oxaliplatin.	
Grade 2 (persisting more than 7 days)	Reduce oxaliplatin dose by 20%.	
Grade 3 and 4	Stop oxaliplatin.	
Pharyngeal dyshestesia	Treatment	Dose adjustment
Any grade, during/after (until 2 hours) the infusion of oxaliplatin	Benzodiazepines and steroids are allowed, stop the infusion and restart when patient is recovered, prolonging the infusion time.	Prolong the infusion time of oxaliplatin on the next cycle, premedication could also be increased (magnesium and calcium infusions) according to local practice. Dose reduction not needed.

Renal impairment

As with other platinum drugs, creatinine clearance should be checked (*Appendix 2*). Dose adjustment will be as follows:

- CrCl ≥60 ml/min full dose;
- CrCl 30-60 ml/min oxaliplatin dose reduced to 65 mg/m^2. (This is an optional dose reduction and may be omitted if not usual local practice).
- ClCr<30ml/min no oxaliplatin will be administered (5FU and trial assessments may continue)

Respiratory Toxicity

As with other platinum drugs, rare cases of acute interstitial lung disease or lung fibrosis have been reported with oxaliplatin. In the case of unexplained respiratory symptoms or signs, oxaliplatin should be discontinued until further pulmonary investigations exclude an interstitial lung disease.

Stomatitis

Routine mouth care is recommended. If mouth ulcers occur despite this, dose reduce 5-FU as per table for diarrhoea.

Allergic Reactions to Oxaliplatin

The occasional patient (approximately 0.5%) develops acute hypersensitivity to oxaliplatin, usually after more than 6 cycles have been administered. During drug administration, the patient may develop rash, fever, swollen mouth or tongue, hypo- or hypertension and other signs/symptoms of hypersensitivity. This rarely develops to full-blown anaphylaxis, even with repeated treatment.

If severe hypersensitivity occurs, discontinue the infusion and treat with IV corticosteroid and antihistamine. After full recovery, the patient may continue with calcium folinate / L-folinic acid and 5-FU alone.

Allergic reaction to oxaliplatin	Treatment	Dose adjustment
Grade 1 or 2	Increase premedication, following local practice.	Dose reduction not needed.
Grade 3 or 4	Stop oxaliplatin.	Stop oxaliplatin.

Other toxicities

Other toxicities		
Grade 3 and 4	Delay the treatment until grade ≤1. If not recovered in 28 days,	Reduce by one level the dose of the drug related to the

	mFOLFOX will be permanently stopped.	toxicity (dose levels are defined in appendix 6). According to local practice, the 5FU bolus can be omitted instead of reducing the dose of the infusion.
Exceptions: alopecia, anorexia, lethargy, nausea and vomiting, constipation	No dose reduction needed if well controlled with medical treatment.	

8.2.3.1.3.-Dose modifications in case of biliary tract obstruction during treatment

In the event of the development of obstructive jaundice due to biliary tract obstruction, appropriate measures will be undertaken to diagnose (e.g. by ultrasound and/or CT scan) and relieve the obstruction (e.g. by ERCP/PTC +/- stent insertion/drainage). Protocol treatment will be deferred until the liver function tests have resolved to the protocol eligibility levels (i.e. total bilirubin < 60 μmol/L and ALT, AST & alkaline phosphatase (5 x ULN). Protocol treatment may then resume at the start of the next treatment cycle. If there is a toxicity related delay in chemotherapy of more than 28 days the patient should be withdrawn from chemotherapy treatment.

Biliary tract obstruction by itself shall not constitute evidence of disease progression. CT or MRI/MRCP imaging will be performed at the planned time points (i.e. after 3 and 6 months of treatment). However, if there is radiological evidence of disease progression compared to baseline during the investigation of obstructive jaundice, the patient shall be withdrawn from mFOLFOX treatment.

8.3.-Management and handling of investigational medical product (IMP)

The listed IMPs for this study are oxaliplatin, and 5-fluorouracil. Calcium folinate / L-folinic acid can be classified as a NIMP. All IMPs/NIMPs for use in this trial must be taken from existing pharmacy shelf/ commercial stock. There is no provision for funding, reimbursement or discounted stock. IMPs/NIMPs should be stored under the correct temperature and storage conditions as per the SmPC. All products used in this study are licensed medications and will not be labelled specifically for the study, additional labelling containing dispensing information should be undertaken as per local practice. IMP accountability logs will be provided for use; these must be maintained for the duration of the study and must be kept in the study pharmacy file, these will be monitored on site. At the end of the study all accountability logs should be retained with the CRFs for patients at that site as a record of drug

accountability. IMP/NIMP destruction, if necessary, should be undertaken in line with local policies and procedures.

8.4.-Non-study treatment

8.4.1.-Concomitant treatment

Patients may receive all concomitant therapy deemed to provide adequate supportive care at the investigator's discretion. All such medications or other further active treatments taken by the patient during the study (including those initiated prior to the start of the study) will be recorded in the patient's clinical notes and either the 'concomitant medications & procedure log' or the 'further active treatments log' as appropriate. Medications given by site as standard as part of mFOLFOX do not need to be recorded on the concomitant medication log (e.g. dexamethasone, ondansetron).

It is acceptable for calcium and magnesium supplements to be given concurrently with trial treatment and these are sometimes prescribed in an attempt to decrease acute neurotoxicity. If supplements are given during the course of chemotherapy treatment this should be recorded on the Treatment CRF.

The use of experimental drugs is not permitted until at least 28 days after the completion of chemotherapy. Patients in either arm of the trial may participate in relevant phase I trials (if eligible) provided that this is at least 28 days after last chemotherapy treatment for patients in the chemotherapy arm.

8.4.2.-Interaction with Other Drugs

Interactions between the study drugs and any concomitant medications should be checked with the SmPC in accordance with local practice. The drugs listed below may interact with some of the IMPs given as part of this trial. We do not consider these drugs to be a recommended standard treatment for this patient group but acknowledge that there may be instances where patients receive these treatments concomitantly. If this is the case, then caution should be taken as indicated below. This list is not intended to be comprehensive and local practice and guidelines should be followed for management of all other drugs.

Warfarin: We recommend that patients do not receive concomitant chemotherapy and warfarin as the disturbance in warfarin metabolism during chemotherapy treatment is unpredictable and difficult to manage. If the Local Investigator feels there is no alternative to giving warfarin concurrently, then

these patients must have their anticoagulant response (INR or prothrombin time) monitored frequently in order to adjust the anticoagulant dose accordingly.

Allopurinol: Interactions with allopurinol have been observed for 5-FU; with possible decreased efficacy of 5-FU. Concomitant use of allopurinol with 5FU should be avoided.

9.-TRIAL ASSESSMENTS

For a summary of scheduled assessments, please see Schedule of Events (*Appendix 7*)

9.1.-Screening assessments

Screening assessments are defined in Section 5.4. Pre-treatment evaluations should be carried out before randomisation.

9.2.-Imaging assessments

CT imaging should be performed according to usual local practice e.g. may include dual phase liver imaging (arterial and portal phase) and will be reported in accordance with RECIST v1.1.

An optional MR scan may be performed in addition to CT, according to the patients liver disease and usual local practice. MR imaging procedure should follow usual local practice, contrast enhanced MR imaging is optional.

See sections 5.4, 9.3 and 9.4 for imaging timepoints.

9.3.-Assessments to be performed in Arm A

Patients randomised to the non-chemotherapy arm should have the following evaluations during the monthly follow-up.

9.3.1.-Monthly visit:

1. Vital signs: blood pressure, heart rate and rhythm (ECG not required), respiratory rate, temperature
2. Physical exam
3. ECOG performance status (*Appendix 1*)
4. Full blood count

5. Biochemistry: Sodium, potassium, magnesium, urea, creatinine, phosphate, total protein, albumin, globulin (Appendix 2), adjusted calcium, LDH, CRP)
6. Liver function tests (including bilirubin and ALP, along with AST and/or ALT and gamma-GT,)
7. Tumour markers: CEA, CA19-9 and CA125
8. Active symptom control: biliary drainage, antibiotics, analgesia, steroids, anti-emetics and any other palliative treatment for symptom control (example: palliative radiotherapy, blood transfusion etc).
9. Concomitant medication record
10. Symptom follow-up
11. This visit will be performed from randomisation until end of follow-up (twelve months after randomisation of the last patient) or death, whichever happens first. It may be by telephone if patient's condition precludes hospital visit. In the case of visits performed by telephone the primary aim will be to confirm survival status and any ongoing further active treatment as further assessments will not be possible.
12. Collection of patient diary (costs of health and social care)

If a monthly visit is missed it should be rescheduled as soon as possible. Future visits should then be planned 4 weekly from the date of the rescheduled visit.

9.3.2.-Every three months:

1. All the above in Section 9.3.1
2. Blood sample collection for translational research- at 12 week visit only
3. Completion of quality of life and health status questionnaires (EORTC QLQ 30, QolBil and EQ5D).

9.4.-Assessments to be performed in Arm B

Patients randomized to mFOLFOX second line treatment will have the following assessments:

9.4.1.-During the chemotherapy treatment, *patients will attend clinic on day 1 of every 14-day cycle for up to 12 cycles. The number/frequency of visits can change according to cycle deferrals defined in Section 8.2.3.*

For **all cycles** of chemotherapy these investigations and assessments may be carried out up to 3 working days before treatment:

1. Weight
2. Vital signs: blood pressure, heart rate and rhythm (ECG not required), respiratory rate, temperature
3. Physical exam
4. ECOG performance status (*Appendix 1*)
5. Full blood count
6. Biochemistry: Sodium, potassium, magnesium, urea, creatinine, phosphate, total protein, albumin, globulin (Appendix 2), adjusted calcium, LDH, CRP
7. Liver function tests (including bilirubin and ALP, along with AST and/or ALT and gamma-GT,)
8. Tumour markers: CEA, CA19-9 and CA125
9. Estimation of renal function (using the Cockroft-Gault formula (*Appendix 2*), Wright formula may be used in place of Cockroft-Gault if this is usual local practice)
10. mFOLFOX chemotherapy according to Section 8.
11. Active symptom control: biliary drainage, antibiotics, analgesia, steroids, anti-emetics and any other palliative treatment for symptom control (example: palliative radiotherapy, blood transfusion etc).
12. Concomitant medication record
13. Follow-up any adverse event, toxicities or new symptoms until resolution or patient's death. The relationship with the treatment or the disease should be recorded.
14. Collection of patient diary (costs of health and social care)

Week 12 of chemotherapy (*N.B. these assessments should be completed **after cycle 6**, they may be conducted later than week 12 if there have been treatment delays):*

1. All those needed for day 1 of chemotherapy (except mFOLFOX chemotherapy)
2. Thorax-Abdomen-pelvis CT (RECIST v1.1). Scans can be Thorax-Abdomen scan followed by a Pelvic scan to provide a 'complete' scan.
3. MR scan (liver) (optional)
4. Blood samples collection for translational research
5. Completion quality of life and health status questionnaires (EORTC QLQ 30, QolBil and EQ5D).

9.4.2.-End of treatment to be carried out within 30 days of the last treatment dose *(including termination on disease progression or early termination for other reasons), patients will have the following assessments:*

1. Weight
2. Vital signs: blood pressure, heart rate and rhythm (ECG not required), respiratory rate, temperature
3. Physical exam
4. ECOG performance status (*Appendix 1*)
5. Full blood count
6. Biochemistry: Sodium, potassium, magnesium, urea, creatinine, phosphate, total protein, albumin, globulin (Appendix 2), adjusted calcium, LDH, CRP)
7. Liver function tests (including bilirubin and ALP, along with AST and/or ALT and gamma-GT,)
8. Tumour markers: CEA, CA19-9 and CA125
9. Thorax-Abdomen-pelvis CT (RECIST v1.1) Scans can be Thorax-Abdomen scan followed by a Pelvic scan to provide a 'complete' scan.
10. MR scan (liver) (optional)
11. Active symptom control: biliary drainage, antibiotics, analgesia, steroids, anti-emetics and any other palliative treatment for symptom control (example: palliative radiotherapy, blood transfusion etc).
12. Concomitant medication record
13. Follow-up any adverse event, toxicities or new symptoms until resolution or patient's death. The relation with the treatment or the disease might be specified.
14. Collection of patient diary (costs of health and social care)

9.4.3.-During the follow up, *after chemotherapy has finished/stopped, patients will have:*

A **monthly visit** with:

1. Vital signs: blood pressure, heart rate and rhythm (ECG not required), respiratory rate, temperature
2. Physical exam
3. ECOG performance status (*Appendix 1*)
4. Full blood count
5. Biochemistry: Sodium, potassium, magnesium, urea, creatinine, phosphate, total protein, albumin, globulin (Appendix 2), adjusted calcium, LDH, CRP)
6. Liver function tests (including bilirubin and ALP, along with AST and/or ALT and gamma-GT,)
7. Tumour markers: CEA, CA19-9 and CA125

8. Active symptom control: biliary drainage, antibiotics, analgesia, steroids, anti-emetics and any other palliative treatment for symptom control (example: palliative radiotherapy, blood transfusion etc).
9. Concomitant medication record
10. Follow-up any adverse event, toxicities or new symptoms until resolution or patient's death. The relation with the treatment or the disease might be specified.
11. When progression is shown in follow-up CT scan: blood samples collection for translational research.
12. Collection of patient diary (costs of health and social care)

This visit may be by telephone if patient's condition precludes hospital visit. In the case of visits performed by telephone the primary aim will be to confirm survival status and any ongoing further active treatment as further assessments will not be possible.

If a monthly visit is missed it should be rescheduled as soon as possible. Future visits should then be planned 4 weekly from the date of the rescheduled visit.

Every three months:

1. All the above
2. Completion of quality of life and health status questionnaires (EORTC QLQ 30, QolBil and EQ5D).

10.-TRIAL CLOSURE

The end of the trial will be twelve months after randomisation of the last patient included, or after the death of all the patients, whichever happens first.

Patients in Arm A will be followed up monthly from randomisation until death or the end of the trial, whichever happens first.

Patients in Arm B, will start two weekly mFOLFOX chemotherapy treatment. According to response and toxicity, after 6 cycles of chemotherapy, up to another six cycles could be administered (total of 12 cycles, 6 months of treatment). When chemotherapy is finished/stopped, monthly follow up will start. This follow-up will continue until death or the end of the trial, whichever happens first..

Patients are able to withdraw from the trial as detailed in Section 7.

Investigators are able to stop the trial due to safety concerns and sites can also be closed (Section 7).

After end of trial chemotherapy due to progression or toxicity, treatment will be at the treating clinician's discretion.

11.-STATISTICS AND DATA ANALYSIS

11.1.-Sample size calculation

This is a phase III, randomised, multi-centre, open-label study. For the sample size calculation, the following assumptions have been made:

- The median overall survival for Active Symptom Control: 4 months (based on ABC-02 results)
- The one-year survival from second-line randomisation: approximately 10% (as per Kaplan-Meier curve from ABC-02 at 24 months, given that the first year is taken up with 1st-line effect)
- The baseline survival is approximately Exponential (ë=0.1733)
- Patients will be randomised 1:1 to each arm
- Randomisation will be by minimisation with a random element. Factors controlled for at randomisation will be chemo-refractory disease (yes vs. no), serum albumin (<35 g/L vs. ≥35 g/L) and locally advanced vs. metastatic disease.
- Minimal loss to follow-up is envisaged (<3%)

Calculations:

- A hazard ratio of 0.63 is the alternative for which the power is specified (equivalent here to an increase in median survival from 4 to 6.4 months)
- Power: 80%, alpha: 5%, two-tailed log-rank test
- 148 events needed (deaths) by the time of analysis
- A total of 162 patients are required (approximately 81 on each arm). If the assumptions regarding the baseline and alternative survival functions hold, recruitment is as specified below and there is 12-months follow-up on the last case we would expect to observe around 152 events.

Allowing for the minimal loss to follow-up envisaged the required number of events should be delivered.

11.2.-The recruitment rate

The UK has an established network of investigators for biliary tract cancer studies under the auspices of the NCRN; high-recruiting centres into the ABC-02 and ABC-03 studies (both first-line chemotherapy) will be opened to accrual:

The target recruitment time is 2.5 years, with 12 months follow-up

We estimate that we are able to recruit 6 patients/month (steady state) from 20 centres; allowing for a 6-month lead-in (2 patients / month for months 1-3, and 4 patients per month for months 4-6) = 162 patients.

The proposed recruitment means that that recruitment at 6 months is projected to be 18 patients; 54 patients at 12 months and 90 patients at 18 months. Recruitment will be reviewed by the Trial Management Group every 6 months to ensure that recruitment is adhering to this plan with the formal milestone at 18 months. If required, options to increase accrual will be reviewed; particularly opening additional centres to the 20 initial centres planned (note: ABC-02 was opened in 37 centres across the UK and not all centres wishing to participate in ABC-06 have been accommodated); other options (including international collaboration) will also be considered.

The rate of loss of follow up is expected to be very low given the poor prognosis of this patient group (overall survival less than 6 months) and as active symptom control is the standard of care for these patients out with the context of a clinical trial.

11.3.-Discontinuation rules

There are no planned formal early stopping rules for efficacy. An early safety review after the first 20 patients have been treated with chemotherapy (completing at least 1 full cycle) will be included (See Section 7.2). This will be done by the Independent Data Monitoring Committee.

11.4.-Primary analysis

The primary analysis will be an 'Intention-To-Treat' cox regression for the overall survival outcome, adjusting for the three factors controlled for at randomisation. Other factors may also be adjusted for in the primary outcome analysis- this will all be detailed in a Statistical Analysis Plan (SAP) which will be written and agreed prior to database locking and analysis.

There are no planned sub-group or interim analyses i.e. a single analysis is planned.

11.5 Secondary analysis

Economic analysis will be completed as per section 11.6 below. Analysis of other secondary outcomes will be as defined in a detailed Statistical Analysis Plan (SAP), which will be written and agreed prior to database locking and analysis.

11.6 Economic analysis

The overall aim of the health economic analysis is to estimate the cost effectiveness of the intervention compared to ASC. The evaluation will use a within trial analysis using individual level patient data to identify the costs, health benefits and cost effectiveness of ASC plus oxaliplatin / 5-FU chemotherapy over a 12 month time horizon. The key objectives for the trial analysis are to:

- Estimate the costs of health and social care in the intervention and standard care groups and assess whether there are differences in costs between the two groups;
- Estimate the health status and quality adjusted life years (QALYs) of patients in the intervention and standard care groups and assess whether there are differences in health status and QALYs between the two groups;
- Assess whether any additional benefit of the intervention is worth the additional cost.

11.6.1 Measure of health benefit

The measure of health benefit for the primary analysis is the QALY. This will be estimated from the EQ-5D-5L and associated utility tariffs completed at baseline and end of scheduled follow up (28;29). The EQ-5D is a validated generic health status measure, used in national health surveys in the United Kingdom and in clinical trials in cancer, covering five domains (mobility, self-care, usual activity, pain/distress, and anxiety/depression). The five level version will be used in the ABC06 trial (no problems, slight problems, some problems, severe problems or unable to do activity). The QALY and the EQ-5D are the measures recommended for economic evaluations by NICE (30). The health status profiles will be converted to utility values using the published utility tariffs for the EQ-5D-5L if available. If the utility weights for the 5 level version of the EQ-5D are not available at the time of analysis, the published crosswalk methodology will be used. [http://www.euroqol.org/about-eq-5d/valuation-of-eq-5d/eq-5d-5l-value-sets.html]. This methodology mapped the 5 level to the 3 level version of the EQ-5D to derive utility values for the 3126 possible health states in the EQ-5D-5L (5 dimensions to the power of 5 levels =5^5 = 3126). There are limitations to the methodology and utility values from direct measurement and estimation will be used in preference if available.

The QALYs will be estimated as:

$$QALY = \Sigma[(U_i + U_{i+1}) /2] \times (t_{i+1} - t_i)$$

where U = utility value and t = number of days between assessments.

11.6.2 **Resource use and costs**

Direct costs will be estimated for the primary analysis. The direct costs of treatment and subsequent care will be estimated by summing the costs of each resource used as an input to provide health and social care. For each type of resource the cost will be estimated as the quantity of that resource or service used multiplied by the unit cost specific to that resource.

Data on the use of hospital inpatient and outpatient care and medication will be obtained by case note review. Data on the use of other health and social care services will be collected from a patient diary completed at baseline and during follow up. Services covered in the patient diary include:

- Whether the participant has used any hospital inpatient and outpatient services and details of the hospital(s) visited.
- Primary and community care services (type and frequency of use)
- Patient costs and expenses
- Support from family and friends (type and frequency of use)

The unit costs of NHS and social care services will be derived from national average unit cost data published in the NHS reference costs database and the annual Unit Costs of health and Social Care published by the Personal Social Services Research Unit (PSSRU), University of Kent. The unit costs of prescribed medications will be obtained from the British National Formulary (BNF). The price year will be the most recent unit costs available at the time of the analysis.

Costs will be estimated for the screening period and the 12 month period following baseline.

11.6.3 **Missing data**

The economic data will be analysed using an intent-to-treat approach and missing data will be imputed. All missing resource use data will be treated as missing at random. This is based on the assumptions that

- The level of missing data will be minimal
- The use of services other than the intervention is determined by external factors (e.g., availability and organization of services, and a patient’s living situation and accommodation) rather than management of advanced biliary tract cancer.

Multiple imputation (propensity score method) will be used to impute values for missing costs, by category of resource use. The imputation model will include assessment point and treatment allocation group as fixed covariates.

Missing health status data for patients who complete scheduled follow-up but have missing observations will be imputed by multiple imputation using the same approach as for costs. For patients with censored data due to withdrawal or loss to follow-up the assumption that the data is missing at random is less likely to hold. Missing utility values will be imputed from the mean values of those who completed scheduled follow-up or died, for each treatment allocation. Cox regression will be used to estimate the survival function and probability of survival at final assessment, using patient status (alive, dead, or withdrawn) and treatment allocation. The QALYs for this group will be estimated as:

$$QALY_C = \Sigma[(U_i + U_{i+1})/ 2][(S_i + S_{i+1})/2](t_{i+1} - t_i)$$

where U = utility value, S = probability of survival, and t = number of days between assessments.

11.6.4 Economic data analysis

The economic analysis will be conducted from the health and social care service providers and patient’s perspective. These are expected to be the key actors so approximating a societal perspective. The time horizon for the evaluation is 12 months, the total duration of scheduled follow-up in the trial.

The within-trial economic analysis will use the framework of cost effectiveness (CEA) and cost effectiveness acceptability analysis (CEAA) to explore the relative cost-effectiveness of the ASC plus chemotherapy intervention. The analysis will use individual patient level cost and health benefit data from all participants recruited and starting allocated treatment in the trial. The measure of health benefit for the primary analysis will be QALYs. Secondary analysis will explore the cost effectiveness of the intervention using the primary clinical outcome measure. The time horizon is 1 year, so costs and outcomes will not be discounted. Descriptive analysis and data manipulation will be conducted using SPSS, and the main statistical analyses and estimation of net benefit statistics and cost-effectiveness acceptability analysis will be conducted using STATA version 11.0.

The primary measure of interest for the economic analysis is the ICER, a joint measure of costs and outcomes, rather than the individual cost and outcome variables. Accordingly, no statistical tests of differences in mean costs or outcomes will be conducted. The ICER is estimated as the

$$\frac{Cost_{ASC\ plus\ chemotherapy} - Cost_{usual\ care}}{Utility_{ASC\ plus\ chemotherapy} - Utility_{usual\ care}}$$

Regression analysis will estimate the net costs and outcomes of the therapy intervention, controlling for baseline covariates. Published literature will be used to identify whether other clinical or demographic variables collected in the trial are potential determinants of costs and utility values. Correlation and ANOVA analyses of the pooled dataset will be used to identify associations between baseline variables and costs and utility values. Variables with a *P*-value of 0.05 or less will be included as covariates to estimate adjusted means for total cost per person, total utility per person and to conduct the CEAA, for the primary and all sensitivity analyses.

The estimates of incremental costs and outcomes from the regression will be bootstrapped to simulate 10,000 pairs of net cost and net outcomes of the therapy group for a cost effectiveness acceptability analysis, as recommended by NICE for health technology appraisals (30). These simulated data will be used to estimate the probability that the intervention is cost effective compared to usual care (31).

This approach revalues effects or benefits in monetary terms. However, in the UK there is no universally agreed monetary value for the types of benefit measures used in cost effectiveness analyses. An approach used in health care is to ask the question: what is the maximum amount decision makers are willing to pay to gain one unit of benefit? The simulated net utility values will be revalued using a range of maximum willingness to pay values from £1 to £30,000 to gain one unit of outcome. These reflect a range of hypothetical willingness to pay thresholds (WTPT) from decision makers being willing to pay £1 to gain a one unit increase in outcome to them being willing to pay £30,000 to gain a one unit increase in outcome. This includes the range of willingness to pay values to gain 1 QALY, implied by NICE decisions (32).

The data for the cost effectiveness acceptability curve are derived by first revaluing each of the 10,000 net outcome scores from the bootstrap simulation by a single WTPT. This is repeated for each WTPT. A net benefit statistic (NB) for each pair of simulated net costs and net outcomes for each WTPT can then be calculated as

*NB = (O * WTPT) – C, where O = net outcome score and C = net cost.*

This calculation will be repeated for each WTPT. Cost-effectiveness acceptability curves plot the proportion of bootstrapped simulations where the net benefit of an intervention is greater than zero for each WTPT (33-36).

Sensitivity analysis will be used to test the impact of assumptions and data on the ICER and results of the cost effectiveness acceptability analysis. These will include: the unit cost used to value resources, the methods used to impute missing data; the methods used to account for variable follow up of patients and the outcome measure used in the ICER.

12.-PHARMACOVIGILANCE

12.1.-Definitions

Adverse Event (AE) - An adverse event is any untoward medical occurrence in a patient or clinical investigation subject administered a pharmaceutical product or trial-specific treatment and which does not necessarily have a causal relationship with this treatment.

Note: An AE may therefore be any unfavourable and unintended sign (including abnormal findings), symptom or disease concurrently associated with the use of the trial treatment, regardless of any relation to the treatment.

Adverse Events of Medical Significance:

1.-Adverse Reaction (AR) - An adverse reaction is any untoward and unintended response in a subject to a trial treatment which is related to any dose administered to that subject. Note: Any adverse event judged by either the reporting investigator or the sponsor as having reasonable causal relationship to the trial treatment qualifies as an AR if there is evidence or argument to suggest a causal relationship.

2.-Serious Adverse Event / Reaction (SAE / SAR) - A serious adverse event or reaction in a trial subject that:

- results in death,
- is life-threatening,
- requires in-patient hospitalisation or prolongation of existing hospitalisation,
- results in persistent or significant disability / incapacity
- is a congenital anomaly / birth defect, or
- other important medical condition (medical judgement should be exercised in deciding whether an adverse event / reaction should be classified as serious in other situations. Important adverse events / reactions that are not immediately life-threatening or do not result in death or hospitalisation, but may jeopardise the subject or may require intervention to

prevent one of the other outcomes listed in the definition above, should also be considered serious).

Note: For the purpose of this definition, life-threatening refers to an event in which the trial subject was at risk at the time of an event; not an event that might have caused death if it were more severe.

Events not to be treated as SAEs: The following events do not require reporting as an SAE, but must be recorded in the relevant section(s) of the CRF

- Disease progression.
- Elective hospitalisation to simplify treatment or study procedures
- Admissions for palliative care
- Disease related deaths

3.-Suspected Unexpected Serious Adverse Reaction (SUSAR) - Any adverse reaction that is serious and unexpected (is not consistent with the reference safety information (Investigator's Brochure [IB] or Summary of Product Characteristics [SmPC]). This may include serious adverse reactions which are expected but the severity or intensity of which exceeds that expected from the IB or SmPC.

12.2.-Detecting and reporting AEs

See flow-chart in *Appendix 9*.

All adverse events that occur between informed consent and end of trial treatment must be recorded in the patient notes. Since one of the trial treatments (Active Symptom Control) will continue to be provided until death/ end of trial it is expected that all adverse events are recorded in the patient notes until death/ end of trial. Every effort should be made to maintain a complete record of adverse events. However it is recognised that full documentation of adverse events may not be possible in the case of patients no longer attending follow-up visits because they are in the end stages of the disease and receiving the appropriate hospice or home care. For patients at this stage of their disease the pattern of adverse events is expected to be similar between the 2 arms.

Where required adverse events will be reported to MCTU via completion on the trial CRF. Serious adverse events should also be reported immediately on an SAE report form, as detailed in section 12.2.1 below. As this is an established chemotherapy regimen adverse event collection on the CRF will focus on known toxicities; the following will be recorded:

- Maximum grade experienced during each chemotherapy cycle / between each assessment for a defined list of known mFOLFOX toxicities

- Any other significant adverse events experienced. Significant adverse events are considered to include:
    - o Adverse events related to the disease, chemotherapy or active symptom control
    - o Serious adverse events (as defined in section 12.1), whether or not these are related to the disease or treatment

Non serious adverse events which are not related to the disease/ chemotherapy/ active symptom control need not be reported on the CRF.

12.2.1.-SAE Processing and Reporting at the MCTU

AEs meeting the definition of a Serious Adverse Event (SAE) must be reported to the MCTU using the trial specific SAE Report Form **immediately, and within 24 hours** of observing or learning about the event. As many sections of the SAE form should be completed as possible (with further information being provided as it becomes available) and sent to the MCTU. If the Principal Investigator is available the form should be signed and dated by the PI, but if the PI is unavailable the form can be signed by a designated representative and sent to the MCTU, and then signed and dated by the PI at a later date. All SAEs must be followed-up until resolution and site investigators must provide follow-up SAE Reports if the SAE had not resolved at the time the initial report was submitted.

Centres should respond as soon as possible to requests from the CI or designated representative (via the MCTU) for further information that may be required for final assessment of the SAE.

All SAEs must be reported by emailing a completed SAE Report Form **immediately, and within 24 hours** of becoming aware of the event to the ABC-06 Trial Manager at the MCTU

Email: saereport_manctu@manchester.ac.uk

On receipt of the SAE Report Form, the CTPM will send an acknowledgement of the SAE to the relevant members of the trial team at the participating site. This acknowledgement will include an SAE reference number which should be included on all future correspondence regarding the SAE. The CTPM then passes the SAE form to the CI (or CI's designee). The CI (or designee) reviews the site PI's assessments of seriousness and causality (and expectedness where completed) and assigns their own judgment, including an assessment of expectedness in relation to the reference safety information.

12.2.2.-Detecting and reporting a SUSAR

If an SAE is suspected to be a SUSAR, regulatory time constraints apply for expedited reporting. The CTPM will then liaise with the CI (or CI's designee) to evaluate the event for seriousness, causality and expectedness to determine whether or not the case qualifies for expedited reporting.

Expectedness of the event to the trial treatment must be determined as follows:

- Expected: The event is listed as an expected adverse event in the SmPC.
- Unexpected: The event is not listed as an expected adverse event in the SmPC, or, the severity of the event is greater than that listed in the SmPC:
  - The event is life threatening or fatal (unless stated in the SmPC as expected).
  - The patient presents with an event which is considered to be moderate or severe, but only mild is listed as expected in the SmPC.

If a difference of opinion between the PI and CI/ CI's designee gives rise to an event being classified as an SAE by one clinician and a SUSAR by another, the "worst-case" assessment is assumed i.e. it is classified as a SUSAR. Similarly, the causality assessment made by the PI at site cannot be downgraded by the CI/ CI's designee as the PI is more familiar with the patient's history, clinical signs and symptoms, lab findings and other investigations. The CI/ CI's designee may, however, upgrade the PI's assessment of causality.

SAEs assessed as having a causal relationship to the study drug/ drugs and as being unexpected (SUSARs) will undergo expedited reporting to the relevant authorities by the MCTU.

If the event is evaluated as a SUSAR, the CTPM will submit a report to the MHRA, REC and sponsor within the following timeframes :

- Fatal or life-threatening SUSARs are reported to the MHRA and REC within 7 days. Additional information / follow-up reports are sent to the MHRA and REC as appropriate within an additional 8 days.
- Non-fatal or life-threatening SUSARs are reported to the MHRA and REC within 15 days.

Any SUSARs which occur will be reported to the MHRA via the eSUSAR online reporting system by the CTPM. Direct expedited reporting of SUSARs to the MHRA by investigators is not permitted. SUSARs will also be reported to the relevant REC by the CTPM using the National Research Ethics Service (NRES) process.

Where doubt exists whether an SAE is a SUSAR, it will only be reported to the MHRA when it has been confirmed that it has fulfilled one of the criteria meeting the definition of a SUSAR. If the SUSAR represents a significant safety concern, all investigators participating in that trial will be informed as soon as possible by the CTPM. SUSARs will be notified to sponsors and investigators in accordance with the trial agreements.

SUSARs occurring in non-UK countries (if non-UK sites used) are reported by the CTPM to the Competent / National Authority in the country of origin unless responsibility for this has been delegated to a local organisation.

SUSARs reported to the CTPM from other trials (via drug manufacturers etc) will be disseminated to the sponsor and to all sites using the same trial treatment.

12.3.-Pregnancy Reporting

If a patient becomes pregnant, the patient must be withdrawn from study drug immediately. The pregnancy of a patient, or of a male patient’s partner, must be reported to the MCTU using the Pregnancy Notification and Outcome Form immediately, and within 24 hours of the participating site becoming aware of the pregnancy. The PI must obtain consent to follow the pregnancy and liaises with the obstetrician until delivery/outcome and for the postpartum period. Parental and neonatal outcomes must be recorded (by updating the Pregnancy Notification and Outcome form) even if they are completely normal and without AEs. Abnormal outcomes for the foetus or child observed at birth or during the follow-up period (e.g. spontaneous or therapeutic abortion, stillbirth, neonatal death, congenital abnormality) are considered SAEs and are reported to the MCTU on the trial specific SAE form.

The MCTU will email all Pregnancy Reports to the sponsor within 1 business day of the MCTU becoming aware. The MCTU will submit Pregnancy Reports as per the SUSAR reporting process should the pregnancy outcome meet the definition of a SUSAR.

12.4.-New Safety Findings

If a new safety finding emerges (from sources such as IMP manufacturers, data analysis, IDMC findings), the CI will review the finding for its' impact on the subjects participating in the relevant trial(s). If there is a potential impact on trial participant's safety, the sponsor takes appropriate action in conjunction with the CTPM, CI and research team. Appropriate reporting mechanisms are followed in the event of actions being taken.

12.5.-Urgent Safety Measures

Where an urgent safety measure (to prevent immediate hazard to trial subject's health and safety) is necessary, prior authorisations from the MHRA and ethics are not required.

Where the PI takes urgent action that is not consistent with the protocol to prevent harm to a subject on a trial, the PI must immediately inform the CI and CTPM and give full details of the measures taken and the decision making process surrounding the action(s) taken. The CTPM will inform the CI, sponsor, ethics and the MHRA of these measures immediately, but no later than 3 days of the actions being taken.

An amendment is formally submitted as soon as possible by the CI and CTPM to the relevant bodies in conjunction with the sponsor.

Examples of issues requiring urgent safety measures may be:

- A single report of a SAR with an unexpected outcome (e.g. death)
- An increase in rate of occurrence of SAR which is judged to be clinically important
- A post-trial SUSAR occurring after the subject has left the trial
- A new event relating to the use or development of an IMP likely to affect the safety of subjects e.g.:
    - an SAE that could be associated with the procedures and which could lead to modification of the trial conduct
    - lack of efficacy of an IMP used for a life-threatening disease

12.6.-Periodic Safety Reports

Annual progress reports will be submitted to the REC, in accordance with their procedures.

A Development Safety Update Report (DSUR) will be submitted annually to the MHRA and REC in accordance with requirements.

These periodic reports will be prepared by MCTU with the input and review of the chief investigator and will be circulated to the sponsor and participating sites.

13.-TRIAL MANAGEMENT AND OVERSIGHT ARRANGEMENTS

13.1.-Oversight Committees

13.1.1.-Trial Management Group (TMG)

A Trial Management Group (TMG) will be established and will include those individuals responsible for the day-to-day management of the trial including the Chief Investigator, co-investigators and identified collaborators, Principal Investigators, the trial statistician and the trial manager(s) (See Page 1). Where possible, membership will include a lay/consumer representative. Notwithstanding the legal obligations of the Sponsor and Chief Investigator, the TMG have operational responsibility for the conduct of the trial including monitoring overall progress to ensure the protocol is adhered to and to take appropriate action to safeguard the patients and the quality of the trial.

The TMG will meet at least quarterly once the trial is actively recruiting. Minutes will be taken at TMG meetings and copies of the minutes will be filed in the Trial Master File. The trial manager and CI will ensure that all relevant issues and actions discussed during the meeting are followed up and resolved and details of significant issues will be made available to participating sites. Minutes of all TMG meetings are available on request.

13.1.2-Trial Steering Committee (TSC)

A Trial Steering Committee (TSC) will be established and will include an independent Chairman (not involved directly in the trial other than as a member of the TSC), not less than two other independent members, a representative from a consumer group, plus the CI, one or two Principal Investigators, the Trial Manager and statistician.

The role of the TSC is to take responsibility for the scientific integrity of the trial, the scientific validity of the trial protocol, assessment of the trial quality and conduct (to ensure that the trial is being conducted in accordance with the principles of GCP and the relevant regulations) as well as for the

scientific quality of the final study report. Decisions about the continuation or termination of the trial or substantial amendments to the protocol are usually the responsibility of the TSC.

The TSC will meet once ethics approval has been given and before the trial begins recruitment. Once the trial has started the TSC should meet at least annually to monitor the progress of the trial although there may be periods when more frequent meetings are necessary. Meetings should be organised by the CI via the CTPM. Minutes will be taken at TSC meetings and copies of the minutes will be filed in the Trial Master File. The trial manager and CI will ensure that all relevant issues and actions discussed during the meeting are followed up and resolved and details of significant issues will be made available to participating sites. Minutes of all TSC meetings are available on request.

The Committee's terms of reference, roles and responsibilities will be defined in a charter agreed by all members of the TSC.

13.1.3.-Independent Data Monitoring Committee (IDMC)

An IDMC will be instigated to review accruing trial data and to assess whether there are any safety issues that should be brought to the patient's attention, whether any safety amendments should be made or if there are any reasons the trial should not continue. The IDMC will conduct an early safety review after the first 20 patients have been treated with chemotherapy (and have completed at least 1 full cycle).

The IDMC will be independent of the investigators, funder and sponsor and will comprise of a statistician and at least 2 other experts in the disease area under investigation. The CI and TMG with the support of the MCTU will be responsible for nominating IDMC members. The Committee's terms of reference, roles and responsibilities will be defined in a charter agreed by all members of the IDMC. This charter will outline any stopping rules and the frequency of analysis and IDMC meetings during the trial. The IDMC will meet in confidence at regular intervals, as appropriate to the progress of the trial and as per the schedule agreed with IDMC members.

Reports to the IDMC will be prepared and presented by the trial statistician and CTPM prior to the IDMC meeting. The IDMC Chairman will then report their recommendations to the Chairman of the TSC or TMG and may request additional reports or information if required. This report will be submitted to the TMG and TSC, and if required, the REC and the MHRA and the CI and CTPM will ensure that all actions and recommendations are followed up.

13.2.-Trial management: MCTU

The Manchester Clinical Trials Unit (MCTU) has been delegated to manage the ABC-06 trial on behalf of the study sponsor (The Christie NHS Foundation Trust). Each trial managed through the MCTU has an assigned Clinical Trial Project Manager, Data Manager, monitor and statistician. Staff at MCTU will undertake delegated responsibilities for the management of the trial, including; study set-up, trial site set-up, progress and safety reporting, patient randomisation, data management and monitoring.

13.2.1.-Data Collection

Each research site is responsible for maintaining source data for their trial patients in their medical notes, and for transcribing this data onto a trial specific case report form (CRF). All entries on the CRF, including corrections, must be made by an authorised member of trial staff. Research site staff will also provide trial patients with copies of the relevant questionnaires for completion at each required timepoint (quality of life questionnaires, health status questionnaire and EPQ).

Research sites will submit original completed copies of the CRF and patient completed questionnaires to MCTU, keeping a copy at site with the Investigator Site File.

13.2.2.-Data Handling at MCTU

Completed CRFs and questionnaires submitted to MCTU will be entered onto a trial database by the trial data manager. The trial database will be stored on a secure server with controlled access, regular back-up of data and an audit trail of changes made to the data.

Data provided to the MCTU will be checked for errors, inconsistencies and omissions. If missing or questionable data are identified, the MCTU will request that the data be clarified. All aspects of data collection and handling throughout the life cycle of the trial will be described in trial specific documents held in the trial master file at MCTU.

Upon completion of relevant data management processes prior to any interim/ final analysis the trial data will be passed to the trial statistician for analysis.

All data handling and analysis will be conducted in line with Good Clinical Practice guidelines and the Data Protection Act 1998.

13.2.3.-*Trial monitoring*

On-site monitoring will be based on a risk-based strategy and a thorough risk assessment will be completed by the MCTU as part of the trial set-up process to ascertain the frequency and intensity of monitoring visits required (although additional monitoring may be conducted if necessary). This risk assessment and associated monitoring plan will be stored at the MCTU.

The purpose of these visits is:

- To verify that the rights and well-being of patients/participants are protected.
- To verify accuracy, completion and validity of reported trial data from the source documents.
- To evaluate the conduct of the trial within the institution with regard to compliance with the currently approved protocol, GCP and with the applicable regulatory requirements

14.-ETHICAL AND REGULATORY REQUIREMENTS

The trial will be conducted in accordance with the principles of good clinical practice (GCP).

The sponsor and MCTU will ensure that the trial protocol, patient information sheet, consent form, GP letter and submitted supporting documents have been approved by the appropriate regulatory body and research ethics committee prior to any patient recruitment.

Any agreed substantial amendments will also be submitted for ethical and regulatory approval prior to implementation. The study is being conducted under the well recognised ethical framework of a Clinical Trials Authorisation (CTA) from the MHRA. Under the CTA scheme the responsibility for the management and well being of a patient is no different from everyday clinical practice, namely the attending physician and the hospital Trust. Drug companies are, of course, liable on a no fault basis for the quality and fitness for use of their product.

It is the responsibility of the PI at each site to ensure that the trial has local R&D approval and the sponsor and MCTU will verify this, plus the presence of all other essential documentation (and potentially an initiation meeting) before giving the site the “green light” to open the trial to recruitment. The PI is also responsible for ensuring that any subsequent amendments gain the necessary approvals. For centres outside the UK (If used), before enrolling patients into the trial and subsequent amendments, the PI or designee must apply for favourable opinion within their IRB/IEC requirements.

The CI, MCTU and sponsor will ensure that the ethics committee(s) and regulatory body(ies) are notified that the trial has finished (either as expected or prematurely) within required timeframes with summary reports to be provided as required.

Contracts will be established with all relevant organisations and stored at the MCTU.

14.1.-Sponsorship and indemnity

The Christie NHS Foundation Trust will act as the sponsor for this study. Delegated responsibilities will be assigned to the MCTU to manage the trial on behalf of the sponsor and to the participating sites recruiting patients into this trial.

As the sponsor is an NHS organisation the NHS indemnity scheme will apply.

Participating sites will be liable for clinical negligence and other negligent harm to participants taking part in the study and covered by the duty of care owed to them by the sites concerned. For participating sites which are part of the NHS, the NHS indemnity scheme will also apply.

Sites outside of the United Kingdom (if used) will be responsible for arranging their own indemnity or insurance for their participation in the study, as well as for compliance with local law applicable to their participation in the study.

The manufacturer supplying IMP has accepted limited liability related to the manufacturing and original packaging of the study drug and to the losses, damages, claims or liabilities incurred by study participants based on known or unknown Adverse Events which arise out of the manufacturing and original packaging of the study drug, but not where there is any modification to the study drug (including without limitation re-packaging and blinding).

14.2.-Participant Information and Informed Consent

The patient's consent to participate in the trial should be obtained after a full explanation has been given of the treatment options, including the conventional and generally accepted methods of treatment. Patients should be given sufficient time after being given the trial patient information sheet to consider and discuss participation in the trial with friends and family. A contact number should be given to the patient should they wish to discuss any aspect of the trial. Following this, the recruiting investigator should determine that the patient is fully informed of the trial in accordance with ICH GCP guidelines.

The right of the patient to refuse to participate in the trial without giving reasons must be respected. After the patient has entered the trial, the clinician must remain free to give alternative treatment to that specified in the protocol, at any stage, if he/she feels it to be in the best interest of the patient. However, the reason for doing so should be recorded and the patient will remain within the trial for the

purpose of follow-up and data analysis. Similarly, the patient must remain free to withdraw at any time from the protocol treatment without giving reasons and without prejudicing his/her further treatment.

14.3.-Participant Confidentiality and Data Protection

Patients will be assigned a unique trial ID via the MCTU trials line which will be used throughout their participation in the trial. Any personal data recorded will be regarded as confidential, and any information which would allow individual patients to be identified will not be released into the public domain.

Investigators and trial site staff must not provide any participant-identifying data (e.g. name, address, hospital reference number) to the MCTU during the course of the trial, unless with prior approval by the Research Ethics Committee. Any participant-identifying data received by the MCTU will be redacted or destroyed, and the sender notified.

Each investigator should keep a separate Trial ID and screening log of all participants consented and screen status. The investigator must maintain this screening log and all other trial documents (including participant's written consent forms) which are to be held at the participating centres, in strictest confidence. The investigator must ensure the patients' confidentiality is maintained.

The MCTU will maintain the confidentiality of all patients and will not reproduce or disclose any information by which patients could be identified. The Investigator and trial site staff involved with this trial may not disclose or use for any purpose other than performance of the trial, any data, record, or other unpublished, confidential information disclosed to those individuals for the purpose of the trial.

Representatives of the MCTU and the regulatory authorities will be required to have access to patients notes for quality assurance purposes but patients should be reassured that their confidentiality will be respected at all times. Prior written agreement from the Sponsor or its designee must be obtained for the disclosure of any confidential information to other parties.

All Investigators and trial site staff involved with the trial must comply with the requirements of the Data Protection Act 1998 with regard to the collection, storage, processing and disclosure of personal information and will uphold the Act's core principles. The Sponsor's host institution will be the data custodian.

Patient notes and trial files at site must be kept in a secure storage area with limited access. Computers used to collate the data will have access restrictions via user names, passwords and the use of encrypted digital files and storage media.

Published results will not contain any personal data that could allow identification of individual patients.

14.4.-Direct Access to Data

By participating in the ABC-06 trial, the Principal Investigators at each centre are confirming agreement with his/her local NHS Trust to ensure that:

- Sufficient data is recorded for all participating patients to enable accurate linkage between hospital records and CRFs;
- Source data and all trial related documentation are accurate, complete, maintained and accessible for monitoring and audit visits;
- Trial-related monitoring, audits, IRB/IEC review, and regulatory inspection(s) are permitted and direct access to source data/documents is provided as required

15.-TRIAL CONDUCT

15.1.-Protocol Amendments

Any changes in research activity will be reviewed and approved by the Chief Investigator and submitted in writing to the appropriate REC, Regulatory Authority and local R&D for approval prior to enrolment into an amended protocol.

Deviations from the protocol may be taken by an investigator without prior approval from the sponsor or regulatory bodies in order to eliminate an immediate hazard to a patient. The rationale must be submitted to the MCTU and the appropriate regulatory bodies as soon as possible after the deviation.

15.2.-Protocol Deviations and Serious Breaches

The Regulations and guidance state that no deviation must be made from an approved trial protocol, unless it is an urgent safety measure taken to protect a patient from immediate harm, therefore the Christie does not allow protocol waivers for any other reason. Participating sites must contact the MCTU if a potential protocol deviation has occurred (or if an event has occurred and it is unclear whether it should be classified as a deviation) and the MCTU will advise the site what information and actions are required.

For Clinical Trials of Investigational Medicinal Products (CTIMPs), there is a legal requirement to report serious breaches of GCP or the trial protocol to the MHRA and REC within a defined timeframe. If a major deviation on a CTIMP meets the criteria for a serious breach, it is notified immediately to the Sponsor and reported to the REC and the MHRA within 7 days of confirmation. Complete investigations of breaches are fully documented, filed in the TMF and a copy sent to the sponsor.

16.-END OF TRIAL

The study end date is deemed to be the date of last data capture (last visit after twelve months of follow up of the last patient enrolled, or death of the last patient, whichever happens first).

The CI and/or TSC have the right at any time to terminate the trial for clinical or administrative reasons.

The end of the trial will be reported to the REC and Regulatory Authority within the required timeframe if the trial is terminated prematurely. Investigators will inform patients of any premature termination of the trial and ensure that the appropriate follow up is arranged for all involved.

Following the end of the trial a summary report of the trial will be provided to the REC and Regulatory Authority within the required timeframe

17.-TRANSLATIONAL RESEARCH

Archival paraffin-embedded tissue will be obtained at study entry for translational research. In addition whole blood, serum and plasma will be obtained at baseline and at 12 weeks for all patients and on disease progression for patients on the chemotherapy arm. The aim of this tissue and blood analysis will be translational research, looking for predictive factors of survival, response to chemotherapy or toxicity. See *Appendix 8*.

Samples must not be taken from a patient who is infected with HIV or Hep C or other transmissible human disease; or from patients who are in high risk groups such as intravenous drug users, as the Manchester Cancer Research Centre Biobank will not accept these samples.

These samples will be collected in the Manchester Cancer Research Centre Biobank; Paterson Institute for Cancer Research; M20 4BX, Manchester.

Analysis of these samples will be subject to a separate grant application and will build on the translational elements integral to other studies in the biliary tract portfolio (ABC-03, ABC-04 and ABC-05).

18.-PEER REVIEW

Peer review of the ABC06 trial has been completed during the consideration of the trial for funding by Cancer Research UK (CTAAC). The trial has been developed within the NCRI Upper GI Clinical Studies Group (HPB subgroup).

19.-PUBLICATION POLICY

The main trial results will be published in the name of the trial in a peer-reviewed journal, on behalf of all collaborators. The manuscript will be prepared by a writing group, appointed from amongst the Trial Management Group, and high accruing clinicians. All participating centres and clinicians will be acknowledged in this publication together with staff from the MCTU. All presentations and publications relating to the trial must be authorised by the TMG and sponsor, on whose behalf publications should usually be made. Authorship of any secondary publications e.g. relating to the various biological studies will reflect the intellectual and time input into these studies, and will not be the same as on the primary publication. No investigator may present or attempt to publish data relating to the ABC-06 trial without prior permission from the TMG and sponsor.

20.-TRIAL RECORD RETENTION AND ARCHIVING

Essential documents are documents that individually and collectively permit evaluation of the conduct of the trial and substantiate the quality of the data collected. Essential documents will be maintained at the MCTU and at the Investigator Sites in a way that will facilitate the management of the trial, audit and inspection. They should be retained for a sufficient period (at least 15 years) for possible audit or inspection. Documents should be securely stored and access restricted to authorised personnel.

APPENDICES

APPENDIX 1: PERFORMANCE STATUS (ECOG)

0: Able to carry out all normal activity without restriction

1: Restricted in physically strenuous activity but ambulatory and able to carry out light work

2: Ambulatory and capable of all self-care but unable to carry out any work: up and about more than 50% of waking hours

3: Capable only of limited self-care; confined to bed or chair more than 50% of waking hours

4: Completely disabled; cannot carry out any self-care; totally confined to bed or Chair

APPENDIX 2: COCKCROFT-GAULT, DuBois AND GLOBULIN EQUATIONS

Cockcroft-Gault for Creatinine Clearance estimation:

Male = $$\frac{1.23 \times (140 - \text{age}) \times \text{weight (kg)}}{\text{Serum Creatinine } (\mu\text{mol/l})}$$

Female = $$\frac{1.04 \times (140 - \text{age}) \times \text{weight (kg)}}{\text{Serum Creatinine } (\mu\text{mol/l})}$$

If the calculated creatinine clearance is less than 30 mL/min at baseline, GFR may be assessed using either Cr51-EDTA or 99mTc-DTPA clearance method to confirm if GFR is ≥ 30 mL/min. If it is not possible to complete Cr51-EDTA or 99mTc-DTPA clearance method or where this confirms baseline GFR is < 30 the patient will be determined ineligible.

The Wright formula is considered an acceptable alternative to Cockroft-Gault, for sites where use of the Wright formula is usual local practice.

DuBois equation for body surface area estimation:

$$\text{BSA} = 0.007184 * \text{Height}^{0.725} * \text{Weight}^{0.425}$$

Globulin equation

TOTAL GLOBULIN = total protein - albumin

APPENDIX 3: NEW YORK HEART ASSOCIATION STAGING (NYHA)

• Class 1: Subjects with no limitation of activities; they suffer no symptoms from ordinary activities.

• Class 2: Subjects with slight, mild limitation of activity; they are comfortable with rest or mild exertion.

• Class 3: Subjects with marked limitation of activity; they are comfortable only at rest.

• Class 4: Subjects who should be at complete rest, confined to a bed or chair, any physical activity brings on discomfort and symptoms occur at rest.

APPENDIX 4: EXPECTED TOXICITY PROFILE OF STUDY MEDICATIONS AND SUGGESTED RECOMMENDATIONS TO PATIENT

**5FU**	
Adverse events	Common: Neutropaenia (nadir: days 9-14; recovery by day 30), Anaemia, Thrombocytopenia, Stomatitis and mouth ulcers, Diarrhoea, Anorexia, Changes in taste, Watery eyes or sensitivity to sunlight, Venous tracking, Nausea, Vomiting<br><br>Less common: Hand-foot syndrome, Discoloration of the skin, Rash or itching, Skin sensitivity to sunlight, Hair loss, Discoloration of nails, loss of nails, Cracking, peeling or excessively dry skin<br><br>Others:<br><br>Cardiovascular: angina, arrhythmia, myocardial ischemia, vasospasm<br><br>Central nervous system: acute cerebellar syndrome, stroke<br><br>Dermatologic: alopecia, dermatitis, dry skin, fissuring, nail changes (nail loss), vein pigmentation, sore mouth<br><br>Gastrointestinal: mesenteric ischemia (acute), sloughing<br><br>Miscellaneous: anaphylaxis, generalized allergic reaction
Recommendations to the patients	Antiemetic premedication<br><br>Loperamide can be used in case of starting diarrhoea<br><br>Drinking plenty of fluids and cleaning teeth regularly might prevent mouth infection and mouth ulcers<br><br>Myelotoxicity is relatively frequent, control of temperature at home<br><br>Increased sensibility to the sun<br><br>Skin creams
Others	Administration to patients with genetic DPD (dihydropyrimidine dehydrogenase) has been associated with prolonged clearance and increased toxicity (diarrhoea, neutropaenia, and neurotoxicity) following administration; rechallenge has resulted in recurrent toxicity (despite dose reduction).
**Oxaliplatin**	
Adverse events	Common: Numbness or tingling of the hands or feet - this condition may be exacerbated by exposure to the cold, Nausea and vomiting,

	Diarrhoea, Fatigue, Anaemia, Thrombocytopenia, Constipation, Changes in liver function tests, liver damage (including rare cases of hepatitis and hepatic failure)<br><br>Less common: Neutropaenia, Fever, Headache, Insomnia, Stomatitis and mouth ulcers, Anorexia, Abdominal pain, Back pain, Abnormalities in renal function tests<br><br>Others:<br><br>Hepatic impairment: The presence of hepatic vascular disorders (including veno-occlusive disease) should be considered, especially in individuals developing portal hypertension or who present with increased liver function tests.<br><br>Peripheral neuropathy, back pain, pharyngeal dysesthesia<br><br>Pulmonary fibrosis<br><br>Reversible posterior leukoencephalopathy syndrome<br><br>Hypersensitivity and anaphylactoid reaction
Recommendations	Take care with extreme drops of temperature, for example, opening fridge/freezers. Avoid drinking iced drinks and eating very cold food for 24 hours before the treatment and for 24 hours afterwards.<br><br>Laxatives can be used if constipation happens

APPENDIX 5: TRIAL TREATMENT: SUGGESTED DOSAGE AND PREMEDICATION

Premedication	Dose	Administration
Dexamethasone sodium phosphate	8 mg single dose	Intravenous bolus injection (day 1)
Ondansetron hydrochloride	8 mg single dose	Intravenous infusion over at least 15 minutes (day 1)
Dexamethasone	4 mg twice a day	Oral, during two days (day 2 and 3)
Ondansetron hydrochloride	8 mg twice a day	Oral, during two days (day 2 and 3)
Metoclopramide hydrochloride	10-20 mg, three times a day if required	Oral, during 7 days (day 1-7)
**Drug**	**Dose**	**Administration**
Oxaliplatin	85mg/m^2	In 250-500 ml of 5% glucose. Two hours intravenous infusion (day1)
L-folinic acid	175 mg (or folinic acid 350 mg)	Two hours intravenous infusion (day1) concurrently with oxaliplatin infusion
5 FU	400 mg/m^2	5-10 min bolus (day 1)
5 FU	2400 mg/m^2	46 hours continuous intravenous infusion (starting day 1, finishing day 2)

In addition to the medications above, the following may be given, according to local practice:

- Thromboprophylaxis for the central line insertion.
- Appropriate treatment for extravasation, if this occurs.

APPENDIX 6: SUGGESTED DOSE REDUCTIONS

	Oxaliplatin (mg/m2)	5FU bolus (mg/m2)	5FU infusion (mg/m2)
Initial dose (100%)	85	400	2400
First dose reduction (administration of 80%, reduction of 20%)	68	320	1920
Second dose reduction (50%).	45	200	1200
Third dose reduction	Not allowed, stop the drug	Not allowed, stop the drug	Not allowed, stop the drug

APPENDIX 7: TRIAL ASSESSMENTS

Assessment	Screening [e]	Trial entry	ARM A[l]	ARM B During treatment with mFOLFOX [h]		ARM B Follow-up (after completion or stopping chemotherapy)	
			Monthly[n]	Day 1 of each cycle	Week 12/ end of cycle 6 [m]	End of treatment [i]	Every month[n]
**Informed Consent**	X						
**Histological or cytological confirmation of inoperable BTC**	X						
**Patient registration/ randomisation**	X	X					
**Medical History**	X						
**Patient demographics**	X						
**Baseline symptoms**	X						
**Height**	X						
**Weight**	X			X	X	X	
**Physical Examination inc. vital signs**	X		X	X	X	X	X
**ECOG performance score**	X		X	X	X	X	X
**FBC**	X		X	X	X	X	X
**Coagulation** [a]	X						
**Biochemistry** [b]	X		X	X	X	X	X
**Estimation of renal function** [c]	X			X	X		
**Pregnancy Test**	X						
**Serum tumour markers** [d]	X		X	X	X	X	X
**CRP**	X		X	X	X	X	X
**Research bloods**		X	Week 12		X		At progression
**Archival paraffin-embedded tissue collection**		X					
**CT scan (thorax/abdo/pelvis)** [f]	X				X	X	
**MR scan (liver) (optional)** [f]	optional				optional	optional	
**Quality of life and health status questionnaires** [g]		X	Every 3 months		X		Every 3 months
**Active Symptom Control**	X --X						
**Therapy: mFOLFOX (Arm B only)** [h]				X			
**Concomitant medication**	X	X	X	X	X	X	X
**Adverse events** [j]	X --X						
**Survival** [k]	X --X						
**Patient diary completion & collection (costs of health and social care)**	X --X						

a PT, INR, APTT, APTT ratio (or usual local practice prior to line insertion- INR is mandatory but others may be omitted if not required per local policy)

b Sodium, potassium, magnesium, urea, creatinine, phosphate, total protein, albumin, globulin, adjusted calcium, total

bilirubin, alkaline phosphatase, AST and/or ALT, LDH, gamma-GT.

c Renal function will be estimated using the Cockroft-Gault formula (see Appendix 2, Wright formula may be used in place of Cockroft-Gault if this is usual local practice). For patients with calculated creatinine clearance < 30 mL/min at baseline, GFR may be assessed using either Cr51-EDTA or 99mTc-DTPA clearance method to confirm if GFR is ≥ 30 mL/min. If not possible to complete Cr51-EDTA or 99mTc-DTPA clearance method or where this confirms baseline GFR is < 30 patient will be determined ineligible.

d CA19-9, CEA and CA125.

e All radiological pre treatment evaluations and other assessments and investigations should be performed within 6 weeks before randomisation and start of chemotherapy (ARM B only).

f CT thorax/abdomen/pelvis (can be Thorax-Abdomen scan followed by a Pelvic scan to provide a 'complete' scan) and optional MR liver within 6 weeks prior to registration. Additional scans for patients in arm B at:

- week 12 of treatment (+/- 7 days)
- at end of chemotherapy treatment (6 months, or earlier where treatment is discontinued).
- repeated every 12 weeks until progression for patients who have not progressed at end of chemotherapy

g EORTC QLQ-C30, QolBil and EQ5D questionnaires.

h Chemotherapy continues for 12 weeks in first instance. May extend to 24 weeks (max, clinician's discretion) or will be discontinued earlier in the case of disease progression, toxicity or patient choice. 1st cycle of chemotherapy should start within 6 weeks of radiological progression from 1st line treatment.

i Includes termination on disease progression or early termination for other reasons (e.g. withdrawal of consent). For patients on ARM B this should be carried out within 30 days of the last treatment dose.

j Follow-up any adverse events or toxicities until resolution or patient death.

k Every month until patient death - may be by telephone if patient's condition precludes hospital visit. For telephone visits the primary aim will be to collect survival status and details of any further active treatment (if applicable).

l All patients must be randomised and patients allocated chemotherapy (ARM B) must start treatment within 6 weeks of diagnosis of radiological progression.

m For patients in arm B the week 12 assessments should be conducted at the end of cycle 6. In cases where a treatment delay has occurred these assessments may also be delayed.

n All monthly visits must be carried out at 4 weekly intervals (+/- 7 days). If there is a change in the dates the following visits can be rearranged so that they are all in sync.

APPENDIX 8: TRANSLATIONAL BLOODS COLLECTION

See Appendix 7 for the timepoints at which these bloods will be collected.

The bloods required for the translational research will be collected as follows:

- 20ml blood in 2 x 10ml EDTA tubes -- plasma
- 10ml blood in 1 x 10ml clot activator tube -- serum
- 3ml in EDTA tubes – whole blood

Please refer to the Laboratory Manual for more information (Section 11 in the Investigator Site File)

APPENDIX 9: FLOWCHART FOR ADVERSE EVENT NOTIFICATION

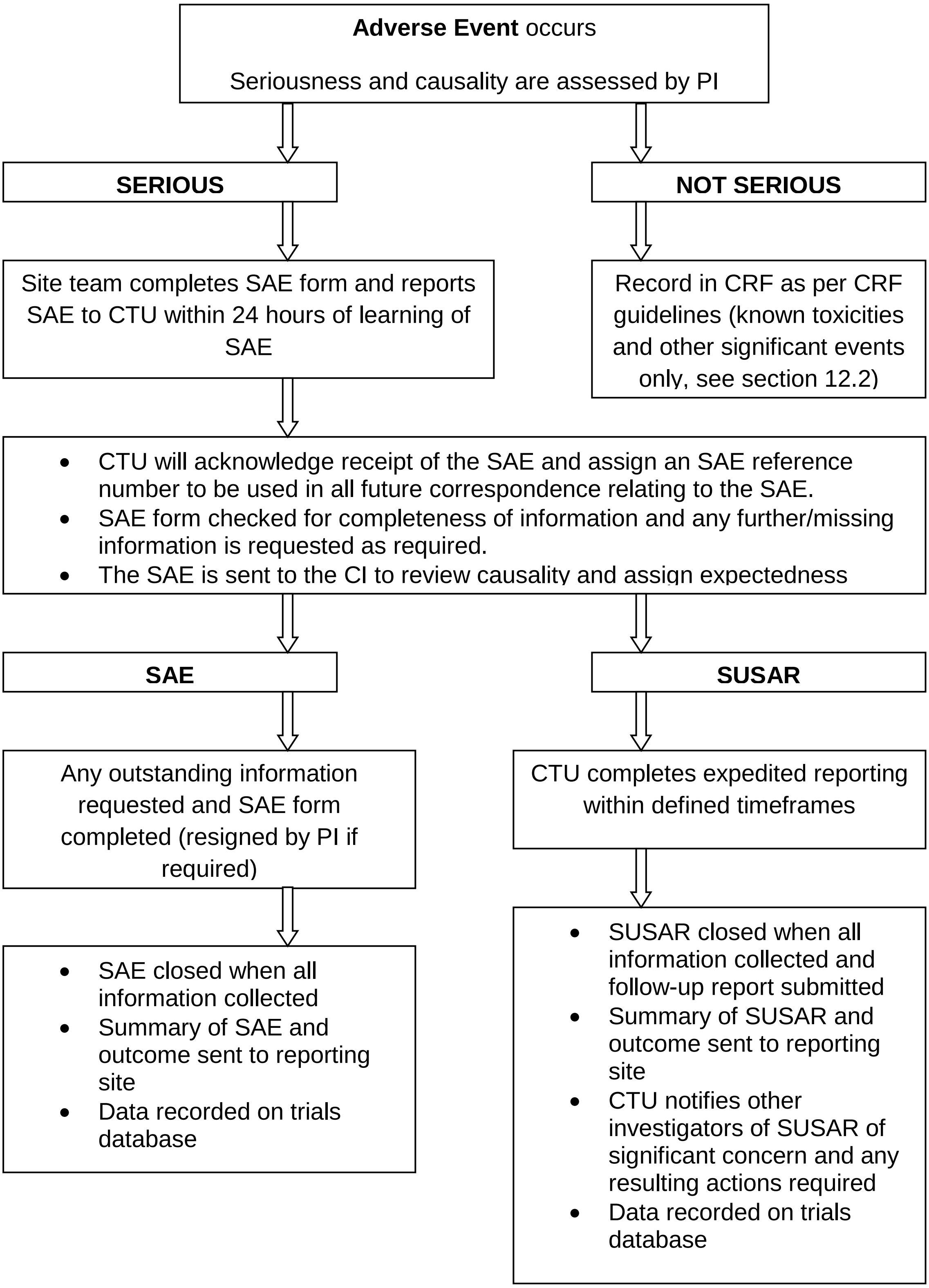

REFERENCES

(1) Cancer Survival Trends in England and Wales 1971 - 1995: deprivation and NHS region.

(2) Cancer Research Campaign Cancerstats: Mortality UK. CRC Factsheet 1999;July.

(3) Gourgiotis S, Kocher HM, Solaini L, Yarollahi A, Tsiambas E, Salemis NS. Gallbladder cancer. Am J Surg 2008; 196(2):252-264.

(4) Gatto M, Alvaro D. Cholangiocarcinoma: risk factors and clinical presentation. Eur Rev Med Pharmacol Sci 2010; 14(4):363-367.

(5) AJCC Cancer Staging Manual, Seventh Edition. 2010.

(6) Eckel F, Schmid RM. Chemotherapy in advanced biliary tract carcinoma: a pooled analysis of clinical trials. Br J Cancer 2007; 96(6):896-902.

(7) Tsavaris N, Kosmas C, Gouveris P, Gennatas K, Polyzos A, Mouratidou D et al. Weekly gemcitabine for the treatment of biliary tract and gallbladder cancer. Invest New Drugs 2004; 22(2):193-198.

(8) Valle J, Wasan H, Palmer DH, Cunningham D, Anthoney A, Maraveyas A et al. Cisplatin plus gemcitabine versus gemcitabine for biliary tract cancer. N Engl J Med 2010; 362(14):1273-1281.

(9) Okusaka T, Nakachi K, Fukutomi A, Mizuno N, Ohkawa S, Funakoshi A et al. Gemcitabine alone or in combination with cisplatin in patients with biliary tract cancer: a comparative multicentre study in Japan. Br J Cancer 2010; 103(4):469-474.

(10) Rao S, Cunningham D, Hawkins RE, Hill ME, Smith D, Daniel F et al. Phase III study of 5FU, etoposide and leucovorin (FELV) compared to epirubicin, cisplatin and 5FU (ECF) in previously untreated patients with advanced biliary cancer. Br J Cancer 2005; 92(9):1650-1654.

(11) Sharma A, Dwary AD, Mohanti BK, Deo SV, Pal S, Sreenivas V et al. Best supportive care compared with chemotherapy for unresectable gall bladder cancer: a randomized controlled study. J Clin Oncol 2010; 28(30):4581-4586.

(12) Bridgewater J, Palmer D, Cunningham D, Iveson T, Gillmore R, Waters J et al. Outcome of second-line chemotherapy for biliary tract cancer. Eur J Cancer 2012.

(13) Walter T, Horgan AM, McNamara M, McKeever L, Min T, Hedley D et al. Feasibility and benefits of second-line chemotherapy in advanced biliary tract cancer: a large retrospective study. Eur J Cancer 2013; 49(2):329-335.

(14) Oh SY, Jeong CY, Hong SC, Kim TH, Ha CY, Kim HJ et al. Phase II study of second line gemcitabine single chemotherapy for biliary tract cancer patients with 5-fluorouracil refractoriness. Invest New Drugs 2011; 29(5):1066-1072.

(15) Lee S, Oh SY, Kim BG, Kwon HC, Kim SH, Rho MH et al. Second-line treatment with a combination of continuous 5-fluorouracil, doxorubicin, and mitomycin-C (conti-FAM) in gemcitabine-pretreated pancreatic and biliary tract cancer. Am J Clin Oncol 2009; 32(4):348-352.

(16) Pino MS, Milella M, Gelibter A, Sperduti I, De Marco S, Nuzzo C et al. Capecitabine and celecoxib as second-line treatment of advanced pancreatic and biliary tract cancers. Oncology 2009; 76(4):254-261.

(17) Sasaki T, Isayama H, Nakai Y, Mizuno S, Yamamoto K, Yagioka H et al. Multicenter phase II study of S-1 monotherapy as second-line chemotherapy for advanced biliary tract cancer refractory to gemcitabine. Invest New Drugs 2012; 30(2):708-713.

(18) Yi JH, Thongprasert S, Lee J, Doval DC, Park SH, Park JO et al. A phase II study of sunitinib as a second-line treatment in advanced biliary tract carcinoma: a multicentre, multinational study. Eur J Cancer 2012; 48(2):196-201.

(19) Buzzoni R, Pusceddu S, Platania M, et al. Efficacy and safety of RAD001 I advanced biliary tract cancer patients progressing after first-line chemotherapy: a phase II study. J Clin Oncol 2010;28 Suppl.; Abstr. e14500).

(20) Roth A, Schleyer E, Schoppmeyer K, Kluge R, Wittekind C, Mossner J et al. Imatinib mesylate for palliative second-line treatment of advanced biliary tract cancer: a bicentric phase II study. Onkologie 2011; 34(8-9):469-470.

(21) Androulakis N, Aravantinos G, Syrigos K, Polyzos A, Ziras N, Tselepatiotis E et al. Oxaliplatin as first-line treatment in inoperable biliary tract carcinoma: a multicenter phase II study. Oncology 2006; 70(4):280-284.

(22) Nehls O, Oettle H, Hartmann JT, Hofheinz RD, Hass HG, Horger MS et al. Capecitabine plus oxaliplatin as first-line treatment in patients with advanced biliary system adenocarcinoma: a prospective multicentre phase II trial. Br J Cancer 2008; 98(2):309-315.

(23) Novarino AM, Satolli MA, Chiappino I, Giacobino A, Napoletano R, Ceccarelli M et al. FOLFOX-4 Regimen or Single-agent Gemcitabine as First-line Chemotherapy in Advanced Biliary Tract Cancer. Am J Clin Oncol 2012.

(24) J.Mane et al. Second-line chemotherapy with capecitabine (CAP) and oxaliplatin (OX) in patients with pancreatic or biliary tree adenocarcinoma (ADC). J Clin Oncol 29: 2011 (suppl 4; abstr 308) 2013.

(25) Lim JY, Jeung HC, Mun HS, Lee DK, Paik YH, Lee SJ et al. Phase II trial of oxaliplatin combined with leucovorin and fluorouracil for recurrent/metastatic biliary tract carcinoma. Anticancer Drugs 2008; 19(6):631-635.

(26) Nehls O, Klump B, Arkenau HT, Hass HG, Greschniok A, Gregor M et al. Oxaliplatin, fluorouracil and leucovorin for advanced biliary system adenocarcinomas: a prospective phase II trial. Br J Cancer 2002; 87(7):702-704.

(27) Walter T, Horgan AM, McNamara M, McKeeve L, Min T, Hedley D et al. Response to letter 'Outcome of second-line chemotherapy for biliary tract cancer'. Eur J Cancer 2012.

(28) Brooks R. EuroQol: the current state of play. Health Policy 1996; 37(1):53-72.

(29) Herdman M, Gudex C, Lloyd A, Janssen M, Kind P, Parkin D et al. Development and preliminary testing of the new five-level version of EQ-5D (EQ-5D-5L). Qual Life Res 2011; 20(10):1727-1736.

(30) National Institute for Clinical Excellence.Guide to the Methods of Technology Appraisal: NICE, 2008.

(31) Briggs AH, O'Brien BJ, Blackhouse G. Thinking outside the box: recent advances in the analysis and presentation of uncertainty in cost-effectiveness studies. Annu Rev Public Health 2002; 23:377-401.

(32) Rawlins MD, Culyer AJ. National Institute for Clinical Excellence and its value judgments. BMJ 2004; 329(7459):224-227.

(33) Fenwick E, Claxton K, Sculpher M. Representing uncertainty: the role of cost-effectiveness acceptability curves. Health Econ 2001; 10(8):779-787.

(34) Sendi PP, Briggs AH. Affordability and cost-effectiveness: decision-making on the cost-effectiveness plane. Health Econ 2001; 10(7):675-680.

(35) Hoch JS, Briggs AH, Willan AR. Something old, something new, something borrowed, something blue: a framework for the marriage of health econometrics and cost-effectiveness analysis. Health Econ 2002; 11(5):415-430.

(36) Briggs AH, O'Brien BJ. The death of cost-minimization analysis? Health Econ 2001; 10(2):179-184.
